# QTL Mapping and Phenotypic Variation for Seedling Vigour Traits in Barley (*Hordeum vulgare* L.)

**DOI:** 10.3390/plants10061149

**Published:** 2021-06-04

**Authors:** Ludovic J.A. Capo-chichi, Sharla Eldridge, Ammar Elakhdar, Takahiko Kubo, Robert Brueggeman, Anthony O. Anyia

**Affiliations:** 1 InnoTech Alberta Inc., P.O Bag 4000, Vegreville, AB T9C 1T4, Canada; sharla.eldridge@innotechalberta.ca; 2 Institute of Genetic Resources, Faculty of Agriculture, Kyushu University, 744 Motooka, Fukuoka 819-0395, Japan; takubo@agr.kyushu-u.ac.jp; 3 Agricultural Research Center, Field Crops Research Institute, Giza 12619, Egypt; 4 Department of Crop and Soil Sciences, Washington State University, Pullman, WA 99153, USA; bob.brueggeman@wsu.edu; 5 National Research Council of Canada, 100 Sussex Drive, Ottawa, ON K1A 0R6, Canada; anthony.anyia@nrc-cnrc.gc.ca

**Keywords:** barley, seedling vigour, QTLs, phenotypic variation, RILs

## Abstract

Seed vigour is considered a critical stage for barley production, and cultivars with early seedling vigour (ESV) facilitate rapid canopy formation. In this study, QTLs for 12 ESV-related traits were mapped using 185 RILs derived from a Xena x H94061120 evaluated across six independent environments. DArT markers were used to develop a genetic map (1075.1 cM; centimorgans) with an average adjacent-marker distance of 3.28 cM. In total, 46 significant QTLs for ESV-related traits were detected. Fourteen QTLs for biomass yield were found on all chromosomes, two of them co-localized with QTLs on 1H for grain yield. The related traits: length of the first and second leaves and dry weight of the second leaf, biomass yield and grain yield, had high heritability (>30%). Meanwhile, a significant correlation was observed between grain yield and biomass yield, which provided a clear image of these traits in the selection process. Our results demonstrate that a pleiotropic QTL related to the specific leaf area of the second leaf, biomass yield, and grain yield was linked to the DArT markers bPb-9280 and bPb-9108 on 1H, which could be used to significantly improve seed vigour by marker-assisted selection and facilitate future map-based cloning efforts.

## 1. Introduction

Barley (*Hordeum vulgare* L.) production ranks as the fourth cereal crop and the earliest domesticated crop used for human food, animal feed, brewing and spirit fermentation for over 10,000 years [1]; therefore, it is of great economic importance. Indeed, it has become an important model monocot cereal crop for molecular genetics and functional studies because it is early maturing, diploid, self-fertilizing and has a short growth period, owing to high-quality genome sequences, and rich in germplasm resources [2,3].

In barley, the seedling stage is considered a critical stage for the plants’ growth and development, including water and nutrient uptake and biotic and abiotic stress resistance, and can influence biomass, grain yield, and grain quality [4,5]. A rapid early development of the leaf area and above-ground biomass, referred to as early seedling vigour (ESV), is recognized as desirable to improve yield under water-limited environments [4,6,7]. ESV determines the potential for the rapid and uniform emergence of plants under a wide range of field conditions [8]. It is mainly expressed as increased seedling weight or height, which usually neglects germination speed [9]. Differences in ESV among cereals have been associated with variation in a number of related traits, such as specific leaf area (SLA), specific leaf weight, leaf area, and the rate of seedling emergence [2,10]. Leaf area can increase without incurring additional costs by decreasing the amount of photosynthetic machinery per unit leaf area. This in turn increases the leaf area per unit weight, measured as the specific leaf area (SLA, leaf area per unit dry mass) [11]. Specific leaf area was suggested to be suitable for selecting plants with superior early vigour. The selection for the width of the first few seedling leaves should integrate embryo size and SLA, and this would be a simple way to screen and select for high early vigour [12]. However, the impact of these complex traits on plant performance is a good indicator of early seedling vigour.

The development of molecular techniques like QTL has become a powerful tool to dissect complex traits and identify chromosomal regions harboring genes that control these quantitative traits, and not only in barley. For instance, Diversity Arrays Technology (DArT) offers a rapid and DNA sequence-independent shortcut to medium-density genome scans of the plant species [13,14,15]. DArT markers have proved to be very useful to detect chromosome substitutions in the breeding program [16]. Since the proof-of-concept report by Jaccoud et al. [13], Diversity Arrays Technology (DarT) has been developed as an inexpensive whole-genome profiling technique for many organisms, especially plants. A current list of organisms which was developed in conjunction with Diversity Arrays Technology Pty. Ltd. is available at www.diversityarrays.com (accessed on 12 May 2021) [17]. Since the first barley genetic map was constructed from a RFLP marker, barley breeders have constructed many genetic maps using various genetic markers, including DArT [18,19,20]. A total of 2032 DArT markers have been mapped to 646 unique positions in the *Hordeum chilense* RIL population [21]. Early seed vigour is a complex trait influenced by multiple genes, which can be dissected into a series of component parts including leaf width, length, area, weight and specific leaf area. Despite the fact that many QTLs have been detected between seedling growth with yield [22], salt tolerance [23,24,25], water logging [26], drought tolerance [27] and nitrogen stress tolerance [28], the QTLs of early seedling vigour-related traits and associated with grain yield and biomass yield have not been well investigated in barely.

The current study aims to utilize the DArT markers for mapping the new QTLs for early seedling vigour-related traits of 185 RILs and evaluate the association between polymorphic markers and the simply inherited loci controlling these traits during two years at three different low moisture environments.

## 2. Results

### 2.1. Phenotypic Analysis

The mean performance values of twelve early seedling vigour-related traits (ESV) are shown in Table S1. Xena showed higher values for leaf length, weight, and dry weight for the first and second leaf, and according to Table 1, no significant differences between the parents were found for SLA1, SLA2, BY and GY. There were significant differences among the RILs for all the traits and means that were assumed to be normally distributed. Considerable transgressive segregation was evident for all early vigour traits in this population. The broad sense heritability (h^2^_b_) estimate varied from 28% in L1 to 35.2% in GY (Table 2). All phenotypic traits showed a continuous normal distribution with skew values ranging from −0.895 to 0.492 (Table 2 and Table S2), which fall between −3 and +3 and are considered acceptable in order to prove normal univariate distribution [29].

**Figure 1:**
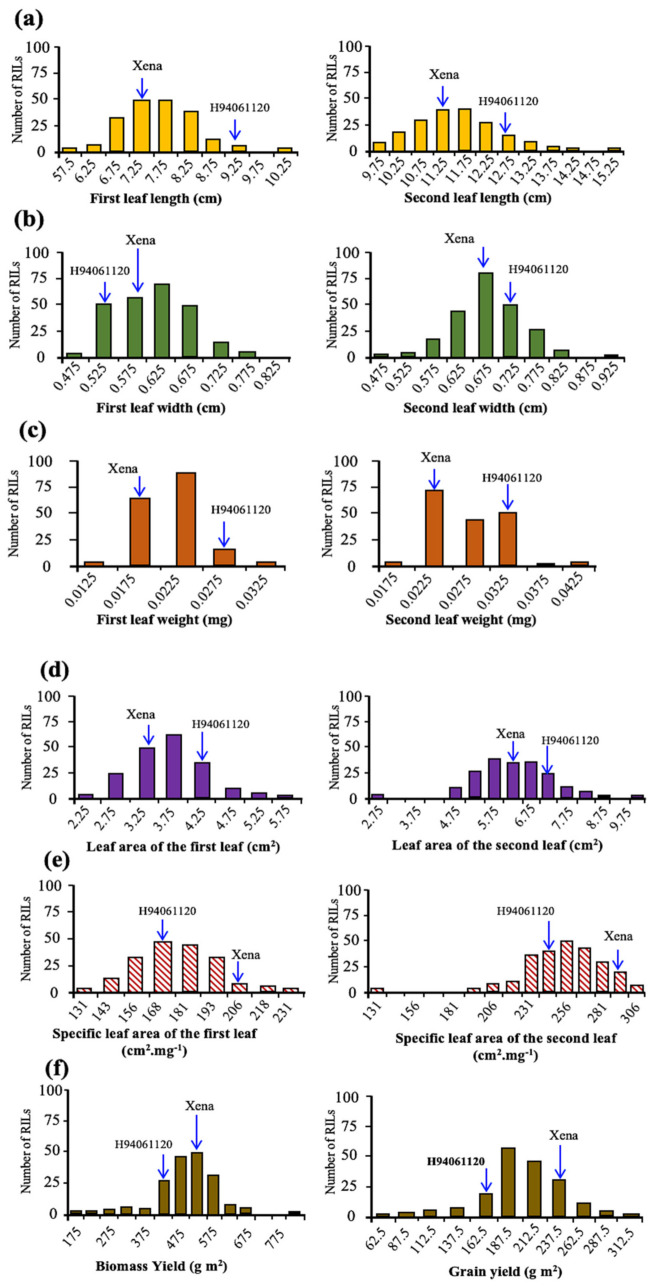
Frequency distribution plots for: (**a**) the first and second leaf length, (**b**) leaf width, (**c**) leaf weight, (**d**) leaf area, (**e**) specific leaf area, (**f**) biomass yield and grain yield in RILs of H94061120 × Xena population. Parental means are indicated for each trait.

The trait phenotypic variances among RILs along with the parental lines showed significant differences in seedling vigour-related traits (*p* < 0.01). The specific leaf area, leaf area, leaf length and weight of the first and second leaves displayed continuous frequency distribution (Figure 1). Nine of the twelve traits considered showed symmetrical distributions, two traits had moderately skewed distribution and one trait displayed binomial distribution. Grain yield (GY) and biomass yield (BY) were slightly skewed to higher values (Figure 1).

**Figure 2:**
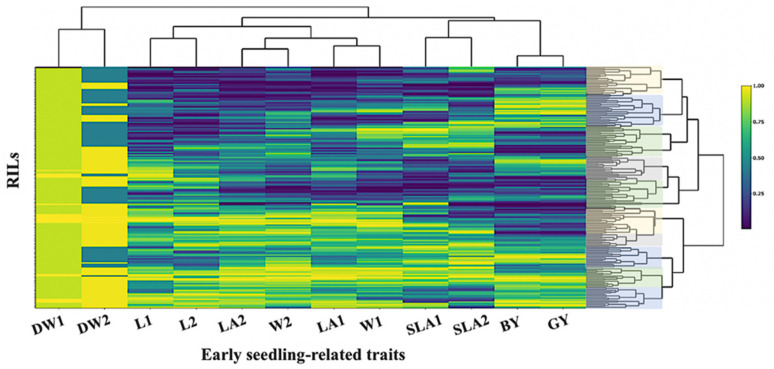
Heat map relationship between the DArT-based linkage map matrix for 185 RIL barley lines. Matrix obtained from 328 polymorphic DArT markers. Rows and columns represent the traits and RILs, respectively.

As the traits were measured in different scales, all data were normalized by subtracting the mean and dividing by the maximal rank, in such a case, bringing each value to its empirical percentile. To confirm that, the genetic grouping of 185 RILs for the twelve early seedling traits (ESV) is presented in a heat map (Figure 2).

**Figure 3:**
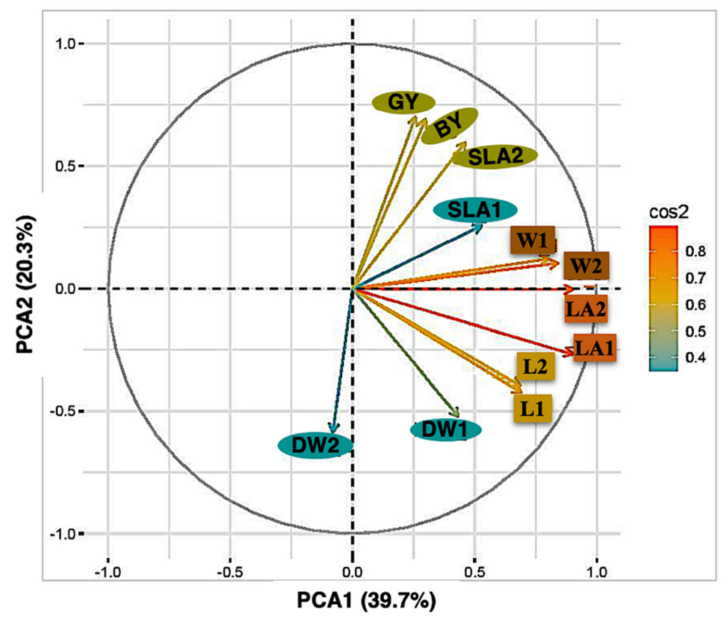
Plot of the first two axes of a principal component analysis.

A clear genetic grouping was detected in the RIL population and ten clusters could be identified. This result might be due to the fact that the RIL population has been developed from the wild accession H94061120 (NSC) and the variety Xena (feed). The high levels of genetic difference between the RILs were also detected by the principal component of early seedling traits. SLA2; GY; BY, W1; W2 and LA1; LA2 were closely correlated across the trials (Figure 3). All the points corresponding to each trait were placed in the same quadrant of the graph of the loadings on the first two principal components. These two components explained 60% of the total variance. However, the DW1; DW2 data points were distributed over two quadrants, indicating changes in the direction of correlations within these traits and between traits. A principal component analysis of these traits, based on the correlations between them, offers a better insight into the relationships within and between traits.

**Figure 4:**
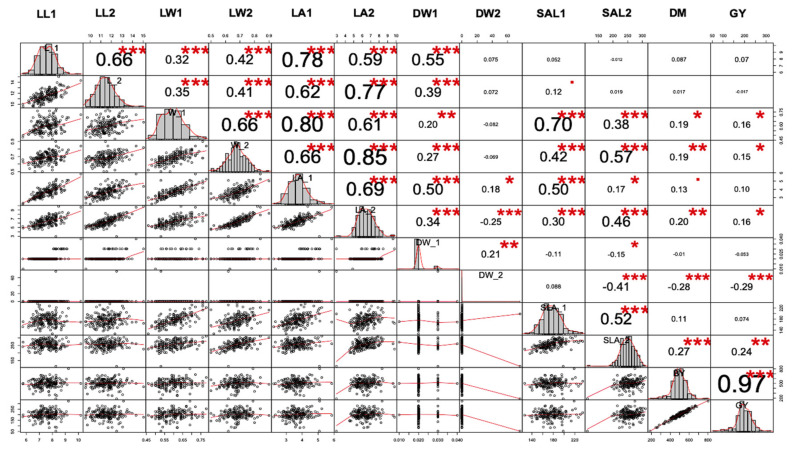
Correlation between twelve early seedling-related traits under low moisture environments. Correlations are displayed to visualize correlation matrix among variables. (▪: not-significant, *, ** and ***, significant level when *p* value: 0.01, 0.05, 0.1, respectively).

The correlation between most of the trait combinations was found to be significant (Figure 4). The strongest positive correlation among traits was found between GY and BY, LA2 and LW2 and LA1 and W1 across all three environments. There was a close relationship between the two traits of the first and second leaves. There was a positive correlation between LA2 and W1. An identical pattern was found between the pairs (LA2–LA1) and W of the second leaf (W2–W1). SLA was positively correlated with LA and negatively correlated with DW, regardless of the rank of the leaves. However, an examination of the relationship between SLA and DW of the second leaf revealed a significant negative correlation, while SLA1 was weakly correlated with DW1. The relationship between SLA1 and LA1 was similar to the one between SLA2 and LA2. An identical correlation pattern was found between leaf area and leaf length of the first and second leaves. A weak correlation was observed between DW2 and L2, while the correlation between DW1 and L1 was significant. SLA2, LA2, L2, and W2 were positively correlated with GY and BY (Figure 4), while a weak correlation was found between the characteristics of the first leaf (SLA1, LA1, L1, W1) and GY, suggesting that the early seedling traits associated with the second leaf could be used to predict grain yield and biomass yield. A significant correlation was observed between GY and BY.

### 2.2. Genetic Map Construction

**Figure 5:**
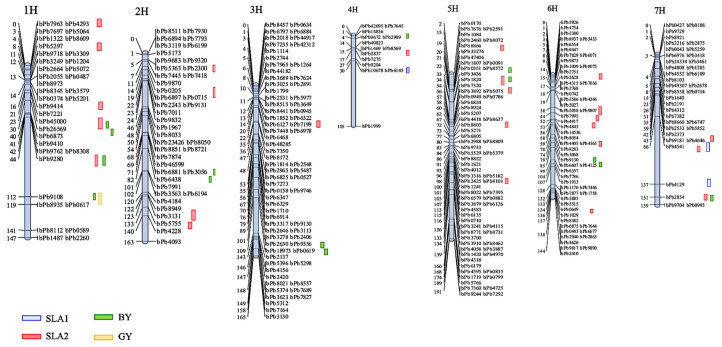
Chromosome location of QTLs associated with 12 early seedling vigour-related traits detected in Xena X H94061120 RIL population by DArT linkage map. Genetic distance scales in centimorgans (cM). The sequences of the relevant DArT markers that are associated with the QTLs in the present study are shown in Figure S1.

A genetic map was constructed using the 328 polymorphic DArT markers across the seven barley chromosomes (Figure 5). The generated map spanned 1075.1 cM distance of the barley genome with an average marker density of 3.38 cM. Each chromosome differed from each other with respect to the total number of markers mapped, total cM distance and marker density. Variation in length varied from 104.9 cM (chromosome 4H) to a maximum length of 191.0 cM (chromosome 5H). The marker density was highest on chromosomes 3H, 5H, and 6H (2.3 cM, 2.7 cM, and 2.5 cM), which harbored 71, 72, and 57 markers, respectively. The lowest marker density was observed on chromosome 4H (7.5 cM) with 14 markers (Figure 5). The numbers of linkage groups per chromosome varied from 2 on Chr. 4H to 12H on Chr.1. The minimum and maximum distances between adjacent markers for each chromosome were 1.6 cM and 58.6 cM in Chr.1, respectively, which indicates that there was a lack of genome coverage in some regions.

### 2.3. QTL Detection and Analysis

A total of 46 QTLs were detected to be associated with four traits (Table 3) in all barley chromosomes, including 35 significant QTLs (LOD > 3.0) and 11 tentative QTLs (2.5 < LOD < 3.0) associated with seedling vigour-related traits. 

Out of 35 significant QTLs, 26 were found for SLA2 with a LOD ranging from 3.06 on Chr. 5H to 7.18 on Chr. 1H and 6H, seven were for BY ranging from 3.01 to 4.18, and two were for GY with a LOD of 3.67 and 3.03. From 26 detected QTLs controlling SLA2, twenty-one, three, one and one were found explaining 18%, 19%, 17% and 16% of the phenotypic variation (PVE), respectively (Table 3). Six of the QTLs identified for BY accounted for 9% of the phenotypic variance, while the remining QTLs captured 18.1% of the variance, respectively. The phenotypic variances explained by the two QTLs for GY were 17.8% and 6.9% (Table 3). As shown in Figure 5, five, four and one QTLs were identified for SLA2, BY and GY, respectively, on chromosome 1H. Moreover, five clusters of QTLs for the traits included three QTLs for SLA2, and one for each of BY and GY traits were located in a similar position on chromosome 1H within the interval (44.0 to 108.0 cM) and shared a common nearest marker, bpb9280. Major QTLs for BY and GY traits were located on chromosome 1H within the intervals 107.8–127.6 cM and 110.2–120.4 cM, and in addition, common QTLs for SLA2, BY, and GY with common markers bpb9108 and bPb9280, respectively. QTLs for SLA2 and BY were found on chromosome 1H within the intervals 43–76.5 cM and 48.1–106.7 cM, respectively, and shared a common marker bPb9280, and QTLs for SLA2 and BY were detected on chromosome 5H within the intervals 33.3–54.2 cM and 34.6–56.7 cM and shared a common marker, bpb1820. Furthermore, one significant cluster was detected on chromosome 7H: QTLs for SLA2 and BY located within the intervals 153–158.7 cM and 134.6–156.8 cM with bPb2854 as the closest marker (Figure 5).

Finally, positive colocations were identified for SLA2, BY and GY with phenotypic variation values ranging from 17.8 to 18.1%. Three additional colocations between SLA2 and BY were detected on chromosomes 5H and 7H with phenotypic variation values of 18.8% and 9.6%, respectively. One positive colocation was identified between SLA2 and SLA1 on chromosome 7H, where the two QTLs exhibited phenotypic variation values of 16.5% and 12.7%. The QTL interval length ranged from 1.6 to 58.6 cM, averaging 15.9 cM. Most of the QTLs detected contained one or two markers. Chromosome 1H is likely to play a key role in seedling vigour and yield determination in barley.

## 3. Discussion 

ESV is a quantitative trait and the QTL mapping for this trait is affected by many factors, including environments, molecular markers, populations, and methods for measuring traits. ESV has a strong influence on plant stand establishment, and the production of high seedling vigour to stabilize crop yield is a challenge for crop breeders. It has been reported that seedling vigour could influence crop yield through both indirect and direct effects [29,30,31,32,33]. However, few QTL mapping experiments in barley have been conducted for studying ESV under low moisture environments using biparental (RIL) population [5]. Our objectives in this study were to detect the QTLs that influence ESV in barley by utilizing the DArT markers and explaining the associations between trait-markers which could be used to improve the water use efficiency and yield stability of barley under low moisture conditions.

### 3.1. Early Seedling Vigour-Related Traits

Twelve ESV traits were characterized that represent indicators of seedling vigour during early development. Significant phenotypic variation was observed across the bi-parental RIL population for seedling growth traits indicative of ESV. We found a negative correlation between the leaf area and leaf dry weight content of the second leaf, suggesting that high or low SLA depends on the significance of the correlation between the two components based on the genotype, species, and environment [34]. An increase in leaf area does not necessarily translate to a proportional increase in dry matter content. This may explain the high specific leaf area observed for the second leaf.

A high specific leaf area would result in greater water loss due to the larger leaf area exposed to ambient air. However, larger leaf areas with greater biomass allocation to the leaves are often associated with a high relative growth rate, which might lower the specific leaf area [35]. Considering that rapid dry matter production during early seedling growth is an important aspect of seedling vigour and the most common measure, high early vigour might coincide with a high relative growth rate in the early stages of seedling development, as suggested by [36]. On the other hand, a positive correlation was observed between the leaf area and leaf dry weight content of the first leaf, suggesting that an increase in leaf area would result in an increase in dry matter content. Leaf traits may reflect the adaptation mechanisms of plants to the environment [37]. Therefore, a lower specific leaf area could be associated with a smaller leaf area, which may be shown to reduce water loss due to evapotranspiration on the leaf surface. However, in some succulent plants with poor seedling vigour that are common in tropical regions, a low specific leaf area may be associated with low leaf dry matter and high leaf thickness. As a consequence of these variations, specific leaf area and its components are often related to each other and to productivity.

Our results confirmed previous findings stating specific leaf area as a suitable trait for the selection of plants with good ESV in cereals [38]. Hence, specific leaf area, the ratio of leaf area to leaf dry mass, is a key functional trait of plants underlying variation in growth rate among species [39,40]. Specific leaf area is also a major trait in the worldwide leaf economics spectrum, which reflects the range of fast to slow returns on nutrient and dry mass investment in leaves among species [40]. We also found that the width of the first leaf is highly correlated with leaf area. It was suggested that the leaf width of the first leaf should integrate embryo size and specific leaf area, and this would be a simple way to screen and select for high early vigour. Seedling leaf width was highly heritable and had a high genetic correlation with total leaf area in wheat during the vegetative stage [41]. Sundgren et al. [42] also showed the importance of both embryo size and SLA in determining vigour among wheat lines. The studied traits L1, L2, LA1, LA2, DW2, BY and GY had high heritability (>30%). These results indicate the possibility of additive gene effect for the expression of these traits. Therefore, selection would be effective for improving these traits.

### 3.2. Map Chatacteristics and QTL Discovery 

The mapping of QTLs related to ESV can enable the dissection of their genetic control and molecular mechanism, leading to the possibility of developing new varieties with improved ESV and enhanced yield. In the present study, QTLs for ESV characteristics were detected using 185 RILs from a Xena and H94061120 cross using 328 polymorphic DArT markers that target gene-rich regions of the barley genome. These markers were distributed across the seven barley chromosomes, spanning a cumulative distance of 1075 cM with an average marker density of 3.3 cM. The genetic map showed extensive genetic diversity which was corroborated by the variation in the analyzed phenotypic traits. The seven chromosomes differed in respect to genetic distances and marker distribution, as a result of which some chromosomes were densely populated (3H, 5H, 6H), while others exhibited few markers (1H, 2H, 4H, 7H). This may be due to the lower recombination frequency in these regions. However, previous analyses of DArT sequences in other species indicated that DarT markers tend to be located in gene-rich regions, which in barley tends to be in the telomeric regions [43]. According to developmental genetics, different QTLs may have different expression dynamics during trait development [5]. Many previous investigations focused mainly on late-growth stages, where analysis was limited to the performance of a trait at a fixed time or stage of ontogenesis [26].

The current study aimed to identify any new ESV-related traits’ QTLs, and we found a total of 46 QTLs—29 were for SLA (26 for SLA2 and 3 for SLA1), 15 for BY, and 2 for seed yield, suggesting that by selection based on SLA, seedling vigour and rapid establishment would correlate with attaining higher yields (Table 3 and Figure 5). We found that most of the QTLs were located on chromosomes 1H, 5H and 6H. It is noteworthy that QTLs for early vigour traits were detected on all seven chromosomes, and that many QTLs controlling multiple traits were located at the same or overlapping marker intervals. For instance, a pleiotropic QTL, detected between DArT markers bPb-9280 and bPb-9108 on chromosome 1H, was related to SLA2, BY, and GY. It may presently be inaccurate to determine whether one gene affects a range of traits or whether there are several genes clustered in the same region that act upon different related traits. Considering all the information here, we suggested that this region may be a credible region for a cluster of QTLs [44]. The co-localization of QTLs for different traits suggests common genetic factors underlying these traits and suggests pleiotropic genetic effects or regulation by tightly linked genes. An assessment of the phenotypic relationships between early vigour and related traits indicated strong correlations between early vigour and grain yield. This implies a pleiotropic QTL or tightly linked QTL in our study. It was suggested that if two QTL peaks are located very close to each other, and the 1-LOD support intervals are completely or mostly overlapped, these two QTLs would be regarded as a single QTL having pleiotropic effects [9]. Several studies also reported QTLs in cereals: 27 QTLs for seed vigour in rye, 27 QTLs were found to be associated with root length, shoot length and shoot dry weight in rice, 29 QTLs were detected for seedling root in wheat and 15 QTLs for root architecture in maize [38,40,41,42].

QTLs for SLA2 were detected on all chromosomes, explaining 16.5–18.8% of the phenotypic variation with the larger contribution from markers bPb5075 and bPb1820 (5H). In a population of fodder barley, Wang et al. [5] reported 70 QTLs over all chromosomes except for 4H explaining 5.01–77.78% of the phenotypic variation, and 23 of them displayed a major effect on 14 seedling-related characteristics. Therefore, the QTL for SLA2 is collocated with the QTL for water content, suggesting that the DArT marker bPb-5075 could be useful for marker-assisted selection (MAS) in barley for breeding for early seedling vigour. Similarly, two QTLs for SLA2 and GY (5H), which peaked at DArT marker bPb-2857, collocated with a QTL for QPSII related to early short-time drought tolerance in barley detected on chromosome 4H and 5H (QPSII.sthb-4H and QPSII.sthb-5H) [45].

In our study, we demonstrated several significant QTL clusters of ESV characteristics in barley under low moisture environments. An alternative explanation for the clustering of QTLs for traits at different organizational levels in the plants is that of a mechanistic dependency rather than a genetic dependency between traits. For example, the colocation of QTLs for SLA2 with QTLs for BY and seed yield might be due to the fact that one or more genes in that region affect SLA2; consequently, this chromosomal region affects the resulting BY and GY. The colocalization of QTLs for SLA1, SLA2 with BY and GY is not unexpected because these traits are associated with photosynthesis and transpiration in barley. Therefore, the relationship among these traits could indicate that these parameters may not be independent but interacting, which may be co-regulated for the protection of the photosynthetic apparatus, an important factor in dry matter accumulation. QTL clustering was repeatedly reported not only in barley [46] but has also been observed in wheat [36], and it was suggested that these QTL clusters represent gene clusters that are separated by regions with noncoding sequences. Marker-assisted selection will become more efficient and effective with the identification of more QTLs that contribute to seedling vigour-related traits. Although many QTLs were identified for SLA1, SLA2, BY and GY seedling vigour traits, there is no significant QTL for leaf area, leaf length, and leaf width. These traits may be complex physiological traits that presumably are under the control of many loci. QTLs with small effects on the overall complex traits are difficult to detect so that for such traits, usually only a few major QTLs are identified [43].

## 4. Material and Methods

### 4.1. Plant Material

A mapping population of 185 F_5_ recombinant inbred lines (RILs) was used to construct a DArT-based linkage map. The RIL population was developed by single-seed descent (SSD), from a cross between a barley variety Xena (feed purpose) and wild accession H94061120 (high in non-structural carbohydrates or NSC). The F_1_ seeds were bulk and grown in-field to generate F_2_, then the plants were evaluated for segregation. Each RIL was selfed to generate F_5-6_ progeny. The parental lines were obtained from the Field Crop Development Centre (FCDC), Lacombe, Alberta, Canada. The Xena and H94061120 genotypes represent a wide range of variation for agronomically important traits. The RILs and parents were evaluated in the field and greenhouse.

### 4.2. Phenotypic Evaluation

#### 4.2.1. Field Experiment Conditions

The RILs and parents were evaluated in three different locations under low moisture environments including Lacombe, Vegreville and Castor in Canada during two successive growing seasons. The soil properties and rainfall for each location are presented in Table 4. The experiment design was a randomized complete block design with six replications. The plot size was four rows, 4-m long. To avoid the border effects, the grain yield (GY) and biomass yield (BY) were obtained from the center rows of each plot. Phenotypic data were collected from six independent environments (two seasons and three locations). An analysis of variance (ANOVA) was performed for the individual trials, and then the least significant difference test (LSD) was used to determine the differences among the genotypes.

#### 4.2.2. Greenhouse Evaluation of Seedling Vigour-Related Traits

**Figure 6:**
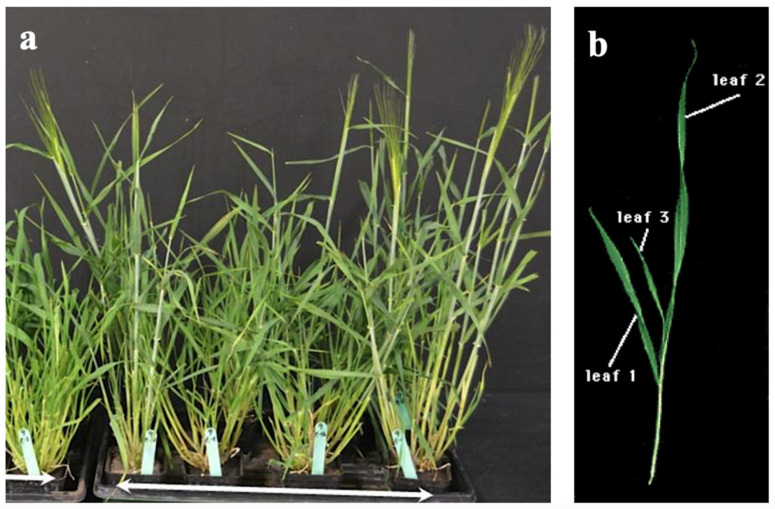
Phenotypic evaluation of barley 185 F5 RILs and two parental lines. (**a**) All genotypes were evaluated in 6-inch pots at the greenhouse with four replicates. (**b**) Measurement of leaf length and width of the first two mainstem leaves.

All genotypes were evaluated in 6-inch pots in a completely randomized design (CRD) with four replications (Figure 6). The grown seeds were selected to have similar size or weight. Twelve early seedling vigour-related traits (ESV) included leaf length (cm) and width (cm), leaf area (cm^2^), leaf dry weight (mg) and specific leaf area (cm^2^·mg^−1^). The recorded data and methods of measurement were as follows. Early seedling vigour was assessed at the third leaf stage by checking the presence or absence of the coleoptile tiller, the first leaf length (L1), the second leaf length (L2), the first leaf width (W1), and the second leaf width (W2). Leaf area of the first (LA1) and second leaf (LA2) was measured according to the method described by [12]. The first leaf dry weight (DW1) and the second leaf dry weight (DW2) were determined after drying in the oven at 70 °C for 48 hours. Specific leaf area of the first leaf (SLA1) and the second leaf (SLA2) was recorded as the ratio of leaf area to dry weight of the first two mainstem leaves as follows:

SLA = *A*/*M_L_*

where *A* is the area of the first or second leaf of an RIL plant, and *M_L_* is the dry mass of those leaves_._ Embryo size for all genotypes was calculated on the basis of [31].

#### 4.2.3. Estimation of Genotypic Parameters 

The trait values for each RIL were reported as the average of values from five plants in each replication. Analysis of variance (ANOVA) was performed to estimate the genetic variation for the measured traits among the RILs, using the general linear model. The R packages “*PerformanceAnalytics*”, “*cos*2” and “*heatmaply*” were used for computing, correlations, principal component analysis (PCA) and heat maps, respectively, for all traits across the environments. Heritability in the broad sense [47] was estimated for all traits. 

Heritability in broad sense

h^2^_b_ = V_G_^2^/V_P_^2^ × 100

where, V_G_; genotypic variance and V_P_; phenotypic variance

#### 4.2.4. Genotyping and Construction of Genetic Linkage Map

Young leaves from five-week-old plants (ten seedlings per genotype) were collected and total genomic DNA was extracted using DNeasy Plant Mini Kit (Qiagen, Hilden, Germany), then quantified at 230 nm and qualified at 230/260 and 260/280 absorption ratios, respectively. The isolated DNA for 185 RILs and parents was sent to Triticarte to perform DArT analysis [48]. Genotyping was performed according to the standard barley DArT® array by Triticarte Pty Ltd., Canberra (http://www.diversityarrays.com/, accessed on 12 May 2021). A quality parameter Q, which is the variance of the hybridization intensity between allelic states as a percentage of the total variance, was calculated for each marker. Only markers with a Q and call rate greater than 80% were selected for linkage analysis. Polymorphic loci were selected after discarding those with a minor allele frequency of 0.5, a missing value of more than 20%, or a common position. The linkage analysis was conducted using a Kosambi mapping function within the JoinMap 4.0 [49], with a recombination frequency of 0.25, and all markers were grouped among the seven chromosomes. Haldane’s map function was used to calculate the recombination rate of the genetic distance in cM.

#### 4.2.5. QTL Analysis

QTL analyses were performed for each of the twelve phenotypic traits across all environments. Out of the 953 DArT markers [14], 328 polymorphic markers were mapped. All data included in the linkage map were used. To estimate the marker trait associations, composite interval mapping (CIM) was performed using QTL Cartographer v2.5 [50]. Logarithm of odds (LOD) threshold score for QTL (*p* = 0.05) was determined using a 1000-permutation test by shuffling the phenotype means with the genotypes. A LOD score of 2 indicates that the model containing the estimated QTL effect is 100 times more likely than the model with no QTL effect. A LOD threshold score of >2.5 at 1000 permutations was considered significant to identify and map the QTLs in the barley population. The 95% confidence intervals of the QTL locations were determined by one-LOD intervals surrounding the QTL peak [51]. Composite interval mapping is based on the idea that the residual error term in a QTL analysis is the within genotypic class variance. This residual variance is partly due to experimental error but may also be due to variation caused by the segregation of another QTL outside of the region being tested. To reduce the background genetic segregation variance when conducting interval mapping, CIM first uses regression analysis to choose a subset of markers that have the biggest effects. These are used as “cofactors” in a subsequent interval mapping. When testing positions near a cofactor, that particular cofactor is dropped from the model, so that the QTL effects in that region can be more precisely identified.

## 5. Conclusions

This study identified 46 significant QTLs for ESV-related traits, of which 26 were detected for SLA2, 15 for BY, 3 for SLA1, and 2 for yield. QTLs for yield harboured two QTLs for SLA2 and two QTLs for BY. QTLs controlling SLA2 were distributed on all the seven chromosomes and explained on average 16.5–18.8% of the phenotypic variance. We found that SLA2 may contribute to ESV in barley and could be used to improve early seeding vigour, plant establishment, and seed yield. We found that most of the QTLs were located on chromosomes 1H, 5H and 6H. It is noteworthy that QTLs for early vigour traits were detected on all seven chromosomes, and that many QTLs controlling multiple traits were located at the same or overlapping marker intervals. A pleiotropic QTL was detected between markers bPb-9280 and bPb-9108 on chromosome 1H related to SLA2, BY, and GY.

## Figures and Tables

**Table 1:** Means squared for the parental lines “Xena” and “H94061120”.

Traits	Xena	H94061120	LSD (0.05)
L1	9.31 ^a†^	6.68 ^b^	1.69
L2	12.88 ^a^	10.43 ^a^	3.35
W1	0.59 ^a^	0.52 ^a^	0.2
W2	0.75 ^a^	0.59 ^a^	0.81
LA1	4.61 ^a^	2.83 ^b^	1.52
LA2	1098 ^a^	7.68 ^b^	9.32
DW1	0.126 ^a^	0.073 ^b^	0.01
DW2	0.150 ^a^	0.093 ^b^	0.06
SLA1	176.17 ^a^	190.99 ^a^	86.96
SLA2	263.25 ^a^	376.94 ^a^	283.61
BY	561.33 ^a^	523.23 ^a^	275.91
GY	234.23 ^a^	212.33 ^a^	182.78

L1: the first leaf length, L2: the second leaf length, W1: the first leaf width, W2: the second leaf width, LA1: leaf area of the first leaf, LA2: leaf area of the second leaf, DW1: the first leaf dry weight, DW2: the second leaf dry weight, SLA1: specific leaf area of the first leaf, SLA2: specific leaf area of the second leaf, BY: biomass yield, GY: grain yield. ^†^: Means within a row with the same letter are not significantly different according to the Least Significant Difference (LSD) at a 0.05 probability level.

**Table 2:** Genetic parameters among twelve early seedling vigour traits in RILs of “Xena” and “H94061120” cross.

	L1	L2	W1	W2	LA1	LA2	LW1	LW2	SLA1	SLA2	BY	GY
Mean	7.55	11.56	0.60	0.68	3.67	6.34	0.02	0.02	175.96	255.40	499.57	200.83
Standard Deviation	0.712	0.956	0.063	0.065	0.597	0.991	0.003	0.003	18.886	25.963	84.083	38.350
Skewness	0.238	0.395	0.391	0.038	0.492	0.361	0.783	0.783	0.401	−0.895	−0.372	−0.528
Range	4.47	5.49	0.32	0.40	3.65	6.88	0.02	0.02	108.47	198.40	616.45	269.45
Minimum	5.72	9.55	0.46	0.5	2.21	2.94	0.013	0.013	126.4	112.36	186.2	53
Maximum	10.19	15.04	0.78	0.90	5.86	9.82	0.04	0.04	234.87	310.76	802.65	322.45
CV	4.93	4.22	6.48	5.40	9.67	8.35	7.82	8.31	7.11	5.37	22.04	25.50
h^2^_b_ %	28.0	28.4	28.0	29.2	30.4	32.8	29.7	31.0	28.3	28.7	32.3	35.2

L1: the first leaf length, L2: the second leaf length, W1: the first leaf width, W2: the second leaf width, LA1: leaf area of the first leaf, LA2: leaf area of the second leaf, DW1: the first leaf dry weight, DW2: the second leaf dry weight, SLA1: specific leaf area of the first leaf, SLA2: specific leaf area of the second leaf, BY: biomass yield, GY: grain yield. Min: minimum, Max: maximum, CV: coefficients of variation, h^2^_b_ %: heritability in broad sense.

**Table 3:** Major QTLs for seedling vigour traits detected in the RIL population across two years.

Trait	Chr.	Nearest Marker	Position (cM)	Interval (cM)	LOD	Additive Effect	Phenotypic Variation
SLA1	4	bPb6145	37.1	27.8–64.0	2.57	22.91	16.1
	7	bPb4541	70.9	65.7–100.5	2.50	25.43	12.7
	7	bPb4129	130.9	100.5–136.5	2.56	25.05	13.1
SLA2	1	bPb4293	2.0	0.6–3.2	6.31	−75.91	17.8
	1	bPb5297	8.7	7.0–10.7	6.15	−75.91	17.8
	1	bPb9414	18.1	17.1–18.7	6.54	−75.91	17.8
	1	bPb45000	26.5	22.8–31.0	6.97	−75.91	17.8
	1	bPb9280	45.5	43.0–76.5	7.18	−75.91	17.8
	1	bPb9280	106.5	79.7–108.7	6.55	−75.91	17.8
	2	bPb2300	6.2	3.8–7.0	5.76	−75.91	17.8
	2	bPb0205	16.4	9.5–19.0	6.53	−75.91	17.8
	2	bPb3131	127.7	122.0–132.8	7.11	−75.91	17.8
	2	bPb5755	136.3	133.4–140.3	6.78	−75.91	17.8
	3	bPb7199	16.7	13.9–20.2	6.78	−75.91	17.8
	4	bPb2837	15.6	12.6–20.2	6.14	−75.91	17.8
	5	bPb8866	17.3	17.0–21.9	3.06	−73.41	16.8
	5	bPb1820	36.8	33.3–54.2	7.56	−77.73	18.8
	5	bPb5075	55.1	55.1–60.5	5.48	−77.73	18.8
	5	bPb8803	69.8	66.2–75.7	4.15	−75.91	17.8
	5	bPb8101	97.5	94.0–103.5	5.74	−77.40	18.6
	6	bPb2628	16.2	12.6–19.0	5.91	−75.91	17.8
	6	bPb9807	41.0	19.0–44.3	6.69	−75.91	17.8
	6	bPb7995	47.5	44.9–52.5	7.06	−75.91	17.8
	6	bPb4843	53.6	51.8–58.8	6.21	−75.91	17.8
	6	bPb4565	64.2	59.4–66.7	7.18	−75.91	17.8
	6	bPb0649	134.1	128.3–138.4	4.22	−75.91	17.8
	7	bPb9181	42.7	41.1–44.9	3.63	−75.91	17.8
	7	bPb4541	67.9	60.7–73.3	5.79	−72.67	16.5
	7	bPb2854	154.6	153.0–158.7	6.93	−75.91	17.8
BY	1	bPb45000	26.5	24.1–28.6	2.69	179.14	9.6
	1	bPb26569	34.9	31.2–38.6	2.76	179.08	9.6
	1	bPb9280	75.5	48.1–106.7	3.65	−78.13	18.1
	1	bPb9108	112	107.8–127.6	4.18	178.09	9.5
	2	bPb3056	74.8	68.3–77.8	2.66	178.42	9.6
	2	bPb6438	93.1	84.1–103.7	3.09	177.77	9.6
	3	bPb48285	40.9	36.8–46.0	3.02	181.41	9.9
	3	bPb9336	104.8	100.5–109.4	2.67	179.06	9.6
	3	bPb0619	138.8	110.0–144.1	3.05	178.36	9.6
	4	bPb2909	8.7	3.8–15.8	2.86	178.92	9.6
	5	bPb8572	25.8	20.0–35.2	2.95	178.67	9.6
	5	bPb1820	36.8	34.6–56.7	3.01	179.08	9.6
	6	bPb9130	79.6	74.0–84.7	2.70	178.95	9.6
	6	bPb4125	91.8	84.7–100.0	2.94	178.85	9.6
	7	bPb2854	150.6	134.6–156.8	3.60	179.19	9.6
GY	1	bPb9280	74.5	48.4–106.2	3.67	−35.69	17.8
	1	bPb9108	112.0	110.2–122.4	3.03	69.50	6.9

**Table 4:** The soil properties and rainfall for each location.

Locations	Lacombe	Vegreville	Castor
Soil texture *	OBLC	EBCM	DBC
Precipitation (mm) †	440 ± 84	382 ± 62	340 ± 89
Latitude/Longitude	52°280 N/113°440 W	53°34 N/113°31 W	52°1300 N/111°5306 W

OBLC; Orthic Black Chernozem soil, EBCM; Eluviated Black Chernozemic Malmo soil, DBC; Dark Brown Chernozemic soil. The three sites were considered three different environments characterized by distinct soil moisture conditions with Castor as the driest site and Lacombe as the wettest site. * Source: Canadian system of soil classification. †: The average annual precipitation source: (Agro Climatic Information Service; ACIS).

## Supplementary Materials

The following are available online at https://www.mdpi.com/article/10.3390/plants10061149/s1, Figure S1: The sequences of the relevant DArT markers that are associated with the QTLs in RIL population derived from the cross between Xena X H94061120. Table S1: Mean performance for twelve early seedling vigour-related traits of 185 RILs of barley. Table S2: The normal distribution values the four twelve early seedling vigour-related traits.
