# Supplementary material for: QTL Mapping and Phenotypic Variation for Seedling Vigour Traits in Barley (Hordeum vulgare L.)
Source: Plants (Basel). 2021 Jun 4;10(6):1149. doi: 10.3390/plants10061149 (PMC8227620; doi:10.3390/plants10061149)
Supplement: Supplementary file 1 [file plants-10-01149-s001.zip › Supplementary Figure S1.pdf]

Chr. 1H

BPB-7963
TGCAGTACCATACTTTCCTCCAAATAGTTCTTCTTTTGCATGTCCTTGAAATTCCTTGTTTCCAAAAGACACTAATGTTG
GAATAAACCACGATGCGTGGCCTTGTCCGGCTGGGACTCGGTGCAATCACCTAATAGTACTAGGATTAAGAATTGGTAAC
CGTATAAGTTTACTTGTTTGATGATAGCTGCTTTATATACGTAGGTATCCTGTAATATTATGAACCAAAATCAAAGCAAT
ACAAAGCACAGGACCGACACAGCCGTGTTCGCCATCACCTCGAGTTTTCGCGTGTATGTTCTGTTGTGAATGAATTAGCG
CCGGCGAGATGATCGCTAACTTAGCTGATTAAGCTAGCTACTCGTGTGTGGGTGTCCAGCAGCACTCGATCCACCTATGT
ATCTGCTTGCTTAGTACTACTACGTGTGTATTTGATTAGTGTGGTAATATATTGCACGTACGTGTCGACTCGTCAAGGAA
CCGACGTGCAACATCTGATCGGGTCTGTGACAACAATTGGTATCTAGAACTTGATTTGTTTGCGTGATTTTCGGAAAGTA
ATCGAAGAATCATGTCAAACCCGGGCAAGGAGATTGTCGTGGCGGGAGCGGGAGCACCGATGCCGATGGTGGGGGTGTCT
CAATTCCCCCGACTGGATCGAGAGGACTACGCACTATGAGCGATGAACATGGAGGTCGCGATGGAGGGCGCTGAGTTCTG
GTAAGCTATCGATCCCGGTGGAGCCCAGTACACGAAGGGTGCGGCGAAGTACCGGACGGGTCGTCAGGCGTTAACGGTGA
TCTACTCCGCCATGCCGAAGGATGTGCTGCA

BPB-4293
TGCAGGACGAGGGGCACCGGGAGGGGAAGGGGGTGGAGCCCCCGCAACTGGTCGCGATGGCATAATATGTCCGCCGTCTC
CGAATGTTGGCAAGTATGTCGTCGGATGATCCCATGGCGCTTGAAGCCACGGCCACGGGAACATATGTCCACCATCACAT
GGCGGATGCATCGGCTGGGGAGGGCACCATTGTGGTTGCATAGGTTGGGGCATTGCCGGCGGTAGCAACCATGATGGTGG
ATGTGGCCAGCCCCCACCCCCAGCCCCCCCTTGTGATTCTAAGTCTTCCGCTGATGAGGGGTCTTCATTGTGATTGATCC
AATCACCAGTTCCATTGCCCGGATCTTCTGTATCACTGGATTCCTCTTTTTCATCTGGACCTTCTTTGCCAATCTCAAAC
GGGCCAACATGCTCTACCATAATCCATACGATGGTAATTAGGTCACCGCCACGGCCGAGTTGCCTCGCATGGGATTCTCT
GCTGCATCGGGTGGCTGCATACAACAGCTTATCCACCCAAATCTTGAAGACGAGCTCCAAAAGTTTCGGCATCTTCAAAC
CATCAGCCTTGAGCAGCTCCAGAGCAATATTAGCACCGTCTGAGACCAGACGAGTTCTACTCTTACCTAAACCCCACTCG
TCGTCTCCCTTCTTGTTCTTTGCCAGCATCCTTGCAAGCTTCTCCTCTGCA

BPB-7697
TGCAGTTCTTGGATGCTTTGCAACTTACCAAAAGTTTTTGGTATTGATCCCGATATTTGGTTCTGAAACAAGCCTAAATA
TTCGAGGTTCATCAGATCGCCAATTTCCTCGGGGATGGAACCGGTTATTTGATTTTCAAAGAGGTATAGTACTACTAGCT
TGGTAACATTTCCTAAGCTGTCAGGAATAGAGCCAGATATTTGGTTGATGGACAAGTCTAAATTCTGGAGATTCATTAGA
TTGCCAAACTCTCTGGGGATGGAACCAGATATCTTATTTTCAAAGAGGTGTAGTAGTAATAGCTTGGTAATATTTCCTAA
GCTGTCAGGAATAGAGCCAGATATTTGGTTGTCAGCCAAGTCTAACTTCTGGAGATTCAGCAAGTAGCCCAATTCTGAAG
GTATTGTGCCTGTGATATGATTTGTATAGAGAAGAAGTTCATTGAGCATAGTGAGGTTGCCTACTTCTGCCGGTATTGAA
CCTGTGATTTGATTTCTATAGAGACCAAGTTGGTTTAGCATAGTGAGGTTGCCTATTGCTGGGGGTATTGAACCTGTGAT
TTGATTTTCAAAGAGAAAAAATTGGTTCAACTTTGTAAGATTGGTTATGAAGACTGGAATTGGACCTGACAAATCATTTC
CAGCAAGATGAAGATTTTGCAAATGGACTAGCCTGCCTAGTTCTTGGGGTATAGGCCCTGAAAGTTGATTATGAAACACG
TACAAAGTATTTAGTTGGGTCAGATTTCCAAGGGTTTTTGGTATCATGCCGCCTAAGGTGTTGTTGCTTAGCTGTAGAAG
TTGTAGGTTGACAAGCCTTCCAATCTCTTCAGGAATGGGACCTGATACCATGGTTTGGTGAATGATAAGATCAGTTAACA
TTGTTAGGTTACACAGAGACACAGGGATATGTCCTGTGAGTTTGTTAAATGAGAGACCAAGTTGGGTGAGACTCTGCA

BPB-5064
TACGGGTCGATCAAGTTAGGGTTCGCTTGTCCTTGTTTGGTTCTCGGCCATGGCGGCGCTAAAGGGCCTCGTCGCCAGCA
CCCACACGGCACCATGCTACCGAGACATCTCGTGCAGGGCTGCCGAACGACATCGTCGCCGGTGGCCAGTCGGACTGCTC
GACCATGGCAGCCGCTCGCCATGTTGTTGGCCGGGCGCACTCCTCGACGGTGGCTATTCATGCGCCAACATGTCATCGCG
GGCAGCGGGCCAACTCCTCGTGGACGAACGGACGCGCACCAAGACCCTTCGAAGTGCTCCATTTTGTCGCACTTGCTCAA
GCCGGCGCCGGTGTACTCCATGAGACCATGACACTTACGTGATCAGGCTGATTGTAAGACGCATGCTTACATATTCAGCA
TATACATGTGCATTGTCTAGTTTGGTTGATCGATTCCTTTAGTCTCATGCTAGCCCTTGATTAGATTAGTATGATGCTTG
GCCAAACTGTTATATCATTAGTTCAATCTGTAAATACTTAAGATAATATCTTTTTTTTTTGCAGGTGAAGATAATATTGT
TTTCAAAACACTATTCGGCGATAGGATTTTAATGAAGGAACTAGTACAAGCAGGCTCCAGTGATGACTTGGAGCACTTCA
AAAAGGAGGCAACTGCA

BPB-1322

BPB-8609
TGCAGCAGATGTATCAAAGGGCAGTGAGCATATGTGAATGGTAGAACAGAAATGAACTAACAAAAGAGAATACGGTGTAG
CCATCTCAGCAACATGCACCACTAGGAGTAAAGAAGGGAGGCTTCATATATTATTGAGAATTCGGCATGAATGCATTTAT
CATGAAAGACTTGCTACATTAGTTTGCCTCGTCAGGAAACTTATTTGTTCAGTACCACAAAGTTGGAGCTACCAATGACA
TGTTTGCTAGATCAGTTTGTCGTCACGCAACATGGGTCAGAGCATACCTCATAAAGCTTCGTCAAGTAAATCCTTACGAT

TGGTCATTCCAATAAGCAACACGTTATTCAACGCCTCACACCGTCTATCTGTAAATTTGA

BPB-5297
CCNCTAGTAACGGCCGCCAGTGTGCTGGAATTCGCCCTTTGGATCCAGTGCAGCATGCCAGCCAGATAAAAATGGTTTAT
TTCCTGTACACATTGCTGCCATGATGAATAGTAGAATTGCCATCCATGTTCTGCTCACACTATACCCTCGTTGCATCGGA
TTTCCTGATAACCAGGGAAGATCCTTCCTTCATATTGCTGCCCAGAACAAGAACTCCCTTGTAGTAAGATATGCTTGCAG
TGTATCGTGTCGGGATTTTGGATCGATAATGAAGTCGATAATGAACGCTCAGGACGACGATGGCAACACCGCACTACATC
TAGCTGTGGATGCTGGGGATCTATCTTCTTTCTGTGCACTGCTGGGGAACCGAGAAGTATTATTAAATATCAGAAATAAC
AAGAATCAAACTCCACTGGATTTGGCATGGAGCAAGAGGAAAGGTTTTGCGTACGGATGGGTATTAATCTATATTTCCTC
TCTCATTTCCTCATGTTAGTTCGTCATATAGCTAGAAGATGTATCCCAGTATCCCACTTCATTTTTTTCAGTAAATATAT
ATCCCGCATCTTGATGATTTTCTTGCAGAATCCGGAACATGTGATGTACAGAACTCTTGTACGTGCCGGCTCTAGTCATG
CTAGCTTTTGGAGAGATCGTCTTCAACAACTTTGCAATTTAGTGCCACTAGAAAGTCAAGAGGATGAAGGGAAAGATCCA
GCAAAAGAAAAAAAAGAGGATGAAGAGAAAGAAGAGTCAGCTAAAGTGACAGATTCAACTCGGACACTTGGCATTGGCTC
GGTACTCATAGCATCAGTGACATTCACTGCAACCTTCACCGTACCTGCACTGGNTCCATCAAGGGCGAATTCTGCANATA
TCCATNNNNCCTGGCNNNNNNTCGAGCATGCATCTAGAGGGCC

BPB-9718
TGCAGCGATAGCCCTTACGGGAGCTATGGCGGCAAGACATCTTTTACGTGGTACTGGTCCGGGGAGGAGGATGAGGATGA
CCTTCCTAATGGGTTCCAATGGAGGGACGAGCCTCGACCGAACAAATCAAAGGAAAGAGTTTGGAACGAGAGCGATGTGG
ATGAGGAAGAGGCCCCTCGTCGCGATGATCTGAAAAGCCATAGGATATCCCTTGGCTTGCCAGCCTTGGGTCCCTTAAAG
CTTGATCATATCAAGTCCGCGTAAGTCTTTGAGCATCTGCATAGCATTTGCGAGGATTCTGATAATCGGGCTCCTGATTG
GTGTTCTCTTTCTCGATGCTACGTTTCTGCA

BPB-3309

BPB-3249
TGCAGAGGTGTTCGTTAGCTATTTGCAGTTAGAGAGTGCTCAATATAAGACCTGCATGCCTTTTCTGAATATAGCAGATT
TTTTTTCTCAGCAACGAGGTAAATGAAGTGCACTGCCAAATAAAGCATTTAAACTAATTTTAGTGCTGACCACACTGAGC
TGGTGGTTAGTACCAGTTGAAGGTTGTGTTTAGACTTATATCCTTGGGACAGAATGGAAGGCAAATGGAAAACAAATTTT
GGGTTAAACCGGACAGAGGGAAAAGAAAAATGAACAGCCCACAAGACAATGAGTATCTGTTCTTTTTTTAGGCAATTGGC
TAAGAATTGTGCATTGTCTACAACTGCAAGCAAAGCAAAGACTATACTAAGAGTCTCGCATCGTCTCCGGCTGCAAGCAA
AGCAAAGGGAAGCTAGCAGAAACTTGGCATGCCCTGCA

BPB-1204

BPB-2664

BPB-5072
TGCAGAAATGCACGCACAAAGAAATCAAGTTACATGCTAATCCAAACATCAGCTCTAGCTCTTGTCAAACGGAGTTCATT
CATAGAAGAGTCTTACAAATTGAAGGCAGACATCACATTTCGGGTGGTGTAGCTCCTTGTAGCACAACTTGTGGTATGGC
TCTGCGCCTAGCAAGGTGAACTGGAAACACAAAGATAGAGAAAACGGGTGACGGTCACTTTCTATTTCAATGGATCTGTA
GCTCAAGGCTTAAAGGGAGTGTAGGATGGTCACCTCAGTCTCACGGATAGGGTGGGTGCAGGAAGAGCAACGGAAGCACT
GAGGATGCCAGTACATCCCCATGCAGCTCAAGTAATGGCCATGTCCAACCTCATGCTTGCAGCCACCACAGACCCTGCA

BPB-2055
TGCAGCATACCAACATGATAAGAATGGTTTGTTACCTGTACATGTCGCTGCCTTGATGCATAGGAAAGTTGCCATCCTTA
TTCTCCTCGAAAGATGCGGTGGTTGCCTCGCTCTTCCTGATAAGCAGGGTAGATCCTTTCTTCACATTGCTGTCCAGAGT
GAGGCCTATGGTATAGTATGATATGCTTGTCAAGAACCNGTTTTTGGGCCGATACTCAACGCTCGGGACAATGATGGCAA
CACCGCATTACACCTAGCTGTGGAGGTTGAGGATTTACTCATCGTCTGTTATCTCTTGCGGAATCCAAAAGTATTATTAA
ATGTAAGAAACAACAAGGATCAAACTCCGCTGGATCTAGCAAGGAACAAGACTCATCTCACAGATTTTTCCTATGGACTG
GTATTAAATCTAGCTTCCGTTCTCATGCATGCTTCATGCTAGTTGATCTTATAAATGGAAAGATAAAACTATATTTCTTC
ATTATTGTGTTTTCAGAATCCGGAGAACGCAATATACGAGACACTCAGGGATGTCGGAGCTAGGCATGGTAGCTTCTGGA
GTGATCATGTTCAACAACTCTGCATTCAGAATCAGTTGTCAAACCTAAAGGGTGAAGAGAATCAATCAGAAGAAGTGGAT
GATTTTGAGATGCATAAGAAGAAAAAGGATGAAGAGAATCAATCAGAAGAAGTGGATGAGTTGGAGAAGCATACGGAAAA

AAAGGATAAAAAGAAAAAGGAAGCGAACCGTAAGAAAAAAGAGTATGAAAAGAAAAAAGAGGTGAACCGTAAGAAAAAAG
AGGAAGAAGAGAAAAAAGACTCAGATCAAATGAATGATTCAACCCGGACACTTGGCATTGGCTCGGTACTCATCACAACG
ATGACATTCGGTGCAACCTTCACAGTACCTGCA

BPB-0487
CCAGTGCAGGGCATGCCAAGTTTCTGCTAGCTTCCCTTTGCTTTGCTTGCAGCCGGAGACGATGCGAGACTCTTAGTATA
GTCTTTGCTTTGCTTGCAGTTGTAGACAATGCACAATTCTTAGCCAATTGCCTAAAAAAAGAACAGATACTCATTGTCTT
GTGGGCTGTTCATTTTTCTTTTCCCTCTGTCCGGTTTAACCCAAAATTTGTTTTCCATTTGCCTTCCATTCTGTCCCAAG
GATATAAGTCTAAACACAACCTTCAACTGGTACTAACCACCAGCTCAGTGTGGTCAGCACTAAAATTAGTTTAAATGCTT
TATTTGGCAGTGCACTTCATTTACCTCGTTGCTGAGAAAAAAAAATCTGCTATATTCAGAAAAGGCATGCAGGTCTTATAT
TGAGCACTCTCTAACTGCAAAATAGCTAACGAACACCTCTGCACTGGATCCNTCA

BPB-8973
TGCAGCATACCAACATGATAAGAATGGTTTGTTACCTGTACATGTCGCTGCCTTGATGCATAGGAAAGTTGCCATCCTTA
TTCTCCTCGAAAGATGCGGTGGTTGCCTCGCTCTTCCTGATAAGCAGGGTAGATCCTTTCTTCACATTGCTGTCCAGAGT
GAGGCCTATGGTATAGTACGATATGCTTGTCAAGAACCAGTTTTTGGGCCGATACTCAACGCTCGGGACAATGATGGCAA
CACCGCATTACACCTAGCTGTGGAGGTTGAGGATTTACTCATCGTCTGTTATCTCTTGCGGAATCCAAAAGTATTATTAA
ATGTAAGAAACAACAAGGATCAAACTCCGCTGGATCTAGCAAGGAACAAGACTCATCTCACAGATTTTTCCTATGGACTG
GTATTAAATCTAGCTTCCGTTCTCATGCATGCTTCATGCTAGTTGATCTTATAAACGGAAAGATAAAACTATATTTCTTC
ATTATTGTGTTTTCAGAATCCGGAGAACGCAATATACGAGACACTCAGGGATGTCGGAGCTAGGCATGGTAGCTTCTGGA
GTGATCATGTTCAACAACTCTGCATTCAGAATCAGTTGTCAAACCTAAAGGGTGAAGAGAATCAATCAGAAGAAGTGGAT
GATTTTGAGATGCATAAGAAGAAAAAGGATGAAGAGAATCAATCAGAAGAAGTGGATGAGTTGGAGAAGCATACGGAAAA
AAAGGATAAAAAGAAAAAGGAAGCGAACCGTAAGAAAAAAGAGTATGAAAAGAAAAAAGAGGTGAACCGGAAGAAAAAAG
AGGAAGAAGAGAAAAAAGACTCAGATCAAATGAATGATTCAACCCGGACACTTGGCATTGGCTCGGTACTCATCACAACG
ATGACATTCGGTGCAACCTTCACAGTACCTGCA

BPB-8345
TGCAGGGCATGCCAAGTTTCTGCTAGCTTCCCTTTGCTTTGCTTGCAGCCGGAGACGATGCGAGACTCTTAGTATAGTCT
TTGCTTTGCTTGCAGTTGTAGACAATGCACAATTCTTAGCCAATTGCCTAAAAAAAGAACAGATACTCATTGTCTTGTGG
GCTGTTCATTTTTCTTTTCCCTCTGTCCGGCTTAACCCAAAATTTGTTTTCCATTTGCCTTCCATTCTGTCCCAAGGATA
TAAGTCTAAACACAACCTTCAACTGGTACTAACCACCAGCTCAGTGTGGTCAGCACTAAAATTAGTTTAAATGCTTTATT
TGGCAGTGCACTTCATTTACCTCGTTGCTGAGAAAAAAAAATCTGCTATATTCAGAAAAGGCATGCAGGTCTTATATTGAG
CACTCTCTAACTGCAAATAGCTAACGAACACCTCTGCA

BPB-3579
TGCAGGGCATGCCAAGTTTCTGCTAGCTTCCCTTTGCTTTGCTTGCAGCCGGAGACGATGCAAGACTCTTAGTATAGTCT
TTGCTTTGCTTGCAGTTGTAGACAATGCACAATTCTTAGCCAATTGCCTAAAAAAAGAACAGATACTCATTGTCTTGTGG
GCTGTTCATTTTTCTTTTCCCTCTGTCCGGTTTGACCCAAAATTTGTTTTCCATTTGCCTTCCATTCTGTCCCAAGGATA
TAAGTCTAAACACAACCTTCAACTGGTACTAACCACCAGCTCAGTGTGGTCAGCACTAAAATTAGTTTAAATGCTTTATT
TGGCAGTGCACTTCATTTACCTCGTTGCTGAGAAAAAAAAATCTGCTATATTCAGAAAAGGCATGCAGGTCTTATATTGAG
CACTCTCTAACTGCAAATAGCTAACGAACACCTCTGCA

BPB-0374
TGCAGAGGTGTTCGTTAGCTATTTGCAGTTAGAGAGTGCTCAATATAAGACCTGCATGCCTTTTCTGAATATAGCAGATT
TTTTTTCTCAGCAACGAGGTAAATGAAGTGCACTGCCAAATAAAGCATTTAAACTAATTTTAGTGCTGACCACACTGAGC
TGGTGGTTAGTACCAGTTGAAGGTTGTGTTTAGACTTATATCCTTGGGACAGAATGGAAGGCAAATGGAAAACAAATTTT
GGGTCAAACCGGACAGAGGGAAAAGAAAAATGAACAGCCCACAAGACAATGAGTATCTGTTCTTTTTTTAGGCAATTGGC
TAAGAATTGTGCATTGTCTACAACTGCAAGCAAAGCAAAGACTATACTAAGAGTCTTGCATCGTCTCCGGCTGCAAGCAA
AGCAAAGGGAAGCTAGCAGAAACTTGGCATGCCCTGCACTGGATCCNTCAAGGGGCNAATTCTGCAGATATCCATCANAC
CTGGGCGGCANGCTCGAGCATGCATCTNGANNNCCAATTCGCCCTATAGTGAGTCGTANTACAATTCACTGGCCGTCGTT
TTACAACGTCGN

BPB-5201
TGCAGATGTACAAAATGCCGTCATCAACCATATGTGTGGGCAGCACGGTCACATCATAGTGGCAAGGCAAGAGCAGCAGG
CCAGCACCCAGAAAACACAGTAGTTCCACCTTCCCTGTCTAATCGCGGCAAACACGGTCACAGTCGTAGAAGTGATGGAT
AATTTGATCCTTTTAAGAATGTATTTTGTCTGATCCCTCCCTCGACATTACCAACCGGCAAGGCAGCTTGTTTTTATCCG
AGAGATTATTAGTCTTTGACGCAAGTTGGTGATTATTTGAAGAGATGAACACAGCTAGCTGCCGTCGATGATCCATAGCT
GCGGTAGATTATTTTTAAAAACTCAATCTAAAACCTAATCCCAGTCGGCACTGCTACTGTGCTTGTTTATAGTTTTTCAA
GCGAGGCCGGATCTTGGACATTGATTTCTTGCATCACCACTTCACCAGTATATTACTTGTATGGCATTAGTCATTAGAA

ACCGTAACTATACATGCATACGTACCGGTGCAGTTGTGAGCTTGACGGGCAACCGCTCCAAATGATGATTAGAGGGAGAG
GACGATGCTGCACTGCTGCTTCCGCCTCTGTTTGTCGCGTACTTAGCGTATGTACGTACGTTCTTCTTCAAGTTAAAAAC
ACAATCTCACAGATTCGGGTCTTGGTCTCTTGCAATAAGCATATTCTAGGAACACATACCACGACACGACAGATAGCAGT
TCCAAAAAATATATTAAAACTATTATTCTGACTTCGTACTTAACTTGCTAACGAACTCAAACTGATTCGTCTCTTTTCTA
TATTTCACCTGATTTCGGACGCCCGGCTACAACCCGCTCGGTGGTGGCAGCCTGCA

BPB-9414
TGCAGCATACCAACATAATAAGAATGGTTTGTTACCTGTACNNGTCGCTGCCTTGATGCATAGGAAAGTTGCCATCCTTA
TTCTCCTCGAAAGATGCGGTGGTTGCCTCGCTCTTCCTGATAAGCAGGGTAGATCCTTTCTTCACATTGCTGTCCAGAGT
GAGGCCTATGGTATAGTACGATATGCTTGTCAAGAACCAGTTTTTGGGCCGATACTCAACGCTCGGGACAATGATGGCAG
CACCGCATTACACCTAGCTGTGGAGGTTGAGGATTTACTCATCGTCTGTTATCTCTTGCGGAATCCAAAAGTATTATTAA
ATGTAAGAAACAACAAGGATCAAACTCCGCTGGATCTAGCAAGGAACAAGACTCATCTCACAGATTTTTCCTATGGACTG
GTATTAAATCTAGCTTCCGTTCTCATGCATGCTTCATGCTAGTTGATCTTATAAATGGAAAGATAAAACTATATTTCTTC
ATTATTGTGTTTTCAGAATCCGGAGAACGCAATATACGAGACACTCAGGGATGTCGGAGCTAGGCATGGTAGCTTCTGGA
GTGATCATGTTCAACAACTCTGCATTCAGAATCAGTTGTCAAACCTAAAGGGTGAAGAGAATCAATCAGAAGAAGTGGAT
GATTTTGAGATGCATAAGAAGAAAAAGGATGAAGAGAATCAATCAGAAGAAGTGGATGAGTTGGAGAAGCATACGGAAAA
AAAGGATAAAAAGAAAAAGGAAGCGAACCGTAAGAAAAAAGAGTATGAAAAGAAAAAAGGGGTGAACCGTAAGAAAAAAG
AGGAAGAAGAGAAAAAAGACTCAGATCAAATGAATGATTCAACCCGGACACTTGGCATTGGCTCGGTACTCATCACAACG
ATGACATTCGGTGCAACCTTCACAGTACCTGCA

BPB-7221
TGCAGCCGTAGCCGCCGTCTTGTCTGCCTTCATGAGCAATTTCTTATCACATTATTTGCACTTTTGCAAAAAGTTCGGCG
AGACCATCCTCGTCTGTAACTAATTCTTCATCGTAGTACCTCCAAATGGTAGAGCATCTCCAACGAGTGTTTTGATTTGG
ATTAGACATGTGTGTGGAAAATGCGATGGAGTTTGAGTGTGAGAAATGAGAAAGTGAGCATCCTTTTTTATAGCAAAAAA
TGGCACGTTAGGCACATTAGACAACAATAATGCATAAAATAAAAAATGAGGTAATGTATCATCTGGGATTAATCTTGAGC
TTTATTCGTGTCTACCTCATTAAATCCTCCCATTCCTGGGAAGTAAAAGAATACAAAGCCACGCTCTAGAATTACAAAAG
GGAAATAAAATTACAAAAAGATAAGAAATTTACAAAAGAAAAAGAAAAGGACAACACTACTATTCTTCCCGTTCTTCTTC
TTCAAGGTTCATTTCTTCAAACATGGGATCTTCTGCA

BPB-45000

BPB-26569

BPB-6873
TGCAGAAAGTTAATATCACGTATTCTGTTAAAAACTAAATCATTTCTGCAATTCTATAGAGCCCACAAAAGAGCACATGC
TCCAACACGAATATGTTTGGCCAAATAAACATCCACTCCCTGTAAAAGGCAAATGATTATTATGATTGTTCCTTATTTAT
CTTTCGGCATCAGCTAGTATGTAAATTGAACATCATTCTTTTTGGCAACAGACGCTAGTGAGGAAAATACTCTAAACTGG
GAGCAACGGTACAACATCATTCTTGGAATTGCCAAGGGAATACTGTATCTTCACGAGGACTCAATCCCAAGGATAATCCA
CAGGGACCTTAAAGCTAATAATATTCTTCTAGACGAGGAGATGGATCCTAAAATCGCAGACTTTGGATTGGCAAGGCTGC
TACAAGAAGGTCACACCCATACTCAAACCACTAGAGCTGCTGGAACACTGTAAGTCAAATGCCTATAATTAGGTTTGCAA
AAACGAAAAAGTTACTTGTAATTATGATCTATATCACTTAGTGAAGGCCCGTTTCCAAGCCTAATTTCCCATCCTCTGCA

BPB-9410
TGCAGATGTACAAAATGCCGTCATCAACCATATGTGTGGGCAGCACGGTCACATCATAGTGGCAAGGCAAGAGTAGCAGG
CCAGCACCCAGAAAACACAGTAGTTCCACCTTCCCTGTCTAATCGCGGCAAACACGGTCACAGTCGTAGAAGTGATGGAT
AATTTGATCCTTTTAAGAATGTATTTTGTCTGATCCCTCCCTCGACATTACCAACCGGCAAGGCAGCTTGTTTTTATCCG
AGAGATTATTAGTCTTTGACGCAAGTTGGTGATTATTTGAAGAGATGAACACAGCTAGCTGCCGTCGATGATCCATAGCT
GCGGTAGATTATTTTTAAAAACTCAATCTAAAACCTAATCCCAGTCGGCACTGCTACTGTGCTTGTTTATAGTTTTTCAA
GCGAGGCCGGATCTTGGACATTGATTTTCTTGCATCACCACTTCACCAGTATATTACTTGTATGGCATTAGTCATTAGAA
ACCGTAACTATACATGCATACGTACCGGTGCAGTTGTGAGCTTGACGGGCAACCGCTCCAAATGATGATTAGAGGGAGAG
GACGATGCTGCACTGCTGCTTCCGCCTCTGTTTGTCGCGTACTTAGCGTATGTACGTACGTTCTTCTTCAAGTTAAAAAC
ACAATCTCACAGATTCGGGTCTTGGTCTCTTGCAATAAGCATATTCTAGGAACACATACCACGACACGACAGATAGCAGT
TCCAAAAAATATATTAAAACTATTATTCTGACTTCGTACTTAACTTGCTAACGAACTCAAACTGATTCGTCTCTTTTCTA
TATTTCACCTGATTTCGGACGCCCGGCTACACCCGCTCGGTGGTGGCAGCCTGCACTGNTCCATCAAGGGCNAATTCTGC
NNANATCATCNCNCNNTGNCNGNNNNTCGAGCATGCATCTAGAGGGNCCAATT

BPB-9762

NACGGCCGCCAGTGTGCTGGAATTCGCCCTTGATGGATCCANTGCAGGAGAAGCAAGAAGAAGCCCAACACGACTTCCGG
TACAAGCGACGGTGAAAAAAGGCCACGCCCGCACCCACGCCCTACAAAAAAACATGCGTCGCTCGACCTGAGGCCCACCT
CATTCTCCATGACAATGGTGACCCACACTGGCACCACACGCTGACCAAGGGCTTCACTTGGTGAATGACCCAGACAAAAT
TTAGAGACACAATCCAAAATCAACAACCATAATTGGCTGTGAAGCCTTAAAATTTAGGGAGCTTAGTGTTATAGAAGATG
TAGCACCTCCGATCAATATAAACAAAGGTTTATTCTCAACTAGGTAACAAAGTGTTTCATCCTACCTCGTCATCAGTACG
TAAAACGGAAATTTTCACTCCCAGACTCAAAAACCATTCTTGCTGATCCAGTTGCATTACATGCGGCAAGTTAGCATATA
TCCGGATCAAGATTACCAGCTACTTTTGCTTGGGGTGCTGCA

BPB-8308

BPB-9280

BPB-9108
TGCAGGTTTAACTCAAGAAGACACCAATGCCAAAGTATCTATGTCAACCAAGACTAATTCTACAGAACTATTAGCAATCT
CCAGACCTTTAGGCAGCTCACATTATTACGCGTGCATCAACAAGTTCAGTTCTACTCCCAATCCTGCTACAAGCCGGGTA
GTAAACGGGCGTTTTANCTTTTTTTCATTTTGCAACGAGTGCGTTTTACGCTCTGTCCCGTGCTTGCAGTACATAAATGA
GATTTCCTCAATTATCTGCCTTATTTTTACTTTGTAAAGGCCCTTTATCTACTTAAACCCTTTAGCTTAGGACCAGATTA
ATTAGGATATAGTGAACAATGTTATGCACGCCATTTTAGGTTTTGCTGCCCGGTAGGAGACTAGCTAAAGCTCTAGTAAA
ATCAGTAGTGCTTGATGAAGATTCAGAGAACACCAGAATCATCTTTAAGCATAGTAACTGCA

BPB-8935

BPB-0617
TGCAGTAATTCCGAAGATTTGTTATTTACGAAGTAAAATGAACAAGCCTTCTCATATTGGGGGATTCCTTCCTATTTGGG
GAGTAAGTTTTTCCCATATTCCAATTTTTAGTTTTTTTTATCTCAATTTTCATTTTTTCTATATATTTTTTGACTGATTG
AGTTTTTATTTTGGAATGCCAGATCATTTATCTGTATTTTGTTGATTTTGATGTTATTGAGAATCATCAATCAACTGTGG
ATTATTCTCTTCCTCGGATAACTCATATTAAGAATGATGATTTCCAGTATCTTGCATTGGTCGATATGAATTATGAGTCT
AAGAAAGCTTTTGCAGTTCTATCAGTAAGCATCGTATAATATATTTCATGTGTTTACTTATACTTTCAGCTCCGGTATTT
TTTCATTGTTCATTCTTCTTTCATTTGTAGCTTCGAGACATATCTCAAACACCCTATGCTAATGTTGGGCTTGTGAACAA
TGCTCCTGTTAATCCTTCCGAGTTTCTTTCAGTTCCTCATGTGGAGGTATGTCTTTTCCTTCATTCAGATTTCATTGAAT
ACATGATTTTCAACTAGTCTCTTGTTTTTTTGCACTATAGTTTAATATTTATAGATAATGTCACTTTTAGGTCATTAATG
CCATGAATGTTGACTTAGATGGCAATGTGAAGCCAGATCCTCATTCCAAGAAACAAGTAAATTTGAATATTTTCCCCCTC
GTACAATATTGTTCATCATTTAAAGCAAATTTGTTCTGCA

BPB-8112
TGCAGATGTACAAAATGCCGTCATCAACCATATGTGTGGGCAGCACGGTCACATCATAGTGGCAAGGCAAGAGCAGCAGG
CCAGCACCCAGAAAACACAGTAGTTCCACCTTCCCTGTCTAATCGCGGCAAACACGGTCACAGTCGTAGAAGTGATGGAT
AATTTGATCCTTTTAAGAATGTATTTTGTCTGATCCCTCCCTCGACATTACCAACCGGCAAGGCAGCTTGTTTTTATCCG
AGAGATTATTAGTCTTTGACGCAAGTTGGTGATTATTTGAAGAGATGAACACAGCTAGCTGCCGTCGATGATCCATAGCT
GCGGTAGATTATTTTTAAAAACTCAATCTAAAACCTAATCCCAGTCGGCACTGCTACTGTGCTTGTTTATAGTTTTTCAA
GCGAGGCCGGATCTTGGACATTGATTTTCTTGCATCACCACTTCACCAGTATATTACTTGTATGGCATTAGTCATTAGAA
ACCGTAACTATACATGCATACGTACCGGTGCAGTTGTGAGCTTGACGGGCAACCGCTCCAAATGATGATTAGAGGGAGAG
GACGATGCTGCACTGCTGCTTCCGCCTCTGTTTGTCGCGTACTTAGCGTATGTACGTACGTTCTTCTTCAAGTTAAAAAC
ACAATCTCACAGATTCGGGTCTTGGTCTCTTGTAATAAGCATATTCTAGGAACACATACCACGACACGACAGATAGCAGT
TCCAAAAAATATATTAAAACTATTATTCTGACTTCGTACTTAACTTGCTAACGAACTCAAACTGATTCGTCTCTTTTCTA
TATTTCACCTGATTTCGGACGCCCGGCTACAACCCGCTCGGTGGTGGCAGCCTGCA

BPB-0589
TGCAGGCTGGGCCCAGCCGCCACCGAGCGGGTTGTAGCCGGGCGTCCCAAATCAGGTGAAATATAGAAAAGAGACGAATA
CTGATTGGTACTTCAGTTTGAGTTCGTTAGCAAGTTATAAGTATGAAGTCAGAAAAAAAAATTATTTCTTTTTTGCAACTG
CTATCTGTGGTGTCGTGGTATATGCTTATTGCAAGAGACCAAGACCCGCGCGAACCAGTGAGATTGTGTTTTTAACTTGA
AGAAGAACGTACATACGCTAAGTGCGCGACAAACAGAGGCGGAAGCAGCAGTGCAGCATCGTCCTCTCCCTGTAATCATC
GTTTAGAGTGGTTGCCCGTCAAGCTCACAACTGCACCGGTACGTATGCATGTATAGTTACGGTTTCTAATGACTAATGCC
ATACAAGTAATATACTGGTGAAATGGTGATGCAAGAAAATCAATGTCCAAGATCCGGCCTCGCTTGAAAAAATATAAACA
AGCACAGTAGCAGTGCCGACTGGGATCAGGTTTCAGATTGAGTTTTAAAACATAATGTATGGGTTGCACTGAGATATTCG
AAGGCCCACAAGCTAGCTACTGTGCTCGGCCGTCTGGATGCTCATGCATGACCGCCGTGATGGTGACGGTGCAGCTATGG
ATCATCGACGGCAGCTAGCTGTGTTTATCTCTTCAAATAATCACCAACTTGCGTCAAAGACTATTTTCAAATAATCTCTC
GGATAAAAACAAGCTGCCTTGCCGGTTGGTAATGTGGAGGGAGGGATCAGACAAAAATACATTCTTAAAAGGATCAAATT

ATCCATGACTTCTACGACTGTGACCGCGCTTGCCGATTAGACAGGGAAGGGGGAACTACTGTGTTTTCTGCTGGCCTGCT
GCTCTTGCCTTGCCACTATGATGTGACCGTGCTGCCAACACATATGGTTGATGACGGCATTTTGTACATCTGCA

BPB-1487

BPB-2260
TGCAGATATCTGAACTTGAAAAAGTGAAGTCCCCAAGAGAGGCACATCGTATAAAATTAGTGGAAAAACATAGGCTCAAG
GACTTGAAATTGGAGTGGACCAGAGGTGTTGAGAGATTTGTCTATGACAAAATGTTGTTGAAAAATCTAGTGCCGCCAAG
CACATTGAAGAATTTGGAGATATGTGGTTACAATGGTGTGAGCTTTCCTGCGTGGCTAGTGGGACAACTACCAAACCTAG
TCTCATTGGTTCTCAGAGATATGGAAAATGTGGAAGAGTGGAACATGTCATACTCCATTGATGAGGACCATGTGATTCAA
ACATTGGAAATACATGGTTGTTCCATGTTAAGGATGAAACGACCTCTTCCTAAAGCTAAATCATGGGTGATAGCACACAG
TGATAATGTATTATCATCATTGGATGAGTGCACTGTGTCACACACCAGTGCTTTCCCCTGCACTTCTCCGATAACTACCG
TGTTGTTGTCGGTTAAATACTGCAAGGGGCCTTATCGGTGGAGGTTACTTCAACACTTCACTGGACTCTCTTCTTTGTCA
ATTCACAACTGCGGTGATCTAACTGGATCACCAGCGATCACCCATCATCTATCGTCTCTCGAGGCACTATGCCTAGAAGA
CGAACATATGGAAGAACTGCCAAAATGGATGGGTGAGCTCAGATCACTACACAGTCTGGAAATAAGGTGGAGCAATGTTG
TAACAGAGTTAAATGAGACAATGAGGAAACTCACAATGCTGCA

CHR. 2H

BPB-8511
TGCAGCGAAGAAGATGTAGCATAGAGTCATGCAGTTCGCTGGAAGTGTGTTGAAGCCATCCACACCCAATATCGCCATGT
TTCGGCACACAATGGCATATGGTGCTGGGTATTCGGTGCCGCTTGTACCGATGTTATGTATATCGAATCTGATTAGTTAT
TATGGACGAACAATGCATATTAGAGCCAATGTAAATTCAGTGAAAGCAAGTAGATTATAAGTACAATGAAATTTGTCACA
TGCTCATGCAATCAAATGGTAGAGCAACAACTAGTCCACTAATCACAAGTAATAGACATAACTTATTTAAAAAGGTCAGT
TCTTCCTTCCTAACACCAAAACCCTTTGACCGAGCATATGCATTATAGTAAATGTAACCTTCTTCCTCGCTGCCAAAAAT
TTGACTGATCACCATGTCATATTCTGCAATGTTCTCGAGGGTGGCACTCCCATATTCCATGTCAAACCCTGCAATTAAAA
AGAATCTAATCTAAGTTATTTGAATTCCACCCATGAAACTAAAACACGTATCAGTAACAATGCTTGTGTACATAATCAAA
CAGAAACATTAGATATACTGCA

BPB-7930
TGCAGCATGTTGGCATGGGCCGGCATACTAGCGTGCTGCCCACTTTTATTCAATTTGTTATTTTCTTGTGTTCATCCAGT
AGAAAAAAATCGCAGATTTTTTATTTGTTACGAAAATAAAAATATGAAATAAAAAAAGGTTTACGAATTTAAATAAAAAG
GTTCATCCATTTGAAAAAAATACTTAAAAGGTTCATCAATTTTAAAAAAAGTTCATCAATTTAAAAGAAAAAAATCATTG
AAACTGGAAAAAAGTCAACTGACGCTTAGATGGGCCAGCTCATTTACGAACACCACACGAGTTAACAAAAATGCACGATA
ACTGAATAGGAAATGCCACAGGACGGCCTGCA

BPB-6894
TGCAGTCCATGGGCGATAACCCCTCCACCGGCAGGTGCTCGCCCCGGTCGATCATCCTCCAAACCTCGTTCATCGCCACC
GTGGTCTGCGTCGTCGTGGGTACGTAGTTTGCATTAATTGTTTCAGCCAGTACATATATAGAACATTCGGTTTAAGTGAT
ATATATCAATCGAATTACACAGATAGAATATATAACATTCAGTTCAAGTGAATTATAATCACATGCATATACCGTGCGTA
CCGGATTGTTGGCGAACATGCCGGCGTCAATTAGGTTAAACTCCTCCAATTCGAAATTTAGTGGACACAAGTTTATAATG
TAGAACTTGTGGGCAGGGAAGAATGTTGGCGCGGCAGTTGCGGCGATGCAAATGTCCGAGAGGTTGGGGTTCATCACCCG
ATCCTGTTGCGCCTGTCAATGGTTCAGCAGCTCGATCAGTACATCCGATGACGATCAGTTGGTTCAGAAGCAGCGCAGTA
ATTAATAAGTAGCTAGCTTAGCTAGGGTACCTTAGAGGTGGAGAAGACCACAAGCTGATTGCGCTTGATGTCGAACGTGG
GCACGATGACGTTGGTGACGGTTTCCTTAAGCTTGCGGTCGCCGAGTTTCCCTCTGACCACCTTGCGGAGCCCCTCGCCG
TCGTACTTGGGGCGGAGCAGCGCGTACCTGACGAAATCCGGGTTCAGCAGCGTCATGCGCAGGCCCTCCACGAACTCCTT
GATGCACTCATCGAAAGGCGTCGAGTCCAGCGCCAGACCCTGCGCACGGCACATCGCCTGCA

BPB-7793
TGCAGTAAGAAAAGTTGCATCTTTCAGGCAACACAAACACACTGTCCACTTCTCTGTCAGTTCATTGCAATTGCGTTCTT
CAATCTTCAGTGATAACATCCCCTCTGAACAGAGATCATCATAATCTGCGTGTTACTCCCCTACGGGTTTTGGTTAAATC
ACTCTTCTAGTTTGACCTTTGGATAAATCTGCATATTCCTACTAGTTTGGCCTTTGGACAAGTCTGCATATTCCTATTAG
TCTGCGGGTAGTACTAAATTGTTTCTGCTTAAATCACTCTTCTATGGTGCTGGTTCTGTTGGTGCAAATCAGGAAGTGCT
TACTGGAGTATATCTAATGTTTCTCTTTGATTATGTATACAAGCATTGTTACTGATACGTGTTTTAGTTTCATGCGTGGA
ATTCAAATAACTTAGATTTGATTCTTTATAATTGCAGGGTTTGACATGGAATATGGGAGTGCCACCCTCGAGAACATTGT
AGAATATGACATGGTGATCAGTCAAATTTTTGGCAGCGAGGAAGAAGGTTACAATTACTATAATGCATATGCTCGGTCAA

AGGGTTTTGGTGTTAGGAAGGAAGAACCGACCAGGAAGTCGGGCACGAACATATCTTTTCGTCGCCTTTATGTCTATTGT
AAAGAAGGATATCGGGCGAGGAAGCACTTCAAAAAAACTGAACGAGTGTGAACTCCTAGGCCGCTGTCACGTTGTGNATG
TGGCGCCCGGATGGAGATCGAGCTTCGTATGGATAATGGTGAATGGTTTGTAAAANATTTTGTGGACNAACATAACCATC
CACTCGCTAAGCCTGACCAGACAGCTTTCATAANATCACATCGCGGACTGAGTGATGTGCAAAAGGNNNNNNNNNNNNNN
NNNNNNNNNNNNNNNNNNNNNNNNNNNNNNNNNNNNCAAGTACAAGAAGAAAAAAATGTTTAGCACAAGTGATGCATCTC
CTGGAAGTTTTTTTTGGTTGCATTGTGTGACAGAACATTTAGTATGCTTAACAAGATGACTTTTCAGAAAATATATATGC
AGTACANNATGAAACACAAGGAGTACTAGTTAATAAATNGCAACCTTTTTAAATAAGTTATGTNTATTACTTGTGATTAC
TGGACTAGTTGTTGCTCTACCATTTGATTGCATGAGCATGTGACAAATTTCATTGTACTTATAATCTACTTGCTTTTACT
GAATTTACATTGGCTCTAATATGCATTGTTCGTCCATAATGACTAATCAGATTCGATATATATAACATCGGTACAAGCGG
CACCGAATACCCAGCACCGTATGCCATTGTGTACCAAAACATGGCGATATTGGGTGTGGATGGCTTCAACACGCTTCCAG
CGAACTGCATGACTCTGTGCTACATCTTCTTCGCTGCA

BPB-3119

BPB-6199
TGCAGCGGTTCAGCAGCGCAAACTCTCGTTGTTCCTCTCGGTCTCTCCTGATCCACCCATCACAACAGCAACAGTATATG
CTTATATATATGGGATGTGTCGTGGTGTGTGCATGAGCAACTGAAATTTTCTAATAAGTTACATGGATAAGTTGAGTATG
TTGTAAAATGCATTAACCACTGAAAGATTGGATAAACATGTGCACCACATAAACNAGCATGCTATAAATTCCCATCCNAT
TACCTTTGAATGCTGAAGGAAGGAAAGCTTCAAAAGCTGAGTAATGTACATCTCGCCGAGCTCCCAAGCGGTGCATGTTG
GTGTTGCCCCAATAAAAGAAAGAGAAATCCACTGGTGAATTAGATCACTTTTAGATATCCTGTATCCTTTTGGAAAGATT
GCCCAATAGGCAAAGCAGAGCTTCAAATACCGAGGCATAANAGTATAGCTTAACTTCNAGGATGCAAGCACCCGTGTTGC
AGCTTTATCTTTGGGATCTGATGAAGAAGAAGATTCTTTCCNGATATCACTGTCTCTCANTTTCTTCCACTGATTATAGG
TCATAGTTTCCAAAATGGGTCCNNTTGATTGAGCAGCTAANCCACACCTCCNCACTTCGCTGCNNNNNNNNNNNNNNNNN
NNNNNNNNNNNNNNNNNNNNNNNNNNNNNNNNNNNCATTGCGTGTGGTTGCTATAACANCCACCTTGCCACCCCTTCCANCC
TTAAGCATGNCAGTCAGACTACGCAGTTGGGAATCATCCTCTGCCCACAAATCATCTAGAATAATAAGANCCTTCTTATT
GGCGAGTAGCTGTGCAAGTGATCTGTGTATCATCTGCGTTTCAGTGTAGGTACTCTCATTCTGTGACAGTTGACTTATTA
AAGAATTGCCAATTTTGTTCAAATCAAATGTCTCGGACACATAAATCCACACACGGACGTATTCTTCAAATTTTGTATTA
TTATTGTAAATCATTCTCGCCAAGGTCGTCTTGCCAAGGCCTCCAATGCCGTGCATAGCAAGGACAACGAGGTCTTGGAC
TGTGTTCTCAGATAAAGAAACCCATATTTTATTTTCCTCTTCGGTCCTCCCAATGATTGAATCCTCAATGGTTGAATATG
TTTCTCGCATTTGTATGAATTTCAGCTCATCAGCATTAGTGCCCGGCACTAACATGAATTCCCGATACTGATGTGTGATC
CCCTGCA

BPB-5173
TGCAGTGGTTGGTTTGGAGCAAGCACTTTGAGTGGCGCATATGGGCTTCGCTGGTCCCGATAGTCGGGGGGATCCTCCTG
ACCTCAATGACAGAGCTTAGTTTCAACATTTTTGGTTTCTGTGCTGCCATGATAGGCTGCCTGGCCACGTCTACCAAGAC
CATCTTGGCAGAGTCCCTGCTCCACGGATACAAATTTGACAGGTACATGCTTATCTCGTTGTGTAATATCTGATGCTTTA
GCGACTGGTGTTGTATCGCATTCTCAATTTTAAAGCATTTTATCTTCTTTCTTACTAAAATGATACTAGATCAAAAGATT
TAGTTCTTACTGTAGTGCTACCGTCAGGTTCAGTCCTGTAAGTTCTTGCCTCGGTCAGATGATTTATTAAGTATTCTCTG
TTTCTGCACTGGATCCATTCAAGGGCGAATTCTGCAGATATCCATCACACTGGCGGCAGCTCGAGCATGCATCTAGAGGG
CCCAATTCGCCCTATAGTGAGTCGTATTACAATTCACTGGCCGTCGTTTTACAACGTCGTGA

BPB-9683

BPB-9320

BPB-5363

BPB-2300

BPB-7445
TGCAGGTAGCACCATGTCCTCTGCATCAATCCATTTCGATCTGACTGTAATTCTGTTCCATTCCTTGCTTTGCATGTGTG
TGTGTGCGTGCGTGATCCTGCTGCATCTGTTTGCAGGTGGCCGTCAAGTGCAGGCCGCTGACCGACAACGAGAGCTGGCG
CTCGCGGCACATCATACAAGTCATTGACGATAAGGTAAACATTCAGAGCCTTGGCTTGATCCTGCTTGATTTGGCTGGTG
GTTTGTGTCATGGGTGGTTACAATTTTGCCATGGATGATAATGGACTAATGGTATGGTAGCAAGGTAGTAATAATGTTGT
GCTTGTATGTTTCTCCAGAATGTGGCCGTGTTGGACCCTGATGTCTCCAAGGACCGCCTTTTGTAATGATAACAGCTGTA
ATCAGCAATTGATTCAGCATCAGTTGGAAAAATAAGCAGGGATATTGTCATTAAAAAGATTACAGTTTGTGAAGCTTAGG
CGCTTTGCAACATCAGATTCGAGTTGATGTAGATATGCCATGCTTACTATTTGACATGAATACTTTGTGTTGTGCTTCTT
AGCTCTAATAGTACTTTGTGTTTACTTATGTAAATTAAAGGCCAACTTGTGAATTGGAAGGCTAGCTTGGATGGTGCTGA

TGAGTTCCTTGGATCATGATCGATGGCCAACTCCACCAGCCTCTGCA

BPB-7418
TGCAGAGCAGAGCGGGCAGTAGGGGTAGTAGCGATAGGACTTCCTCTGTCGCATCAGTAGAGTAGAGGCCTTGCTCTGGG
GAGCATCGCAGCGTCGTCTCTGTTGTAGGAGGAGAGAGGGGTACCCAAAAGGGAAACCCCCTCCTCTTTGTCAAGTCTCG
TAGAGTAGCGCAGTGGTTTGCATGTGATGTGCTAATCAGGCGGTCGTGGGTTCGAGACCCCACGGCGCCAACCCCCCTTT
TTCGTGTTTTGTTTCGCAGAGTAGCATAAGCAACAACAGAGACAATTTATCCTGTTAGCCATCCATGTTCTGTCGAGCAT
ATGGACAGTAGCAGAGTGGTGGCTTGTTCGTTGGTGAAACACGGGGTCTTGGGTTCGAATCCCTTCCCCATCCTTTTATT
TCTTTTGCTTTTCTTTTACTGTTTTGAATATGTTTGCTTGTATAGTTACTGCA

BPB-9870
TGCAGTGACTAGTGCGGATCACTCGCTGCTGCTGCCTGCTATGGGAGGTGCTTCTTAATTTTTATATAGCGGTGCCATTG
CGCGCGGCGTTGTGGTTGTGGCGAGCACCTCGTTGTGGGCGGACCACTGACAAGGTACAACGCGTAGGCGTAGCATTATA
TTTGTATGCTGTATATAATAACCTTTATATGCACAAGCAATGCATGCTCCGCGTGTTTGAAATCTGAGACCACTTTTTCA
ACAACCAACCATGGATCGCTAGCTTATTCCAAAGTTCCAGCTCAAAGGTCCTTGATCGATGGCCATTTTTTAATCCTCCG
TTCATACAGGCTTCTTTAATCACCACTAGTTCAACCGACAGGCTCCTCAATTTGTGCCATTTGCTTTTCCTCTTTGCACA
AACAATAGTAACGCTTTTTACTTGATTCTTTGCAGGTCTTTTTGTTGGGGGGGGGGGGNCCNNNNNNNNNNNNNNNNNNN
NNNNNNNNNNNNNNNNNNNNNNNNNNNNNNNNNTTGGGGNGGGGGGGGCCCTCCTTATCAGTTAACTAATTATTTTTGCCCC
ATTAATTTTCACCCGTCAAAAAATTGCCAGAGTCAATTACACCCGCAGGTCTGTGAACTTTACAAGAAAAATCAGTTTGA
TGCTAGAACTTACGGCATACATTGAACCGGTAATAAAACTTGACTCGGGCATGCATCTATGATTCTAATCATGTTTTGTA
TACATGTAACATACCGAAAAGGCACACCAACACGGCGTGGGACCCAATGTAAGTGACGGATGGCGGTGTCGTGCGTGTGG
CATGATTTTTTTGCAAAAAGAACACCTGATATTTTACCTGTTCACACAAAAAAGCATATCAATTGTTTTTCTTAAGTAAC
CTTGAAACATAACTTTGACAATTGGGCCCCACCCACCAGAACACAATACAACAAAGGAAAACGAGGACGCAAAATTAATC
ACTGCCAAGTTCAAACACACGACCTTAAACGTCAGTGCGGAAAGGCTAGCCACCAGCCGACCACACCATATATCACTTTG
TATAAATGGGAGCTCTATCCGTAATGACCTACAGACAAGAAGGCACATAGGCCTTAAATGGCCTGCA

BPB-0205
TGCAGAGGAGAAGCTTGCAAGGATGCTGGCAAAGAACAAGAAGGGAGACGACGAGTGGGGTTTAGGTAAAAGTAGAACTC
GTCTGGTCTCAGACGGTGCTAATATTGCTCTGGAGCTGCTCAAGGCTGATGGTTTGAAGATGCCGAAACTTTTGGAGCTC
GTCTTCAAGATTTGGGTGGATAAGCTGTTGTATGCAGCCACCCGATGCAACAGAGAATCCCATGCGAGGCAACTCGGCCG
TGGCGGTGACCTAATTACCATCGTATGGATTATGGTAGAGCATGTTGGCCCGTTTGAGATTGGCAAAGAAGGTCCAGATG
AAAAAGAGGAATCCAGTGATACAGAAGATCCGGGCGATGGAACTGGTGATTGGATCAATGACAATGAACACCCCTCATCA
GCGGAAGACTTAGAATCACAAGGGGGGGCTGGGGGTGGGGGCTGGCCACATCCACCATCATGGTTGCTACCGCCGGCAAT
GCCCCAACCTATGCAACCACAATGGTGCCCTCCCCAGCCGATGCATCCGCCATGTGATGGTGGACATATGTTCCCGTGGC
CGTGGCTTCAAGCGCCATGGGATCATCCGACGACATACTTGCCAACACTCGGAGACGGCGGACATATTATGCCATCGCGA
CCAGTTGCGGGGGCTCCACCCCCTTCCCCTCCCGGTGCCCCTCGTCCTGCA

BPB-6897
TGCAGGTTACAAAGTCTTGGAGGTCGGTGGCTCGGAGGAGGTGTACGACAAGATCATATTTGGTGCTCATGCACCTGATG
TTCTGAGAATACTAGGGGATGAAGCAACACACGAGGAGTTGAGAATTCTGGGTGCTTTCCAATATGTCCATAGGTATGAA
ATTTGTGTCTGCTATAAAATTTAATTTACTCAATTGATAAGTGCCGTACGTGTGGCACCAAACACTTTAGTCCTCAAGAC
AATGTTTTAAATAGCGGGCTATGACAAACTAGCGGCAGGCCTTAAAATCAGCTGTAGCAGGCTATAACGACATATTTGCA
TACGATACCATTTGACGGCACCCTGTTGAAATGGTTATAGCGGGGCTTCAGCCGGTTATTTAAAACTACGGAAAGGTGCC
ATAGCACCCGGGTCTCCCGACACCCGTTTATCTGGTGCGTCCAGTTTTTTTGAAGATTCGAGCTGAGGACTAGACAACAG
CAGGTCATTTGCGTCTGCATGTTAATTAAAAAGTTATTTTACATAGCAGAACATGGTCGGTCGTAGGCTGCACGAGACAG
AGTAGGTGTGACAGCACCGTGCTGCA

BPB-0715
TGCTCGAGCGGCCGCCAGTGTGATGGATATCTGCAGAATTCGCCCTTGATGGAGCCAGTGCAGGTTACAAAGTCTTGGAG
GTCGGTGGCTCGGAGGAGGTGTACGACAAGATCATATTTGGTGCTCATGCACCTGATGTTCTGAGAATACTAGGGGATGA
AGCAACACACGAGGAGTTGAGAATTCTGGGTGCTTTCCAATATGTCCATAGGTATGAAATTTGTGTCTGCTATAAAATTT
AATTTACTCAATTGATAAGTGCCGTACGTGTGGCACCAAACACTTTAATCCTCAAGACAATGTTTTAAATAGCGGGCTAT
GGCAAACTAGCGGCAGGCCTTAAAATCAGCTATAGCAGGCTATAACGGCATATTTGCATACGATATCATTTGACGACACC
CTGTTGAAATGGCTATAACGGGGCTTCAGCCGGTTATTTAAAACTACGGAAAGATGCCATAGCACCCGGGTCTCCCGACA
CCCGTTTATCTGGTGCGTCCAGTTTTTTTGAAGATTCGAGCTGAGGACTAGACAACAACAGGTCATTTGCGTCTGCATGT
TAATTAAAAAGTTATTTTACATAGCAGAACATGGTCGGTCGTAGGCTGCACGAGACAGAGTAGGTGTGACAGCACCGTGC
TGCA

BPB-2243
TGCAGGAGATCGAATAAAAATAAGAAAAAATGGCAACGAAGCGGCAGCCACCAATTGCGCACACGCGCACAAAGAGAGCA
AACAGGAGAGAGAACGTTGGATGGACGGTACCTCTCACACTTATACCACCCTCTACAGTTGGAAATGCAGACTATGGACT
AAGAATAGAACAAAAGTGAATCAAAAAATATTAGCTTAATCTCGATGAATATAGATCTTGATGCCTTTTGAAGGCCAACT
ACTAAGGTAGTAGTGCACACCATGGTTCCCATGGCTTTTTCAAAAGGAACGCAACACTATAATAGCGAAAACCTGATGTA
TATAAGCTACAGAAACAATATAGATAGAAGAACCAAGCATGTCCCTTTCATGAGAATACAGTAGGGTGGCTGTTTCTGCA
CATGAAAATAAAATCCACCAAAAAGGGAGAAAGAAGAGTAAAACCATAGCTGGTAACTTGGACACACATCANATATAGTA
CTGGTATAATTTAAAGCATAGTAAAGCTTGATAAATTAAAAATAGAACTTTGCAAACAACATGTAGATTGAAGTATCATT
TAAAATGAGTGAACTATTCATAGATACGTACTTTGTAGGTACAAATATCTTTCTCGAAAATACATTCCATCAAAG

BPB-9131
TGCAGTCCTGAAGGTAGAGGATACACGATCTTTACTATATTTTTATGGGTGTCTACATTGTTGTACACACTTTGATGGAA
TGTATTTTCGAGAAAGATATTTGTACCTACAAAGTACGTATCTATGAATAGTTCACTCATTTTAAATGATACTTCAATCT
ACATGTTGTTTGCAAAGTTCTATTTTTAATTTATCAAGCTTTACTATGCTTTAAATTATACCAGTACTATATTTGATGTG
TGTCCAAGTTACCAGCTATGGTTTTACTCTTCTTTCTCCTTTTTTGGTGGATTTTATTTTCATGTGCAGAAACAGCCACC
CTACTGTATTCTCATGAAAGGGACATGCTTGGTTCTTCTATCTATATTGTTTCTGTAGCTTATATACATCAGGTTTTCGC
TATTATAGTGTTGCGTTCCTTTTGAAAAAGCCATGGGAACCATGGTGTGCACTACTACCTTAGTAGTTGGCCTTCAAAAG
GCATCAAGATCTATATTCATCGAGATTAAGCTAATATTTTTTGATTCACTTTTGTTCTATTCTTAGTCCATAGACTGCAT
TTCCAACTGTAGAGGGTGGTATAAGTGTGAGAGGTACCGTCCATCCAACGTTCTCTCTCCTGTTTGCTCTCTTTGTGCGC
GTGTGCGCAATTGGTGGCTGCCGCTTCGTTGCCATTTTTTTCTTATTTTTATTCGATCTCCTGCA

BPB-7011
TGCAGAAAAATAAATAAATAAACAGCAATGGATGACAGTATGCTTAGGCGTGAACACCATGATCTGTGTGGCACCTAGCT
TCCTCACCTTCCCTAGCAAATTTTCTGATCAATTGGCACGCGATGGACGAACCCCCGGGTGTGTCCAACAAGGACTTGAA
CGTCGCGTTCAGCGCCGAACTGTCGGTCCGACAGGCTAGCGCCTTTGACGAAGTATCTTGTTCCTGCATCACACTGGTTC
TGTTTCTAGCATACATCTCTATCATAGAAACCATTGTTGAACTCTTCATGGTCACTCCACCATTATTCATCTCTTTCAGT
ATCTTATCCGCATCGGTGATCATCCCACTTTTGCAGCAAACTTCCATTACAGTCATGCAGAGGGCCTCATCAACCTGGAT
ATCTTCTTTTCTCATGTTCAGAATGAACGCACTTGCCTTCTCTAGCTGGCCTAGCTTGAAATACAATCTCAGTAGGTCAT
TGCAGCAGAACACATCAGGGAGGCCATATTTGCACAGAGCTCTGAAGGCCTCTTCAGCTGCA

BPB-9832

BPB-1967
TGCAGATGCCGGAGGAATATAATCTTTTCAGGAAAAATCTCTGTCAAATTTACATTATTTCTGAGTGTACGATCGAACAC
CATAACTCAACTAGGACCTGATGAGCAACTAACATCAGTAACAGCCTGTTCCTATGTTGCAGAAGGCGGATAATTCAAGA
ACAAAGAAAGAAAATAACACAATGCAGGAATCAGAAGGCAGGTAACTCTACATACAAGAAAGCAGATGGCACAACACATC
TGATAGAGAAAAAATGCATCTGACAAAGTTAGGAAAGTAGTTCTACAAGAGTAGCAGGAGGATTGAGAAAACTCTCTATG
CATTATTAAGCTGGTAAGCAAATGGCTAATTATGCAGAAAGAATATTATTTACAGCTAGCGTAGCTGCTTGAAAACCTCT
AGGGTATGCAGATAGCATATTTAATGTCGCAAGATCGGCACTATGACTATTCAAGAAACCTTGGTAAATTTAGCAAGAAC
ATTCACCTACTGTTGAAATCAGTGCCCGAAAGCATCAGAAGGGATAGGAGTAAAAGCAGACAAACTGTTATGATAGAGAA
TAAGAAGGGAATTGACCACACTTTGATTCAGCCATAGGCAGAGAATATAAGTACAGGTTGGAGCCACTCCTAGCCTAGTG
GAGATCATGGGTTGCCCACTACTCTCGGACGCGTGCANGGGTACAAACTGCACTGGATCCNTCAAAGGGCGAAATTCTGC
AGNATNTCCATCACACTGGCGGCCGCTCGAGCATGC

BPB-8033

BPB-23426
TGCAGGAGCATAGAAATAATGGGATGGAATCTCGCACAGGACCAAAGGTTGTTGTTTTTATTTAGCTGAAGTATTTGTTC
TTTCACAACTATTTTAATGTGTATAGAATAATTTAGCTTGTCCCCTTGTTTTGCAGGAACCACCGGAGAATGCTGTTATA
ACAACATGCTGCCATATTTTCTGCTATGAATGTGCACAAGAGAGCTTAAGTGAAGAGGAAGTCTGCCCTGTTTGCAAACA
GAAATTATGTTCTGAATTGCTTTTTTCACGCCCAGTACTAAGGCTCTGTATCTCTGATGAGTTGGAGTCATATGCAACAG
CAGCAGGAGATAGTTCTGCA

BPB-8050
TGCAGGAGCATAGAAATAATGGGATGGAATCTCGCACAGGACCAAAGGTTGTTGTTTTTATTTAGCTGAAGTATTTGTTC
TTTCACAACTATTTTAATGTGTATAGAATAATTTAGCTTGTCCCCTTGTTTTGCAGGAACCACCGGAGAATGCTGTTATA
ACAACATGCTGCCATATTTTCTGCTATGAATGTGCACAAGAGAGCTTAAGTGAAGAGGAAGTCTGCCCTGTTTGCAAACA
GAAATTATGTTCTGAATTGCTTTTTTCACGCCCAGTACTAAGGCTCTGTATCTCTGATGAGTTGGAGTCATATGCAACAG

CAGCAGGAGATAGTTCTGCA

BPB-8851

BPB-8721
CATGCGCTCCTTTGTTGCTCTTGTTGCAATAAAACAAAATCAATCTACTACTGTATTACGAGTAACTTATTATACGGCGG
CGCACAAGTAACCATGCGGTACTCCTTGCCAAACATTTGAGCAGTAGAAGTACCTTCATCGATCCGGATACGCAGTCTAC
GAAAGGGCGAATTCTGCAGATATCCATCNNNTGGCGGCCGCTCGAGCATGCATCTAGAGGGCCCAATTCGCCCTATAGTG
AGTCGTATTACAATTCACTGGCCGTCGTTTTACAACGTCGTGATNGGAAAANNNNGGCGAGTGAATTGTAATACGACTTA
CTATAGGGCGAATTGGGCCCTCNAGATGCATGCTCGAGCGGCCGCCAGTGTGATGGATATCTGCAGAATTCGCCCTTTCG
TAGACTGCGTATCCGGATCGATGAAGGTACTTCTACTGCTCAAATGTTTGGCAAGGAGTACCGCATGGTTACTTGTGCGC
CGCCGTATAATAAGTTACTCGTAATACAGTAGTAGATTGATTTTGTTTTATTGCAACAAGAGCAACAAAGGAGCGCATG

BPB-7874
TGCAGGACCACTGCCATTGGCGTTCTGCTCTCCGGCATCCGCCATGGCTCTGAAATTCCTTTNCAAATCTCTGNCTTTTC
TCTAAAGATTGTAGCCTATATGTGGGTNGGCAAGACTGAGATGATTGAGCGGATAACAATNNCACACAGGAAACAGNTAT
NCNANNCAAGGCCAAGCTNNCNNCGAGCTCGGATCCCTTAGTAACTGCCGCCAGTGTGCTGGAATTCGCCCTTGAGTAGT
GCCAGAACGGTCCATGTAGTTTTGTGGTGTAATTGCCTAGTTGGTCACTAAATCTGCACTGGATCCGGATACGCAGTCTA
CGAAGGGCGAATTCTGCAGATATCCATCACACTGGCGGCCGCTCGAGCATGCATCTAGAGGGCCCAATTCGCCCTATAGT
GAGTCGTATTACAATTCACTGGCCGTCGTTTTACAACGTCGTGACTGGGAAAACAATCAT

BPB-46599

BPB-6881

BPB-3056

BPB-6438

BPB-7991

BPB-3563
TGCAGGCGCACTTTAGGGGAACATTGTTCTGTGGAATGCAGGTGTACCTTCAAGTTGCCTTATGCGCAATATGTGCGTCA
ACCATGTTATATTATGTTCCCTTAGAGTTGGGTCACGCGCTCATTTGTTTTAGGGCATAAATACGTATGTGTGACTACCC
TAAACAGAACAAAAATGGAAAGGGGTCAAGGGACCTTTGCTTGGTTTGATTAATTCACGTGTTAAGATTGAAGTGTGATC
TTGCATTGATGTTCTACAAAGTGATTTGCTAGGAAAGAGTGAATTACAGAAAGATACAAGTTTAATATATGATATATCGC
TAAACTACAGATTGAAGAACAGATATTTACAACTATTGATTCAAGTGAACAACATGTAGTTTTGTGGTGTAATTGCCTAG
TTGGTCACTAAATCTGCA

BPB-6194
TGCAGATTTAGTGACCAACTAGGCAATTACACCACAAAACTACATGTTGTTCACTTGAATCAATAGTTGTAAATATCTGT
TCTTCAATCTGTAGTTTAGCGATATATCATATATTAAACTTGTATCTTTCTGTAATTCACTCTTTCCTAGCAAATCACTT
TGTAGAACATCAATGCAAGATCACACTTCAATCTTAACACGTGAATTAATCAAACCAAGCAAAGGTCCCTTGACCCCTTT
CCATTTTTGTTCTGTTTAGGGTAGTCACACATACGTATTTATGCCCTAAAACAAATGAGCGCGTGACCCAACTCTAAGGG
AACATAATATAACATGGTTGACACACATATTGCGCATAAGGCAACTTGAAGGTACACCTGCATTCCACAGAACAATGTTC
CCCTAAAGTGCGCCTGCA

BPB-4184
TGCAGTGCAAAACCACGGGTGTACAATGATCCTTTCAACAATCCGTTAGTCAAGATCGATGAAGGTACTTCTACTGCTCA
AATGTTTGGCAAGGAGTACCGCATGGCTCCTGTCCGGCTTACAAATGAACAACAGACAATGCATCAGAAAAGGCGATCAC
ATGCATATCAGTGGAAAAGGCCCACCGTGTTTCTTAAAGAAGGTGATCCCCTGCCACCAGATGTCGATCCAGAGACAGTT
AGATGGATTCCAGTAAACCACCCATTTGCTGCTGGTTCAGTTGAAGTAGATGAGGAGGCTGCCAAAAGGAATGTTTATCA
AAAGGATGGTGTCCCGTCCCGTGTTAAAGCCGAACATGAAGCTTTGAGGGCAAGGCTAGAGGCTTCTAATGATGTGAGTT
TTTGCAATAAACATGATACTCTGAACCGGCCAAATGCTACAATGCAGAAAGACTTGTGCTGCTACTTATTTTTAAATGTA
GTTCTATATATGCTGTTTGCAGACCTCTCTGCA

BPB-8949

TGCAGGCGGCTAATGGGAACCATCCCGAAAATCAGACTATAGACCAATCCAAGGTACGTAACAGATAAGTACTTCCCGAT
TCCCGATTTCTACAATTGCCCTTGCTTTTTTCAGGTCCCAAACAGGCCATTTGCGTATTCTGAACATTGATTGTTTCCAT
CAAGCGTGCAGCTCGTCTCCCTGTCTCGGAATTGTACACTCTTATAGCCTTGTCGAATTCCCTCCATCTCTGGACAGTTG
GCGCATAGATCTATGCACATAGATCTGGTAAGTAAATGCAAGATGGATTTTGCTATTTTTACTTATTTTTGTTGTTTCAT
ATGTTGTAGGTTGCCATTGTACGTTCTGTATATATATATCTTTTCCACTTTGGATATAGGTAATTCCAGCCCAAACAAAT
TCATGACACCTTTTCTATGTGAATTTATAGTTACCTATTTATTTTATTTGTCTGACAAAAATTCTACCAATTCTCTTAAT
TTAAATCACTGCTCTTGAGTGCATAATTTTTGTTACCAGTATAAATGATCAGCACTCCAAATAGTGTGTTTCTAGCAGAA
TAATACGCAGAAGTGATTTTTTCCCCGCCTGTGCAACATTATCCTTGTTAGAGCTTATCTTGTTTTCTTTTTGGCATAGC
ACATGACTAGTTCTTGTTTCCATATATTAGATGGCGCCTCATGCACAGATAACTTTATTGATAACCTCTAACTAGTCCAC
TACTATGATTTTAGTTTTTCTTTTTCTTCTTTCTATCCCTAGCCATATGATGTATTCACCATTTGTTTTGGCATGATCTG
CA

BPB-3131

BPB-5755
TGCAGCAGCCATGGAAGTCCATGCTGTTACACGGAACAGCAACGTCGGGATTTATTGCCATATAGCCGTGCAGTTTGAAT
AGTAGTACAGTATGAAGCTGTGCATTATTTGTGGGCAAGCGGTCCGGCTTTTCTGATAATTGGTCAGCTTTCTCTTGACG
GAGGTTTGGTTTCCTGTAATCAATGCCCTTAATTCGGCAAGCTGCAACCAATCCGATCTCATGAGTTTAGAGTATATACT
TCTGCCAACAAGATCGGAGAGAGTTCCTTTTGCAGGCTGTCAGTTAGGCTGTCGAGCACTGGCAGCTGTTCCATTGTGCT
GGAAATAAGTTCGGGTTATTTTCTGCA

BPB-4228
TGCAGTACAGCAGTTGCTAGTAACGAATCAAACAGTGTTTCAAGAACCTAAAACACTGCCTCCTCATAGAGTATTGGATC
ACTCTATTCACTTCGTACTTGATGGTATGCCTGTCAATTGCAGACCATATAGATACTCTCATGTGTAGAAGGATGAAATA
GAGAGACAGGTGGCAGAAATGTTGAAATCTGGTCTGATTACACCCAGTGTGAGTCCTTTTGCTTCTCCTGTTTTACTGGT
CAATAAAAAAGATGGAACATAGAGGTTTTGTGTGGACTACAGAAGAATAAATGCTTTAACCATTAAAAAAATCCTATGCC
AGAGATTGATGACTTGCTTGATGAATTACGGGACACTAAGTGGTTTTCCGAACTGGTCCTTAGAGCTGGATATCATCAAA
TCAAAATGAGGGAAGAAGATGAATTTAAGACTGCTTTTAAAACACACCATGGACACCTTCAGTTCAGGGTTGTTCCATTT
GGTCTGACCACAACCCGAAACACTTTTCAATGTGTGATGAATTTTGTGTTTGCAGGGCCAAACAGAAAATATGTGTTGGT
TTTCATGGATGACACATTGGTTTTCAATCAAACATTTGAGGAACATTTAGAACACCTGCA

BPB-4093

CHR. 3H

BPB-8547
NNGTGTGCTGGAATTCGCCCTTGATGGATCCNNTGCAGGCGTGAGGATTGCCTCATACACGACAAGGAAGAAGATCGTGT
CTGGTATGGCGAGGAGGTGAAGCGCATTGATGGTGAGGAGCTGAAGCTCATACTCCATGCAGGTTCGGAGAGCAGCACAT
GACGCCAACCACTGCGATTGCTTTCTGGCCCATTGGAGCTACCAATGTTCATGTCAGTTGCTAACGACACCATCAGCGGT
CAGTGGGTTCACCCTCCATAGTTGGTTCGACTCTTTCACACCACTCATATTGGAATGCGCCAAGAATATTTGTTTCACTT
CAACATCTCATAAAGTACTTTTGGGAGTATTAAACTACTGTCCGAATGTATTGCTACTAACTAGTCCCGGACGGACTGCA
TAGATATAGCTGATTAATTTACTACACTTGCAGCTGCA

BPB-0634
TGCAGTGGAGTGGGACATTGCTGCTCGCCACGAGCCAAGTATAGCGGTGCTGCAATGGAGCGGGCGCTGGTTCCGACGGT
GCGACCAGGGCGCTTCAATGGCATGAGCGTTGCCGTCGTCGGCACGACAAGTCAAGAGCGACAGTGCTGCTGCCGGTAGC
GTTGTAATGGAACGAGCGATGGCGCTGTTGCCATTGGATCATTGGTGCTGCAATGGAGTATTTGCGCATGGTGGTGCCTG
CGCGACGATGAAGCTTCAATGCATGGCTGGTGTTGCTATGGAGCATCGCCGGTGTTGCTATGGAGCATTCCGGAGAATCA
CCGGTGTTGCTATGTAGCATCCTCGGCCGTCGGGGCATCATCAATGCTGGAATGAGGCATCCCGGAGAGTTGCCGGTGTT
GCGCTAGAGTGTTGTCGGGCCGCCGGAGCATCACTGGTGCTTCATGGGATGATCGCAAGCCTGCA

BPB-6797
TGCAGTAGCTGCTAATTTTTTTTGATGGTGGAGGGGCACGTCCATCTTTGATGGTTGCCTGTGATTTTAGGTTCTAAGTG
ATATCTGATTTGACATAGTACTGATCCTAGTTCCCCCTATGTGTTTTTATATAGGCGTTCTGCAAAGAGTATAGACAGGT
TGCTCCTCCTGTTCAGTCGAGGTGTCTTTTTGGATCCAGAGATGGCTGGCAAGTAGACAGACAAGGAGCGCGAATGACCT
TTTTAAATTTTGTCGTCATTACTTGTATCAGTTCGTTTTTTTTTTTTTTTGCT

BPB-6884
TGCAGAATGGTGCCGTAACTTTTTGATGCAGAGGCCGGTGGCTGTGTTCCTCCTTTAAAAAAAAGGGTGTCATAACTTTT

TATTTGCTCCCTACATTCCCTATTAGTTGTTTCGCTATGACCAAGGAGGAGCTTCGCGCGAGGGAGGTGTACGGGTAGTT
CAATAACTTCCCAGTGATGGGATAAGCACCCGTGGATGTGTAATTAACGAGTGGGGCAGAAATCAGCAACTCGGGTGCAA
AATGATAAATGGGACTTAGAATTCGAAAATTTGCTAAATACAATACCTTAAAGAATGGAATGGAAGCAGTAACGTTAAGT
GAAACTTTAGCGACAAACGCAAAGGTGAATTCAGGAGAAGTCGCGAGCAAATGAAATCAAATGCATGAATGGAGAGGTGG
CTAACCTTTGATTTTCNNGCCTTTTCTTGCATACGCTTTGACAAAAGTTTTATGATAACTTATCCGACTGAAAAAGTATT
TAATTTTCTTATGAATTCTCGACAACTCATCTTCCGGACCACTCACTGTGGGTCTTTTCCTTCTTCTTTTCTTAGCAGTT
GGTTTCACTAGAGTGTCCACTGAGTTGCTAGGTGGCTTCTTCGCGTCTCTCGAACGAAGAATCCTGACCTGAGTCTTCTG
GATAACGTTNTTGGTCTGAGTAACGTCCTTAACAGAGGCAGAATTATTTCTATTGTNNNTCGGCGGCGAGAAAATATATT
ATTGTAGGAAAAC

BPB-2018
TGCAGAGTTACCGAGATCTATTACAGGCACCGTGTCATCAACATCATCCCACTTGTGGAACCATCAACTCAGTGCCCATT
TCAGAAGCTCATCTCAGCAAACTTAGCAACTGATGTGTACAAACAACCTCAATCATCACAACAAGTTATGACCATGGTAC
GTTGTTCAAGAGATTTCACACCAGTAGATCAATACCGTATAGTAGGCCCAGCCTCTTGTGTCAGTAACAACGCTAGCCAA
TTCTGGTATATGGCGGTATCTTATGCATACATATCTGATCTTCCACGAGACTGCATGGCTGTTTCTAAAGGCATCCCCGT
ACCCTTCACTTATGATAATCATGGCCCCAACTATGATTACACCTTNNAGGCAAATACAGTCCTCAACTCTGGAGAGACAA

BPB-44917
TGCAGCTGTACTGGTACTCGGAAGAACAGATACGGCAGGAGGTCAAATTCTTAGCTTGTAGTTTTATGCTGGCCTTCCAA
GTCATTTGAATGTTATTTCATTTTTTGCGAAAGCATCTGAATGTTATTTATATGGCTAAAATTAACGCAGATTAAATGGG
AATGCCGTAGCAATTTGTTCTCGGTCTACAAGTCGTTTTGACGTTTACATGTCTGGAATTATTGCTGTAGAAATTCGGAT
GACAGATACTGAACTTTTCTTGCCCTGAGAGCGACGTTGCTGCTGCTGTCCAGTTATTTATAGTGGTTCATATGAGGCAG
AGGACTCGTCGTCCTTGCAGTGCTTGTCAATCACACTCTTAATTGCAGGAACGCATACGGCAGGAGGTTGCATTCTTGGC
TTGTACAGTAGTTTTCTGCTGAACTTCAAAGTCATTTGGATGATTTTTATACGGTTGAAACTGCCGACGATTAAATCGGA
ATGCGTGTGACAGCAAAAAATCAGCTTAATCTTTCTCACCCTGAGCTGACGGTGAGGTTGCTGCTCCTGTCCACTAGTTT
GGTCCATATGCAACAATGGACTGCA

BPB-7235
TGCAGCTATTCATCAATGCAATCAAAACCCATTTACATATGACCTAGCTGTCCACCATAAAGGCTTCAGGAATCCTCCGG
TGGGAAAGTTGCCTAAGAGCATGGTTAATAGTATAGCCAACTGCTGGCTATAAGCAGTCTTATAGTTCATCTTATAGCTA
GTTTGTATAATAGTTAGCTATAAAAAAAATACAACATTTGTCATATATGGCCCACCTTTCATTCTCACAAAACACCTAGG
AACACGTGCTAGAGCTGGCTCTTCACGAAGAGTCCGCTTCCCTTCTCTCTCCTCTTCTCTCACATTCAACTCAGCAAAAA
TATAGTATTTTAATTCTTATAACCTGCTGACTGTACCTTACTGTACTTGCTCTAAAAAAAACGTCTCGTTTCACGAGGAG
TCCATCAGATTGGGTCGTGCCTCTCTTCTTGTTACTGCTCCTGACTCGCACGCGTCATCCTCACCCTTGAAGCCCCATGT
GGCGTCTCTGCA

BPB-42312
TGCAGTCCGTTGTGTTATAATAGGGCTAAGCTGGCTCTGTTATGGAGGAGATGCTTTCTCTACCACAGAAACCTTTTATA
CTCATAGGCCTCTTGGAGGCTCTAGCAGCAGCGTAAGGGATGGCTGCTGGGGGTGATTTTGTTTCTTCTAAAAAGCAAAT
ACAACATGTTCCTCATATATCTTCACTTGTACAATGTACTTGATATGTGACATAAGGAACTCAAAATCAAACTATTATAT
GCTTACTTTTGCTGGGGCAGCTATTCTTTTTGGGACTTCAATACCAATATTATCACATGTTGGTGGGTCATCAGTATAAT
CATATGACAGTTTTCACTTTGTCACCTCACCTGCAATCACATTTATATAATTATCTTACTACCTTGCTGCA

BPB-1114
TGCAGCAAGGTAGTAAGATAATTATATAAATGTGATTGCAGGTGAGGTGACAAAGTGAAAACTGTCATATGATTATACTG
ATGACCCACCAACATGTGATAATATTGGTATTGAAGTCCCAAAAAGAATAGCTGCCCCAGCAAAAGTAAGCATATAATAG
TTTGATTTTGAGTTCCTTATGTCACATATCAAGTACATTGTACAAGTGAAGATATATGAGGAACATGTTGTATTTGCTTT
TTAGAAGAAACAAAATCACCCCCAGCAGCCATCCCTTACGCTGCTGCTAGAGCCTCCAAGAGGCCTATGAGTATAAAAGG
TTTCTGTGGTAGAGAAAGCATCTCCTCCATAACAGAGCCAGCTTAGCCCTATTATAACACAACGGACTGCA

BPB-2744
TGCAGAAGTTACAATTGGGACAATGGTGCTGTGGAGACGACCATGACCACTACGTGCGTGGCAAACGGGGAAGAGGAGCA
TGGACCTCCACTACGTGCATACACCGGTGGCGTGCCCCGGGATTTAGACGGGAGGGAGAGGGGCAGAACATGGCCGGGTC
ATGGTCTTGCACCAAGCCCACACAGCGCCCACACTTACTTACATGGAAGCTTCATAACTTAATAATGTAATTAATACTTG
ACGGATCATTGTTTCTGGATTTTTCTTTCAGCGTACGTACCAGTAAAATAAATTGTAACTTAGAACAGTACTGCACTACC
TTTTTCATGAGTATACTAGATATTGCTTCCTTCGTTTAAAAGTATTTGTCATAGAAATGAATGTATCTAGATGTATTTTA
GTTTTAATTATTTTTATTTCTATACATTTGCGTGACAGGTAATTCCGGACGGAGGGGGTATTTTTTTAGCAAAGTATAGT

AAGTGTTGTTTGTTTAACTGAAGGTCGTTTTTGCGATTTGTCCTCTTTGTAACCGACTGCA

BPB-7965
TGCAGCCACAGCGTTTCCAAGGCCCTCGGTACAGACCCCAGCAGCTCATCAGCCCCAAGGCCACGTTCATGTCACCAAGA
ACGAGCTTGTCTGTGGCGTCGTCGGGTTCCTCGCGGCATGTTTCCTCCTCTCTCTGCTGATCGTCATCGCAAAGAAGGCG
AGGAATGGGGCGGCGCCGGCGACCGATCCGGAGCCGCGAGACGATGAAGATACCGTGCTGAAGGTTGCTGAGCGGTGCCT
CGGCTGCTTCTTTAAACTTCTGTTATGCGCGTGATGGGCTCGGTCGATTGTTAGCAGTGCAATTTACCTTGTTGTTGGAT
TGTATCATTGTACGCACTGCTACTGTAGCAGTGGTCTGGTACACTGCCCCTTTTATTCTTTCACCTTGTGTTTGTAGTAT
CGCTTTATAATCCTTGAGCTTACACCTCGGAGAAATCTGTAACTGTGGTTGTTGTTGAATAAATCTTACTGCACTGGCAA
TACTACTAATATTGTTTCTATATTAATCGTATTTGCAGCTTGTGCTTTTCTTGGTGTAAACAGTCGTGCACTGTCCTACT
GTTTGCATCGCTTTTATCTCTATATAGCCTATGCTAATTTCCACGTTATCTTCCTAAAAATTACTCGTATCTGGGATTCT
ATTACACTGTGGAGATCCAGACTCCTTAACCATGTTCACCAACCCTGATCAAATCCTGAATTTGTCTGTCCTTCATCACT
ACAATAATCTTATTGTTTTTGGATGTAAAAGCAACTCTTAACTTTTTCTTAATTAATAAATGAGGTAAATCTTTTGCCTC
CGTTTTTTTTAAAGCTGTTATTGTGCGCTGCTAAGCCTTAGCACCGGGCGCCCGGTGCTGCTCTGTGTTTCTGACCGGTG
GCCGGTGCTAGCG

BPB-1264
TTGGGCCCTCTAGATGCATGCTCGAGCGGCCNCCAGTGTGATGGATATCTGCAGAANTCGCCCTTGATGGNNCCAGTGCA
GTCCATTGTTGCATATGGACCAAACTAGTGGACAGGAGCAGCAACCTCACCGTCAGCTCAGGGTAAGAAAGATTAAGCTG
ATTTTTTGCTGTCACACGCATTCCGATTTAATCGTCGGCAGTTTCAACCGTATAAAAATCATCCAAATGACTTTGAAGTT
CAGCAGAAAACTACTGTACAAGCCAAGAATGCAACCTCCTGCCGTATGCGTTCCTGCAATTAAGAGTGTGATTGACAAGC
ACTGCAAGGACGACGAGTCCTCTGCCTCATATGAACCACTATAAATAACTGGACAGCAGCAGCAACGTCGCTCTCAGGGC
AAGAAAAGTCCAGTATCTGTCATCCTCGAATTTCTACAGCAATAATTCCAGACATGTAAATGTCAAAACGACTTGTAGAC
CGAGAACAAATTGCTACGGCATTCCCATTTAATCTGCGTTAATTTTAGCCATATAAATAACATTCAGATGCTTTCGCAAA
AAATGAAATAACATTCAAATGACTTGGAAGGCCAGCATAAAACTACAAGCTAAGAATTTGACCTCCTGCCGTATGTGTTC
TTCCGAGTACCAGTACAGCTGCA

BPB-44182
TGCAGGAAATACTGATACAGAGATAAATCACCAGAAAATCAGAATAAGGTAACCAACAGATTGCTGTATTTTTAACTATT
ATTACAATCCAAGTAAAGTTTAATGTGATTCTGATTACAAATATCTAGTACTAGTATTACATGGCGCCGGATCATTATTC
ATACATCAGATGATCATGTAGTGGAAAGACCGAAATATATTGCATGCTAGTGATATGCATTGATGTGCAACAGATGGACG
GAGCAGTACATGCCCCCAGTATTTACTAAATTGTGGCAGCATGTTTTCCATGATTTACACCACAGGGTAATTTTCAGATG
TGATGAATGGCTTTGGGGGCATCTGTAGATTCTGCAACCTCGCGGTTAACATGTTTACCACCTTTGTCATTGATGGCTGA
TTCTTCGGGTTCCACTGGATACACCATAGTCCCACAATGGCAAGTTGTCTGACCTTTTCTTGCTCTTCCTGTGCCCAGTC
CAAAGTAACTACTAAGTCCTGCCCACTGCTTACTTTCTGATAGATCCACTCCGGGAGATACACATCGTTCTGGCTACCAA
CACTTGGGTCCGAGTTCCTCCTTCTACTCACCATTTCTAACACTAGCATGCCAAAACTGTATACATCAGATTTGTATGAT
ACTCCCCCAAAGTTCCGTGAGTATAGCTCCAGGGCAATGTAGCCCATCGTGCCTCTTGCTGCA

BPB-3689
TGCAGAGCTTTTCACAGAGTGGAAGGTACATGTAATTAAGGGTCGGCTGGCAAAGGGCACGGTATACCGACCCGACATAG
AAGAGCCCGAAGAGGGTAGGTGGCATGCCACAACACTCGCTGACCAAGCTAATGTTTCCTTGGCGCCAAGTGTTCGTTTG
GATCAGGATGTTCTAATTGCCATAGTGGGCCGCTAAGACAACCAAAAGCATCGGATATGGGGAAATCCCGAAAATGGACA
GGAATTCAGCGTGCGATGAGTTTTGATCGAGCCACGTGTCAAGCCAGAAGAGGGTACCGACAACATCACGTATGAAGAAA
GTGATACCCAAGTGAATCCCATTGTTGATCGACTGGGGAAATTTCTAGAACCGATACCCCTCCCACGGATCACATGCCAG
AAGAGGGAGACCCTGTGGGTATTTGGCTTTTATAAGCTGCA

BPB-7624

BPB-3025

BPB-2891

BPB-1799

BPB-2531

BPB-5977

BPB-8515

TGCAGTACAACGGTAGGGACGCTGACGGTAAGATTGATGAAGAATTCGAAGATAAACCGATAAATAATTTTTATATAGAA
CAAAGCTAATCTAGGGTTAATATGTACTCCCTCCATGACAAAAATATAAGAGCAGTTAGATCACTAAAGTAGGGATCTAG
ACGCTCTTATATTTTTGTACACGGGGAGTAGTAGAATCCTACGACCAGTCAAGGACTATGTATCGTATTACCTTATTTAT
TCATTCTATCCGTGTCACTAAATTATTCTGTTTTTGGTAGATGCAACATCGTTAAATTTGTGAACAGAAAGTAGATGGGT
GTTGGGCCTTCTGCTCTTTTTGGCACTAGAAAATGTAATATTAAATATTTGTTTGCTGTGCTTGGATATCTATTGCTCTT
GTCATCCTTCTTGTAAATTTTTGTATTAATGCCCTTATATATATAAAGGACACACCCTCTCCGAAAATGGCGTTTGGTGG
CCGAGCACCCTGCA

BPB-3649
TGCAGAAACGAAACGATTAACTGGGTCACAATGCTCATAACTTTTAACATAAATAAGATGGCGAAAACAGAATATACTAC
ATAACAAGAAGATAGGTTAGTGCTACACATTTCATTATTTTATCAGCTACCATAGGTATTACCCCTACAGAAGAAACTCT
CTTGGCACGTAGAACTACATTCAATGATAGTTCATCAAAGTTTTTCACTTGCTCAAGTTTAAAGACAAGTCAATATGAAT
ATGTACATATAATAATTTGATAAAAAAAAAGTGTACATATACTTAGTGCATTTACCTGATAGATCTCAGTCGTCAAGTGT
TCGAAACAGATGGTGCAGTCAAGGGTCTCCTTCAATATTCTGACATCCATCCNAGTGTCTCCCTCTCTGATAGCTTCAGC
ACTTCTTTTGTTGGAGCTGCTGCCGTTCATCGCCNAAAAAACTCAAGGGTACCAGAACTAANTTGCTGGCTGTGTGCTGC
TGGTACTACNGACCGAAACCAAGATCCNAACCTCTTGATCCACACAACCTCACCGCTCCNTTCTCCGCCCTGTACCACAA
GAGCACATGANATACTGATAAAATTC

BPB-8441
GATCCAGTGCAGCGCAAACATTTGCGTTAGATTTACCAAAAAATCATTGGAAAAAGTGCTAGAAAATCCAAAAAATTGAG
TGCAACGCTAAAGAAATCATGCGGATACGCAGTCTACGAAAGGGCGAATTCCAGCACACTGGCGGCCGTTACTAGTGGAT
CCGAGCTCGGTACCAAGCTTGGCGTAATCATGGTCATAGCTGTTTCCTGTGTGAAATTGTTATCCGCTCAA

BPB-0945
TGCAGCGAGTGACCATCCCAACCGCCCGGATCTTCGTATCCGATACATCGAAAAAGGTACACAACTGCACCTCCACGCAT
CAAATCATGCGCTTCCTCCCATTCCTGTGATCCACCACGTGGAAAATTCAATGCTCATTCCTCTGATCCTCTCTCTATTT
CTCAATTTAGGCAAAAAGAACTTGCAGGCGCTGAGGTTTGCTTCTTGTGCATGGGATTCCTCTGTTCTACACGCATTGTT
TTGTTTGCCGCCGATTGTTCAGATTTTCTTTTCATCAAGCAAGTGTTTAGCATTCAACTTCCTTTTTCCTTTCAATTTCA
GATCAAGGGCTCGAGTTATCGAAATTATCCTGCA

BPB-1852

BPB-6322
CATGGATGTAGAAGCACTACAAATCCATGGCACACGGCGACAACCATACAGGATTGATGTCACTGCCAAGAAGCTGCACT
GGATCCGGATACGCAGTCTACGAAGGGCGAATTCCAGCACACTGGCGGCAGTTACTAGTGGATCCGAGCTCGGTACCAAG
CTTGGCGTAATCATGGTCATAGCTGTTTCCTGTGTGAAATTG

BPB-6127
TGCAGAAACGAAACGATTAACTGGGTCACAATGCTCATAACTTTTAACATAAATAAGATGGCGAAAACAGAATATACTAC
ATAACAAGAAGATAGGTTAGTGCTACACATTTCATTATTTTATCAGCTACCATAGGTATTACCCCTACAGAAGAAACTCT
CTTGGCACGTAGAACTACATTCAATGATAGTTCATCAAAGTTCTTCACTTGCTCAAGTTTAAAGACAAGTCAATATGAAT
ATGTACATATAATAATTTGATAAAAAAAAAGTGTACATATACTTAGTGCATTTACCTGATAGATCTCAGTCGTCAAGTGT
TCGAAACAGATGGTGCAGTCAAGGGTCTCCTTCAATATTCTGACATCCATCCAAGTGTCTCCCTCTCTGATAGCTTCAGC
ACTTCTTTTGTTGGAGCTGCTGCCGTTCATCGCCAAAAAAACTCAAGGGTACCAGAACTAAATTGCTGGCTGTGTGCTGC
TGGTACTACAGACCGAAACCAAGATCCAAACCTCTTGATCCACACAACCTCACCGCTCCTTTCTCCGCCCTGTACCACAA
GAGCACATGATATACTGATAAAATTCATGGATTTGGAAGTGACACTGCACTG

BPB-7199
CTTCCAAATCCATGAATTTTATCAGTATATCATGTGCTCTTGTGGTACAGGGCGGGGAAAGGGGCGGTGAGGTTGTGTGG
ATCAGGAGGTTTGGATCTTGGTTTCGGTCTGTAGTACCAGCAGCACACAGCCAGCAATTTAGTTCTGGTACCCTTGAGTT
TTTTTGGCGATGAACGGCAGCAGCTCCAACAAAAGAAGTGCTGAAGCTATCAGAGAGGGAGACACTTGGATGGATGTCAG
AATATTGAAGGAGACCCTTGACTGCACCATCTGTTTCGAACACTTGACGACTGAGATCTATCAGGTAAATGCACTAAGTA
TATGTACACTTTTTTTTTATCAAATTATTATATGTACATATTCATATTGACTTGTCTTTAAACTTGAGCAAGTGAAAAAC
TTTGATGAACTATCATTGAATGTAGTTCTACGTGCCAAGAGAGTTTCTTCTGTAGGGGTAATACCTATGGTAGCTGATAA
AATAATGAAATGTGTAGCACTAACCTATCTTCTTGTTATGTAGTATATTCTGTTTTCGCCATCTTATTTATGTTAAAAGT
TATGAGCATTGTGACCCAGTTAATCGTTTCGTTTCTGCACTGGATCCNTCAAGGGCNAATTCTGCANATATCCNTCACAN
CTGGCGGCNGCTCGAGCATGCATCTNGAGGGCCCAATTCGCCCTATAGTGA

BPB-7448

BPB-6978

BPB-6468
TGCAGCACGACACATGTTGTCAAACCATCTCTTCTTTAGTTTTCTGTAACAAGACTCATGTTGCACAACCTTGTTCCATA
ATTTTCTACGACAACGGTGTTGTTGCAAAATTTACAATAACATCTCCATTAAATCTAATTTTGTGTAACAAGATCCATGT
TACAAAAATTACAACATTATTTTCAGCTGCCCACGCAGTTGCGTAGTCGTCGTTCGGTGTCATCATTCTGTAACAACATC
GTTGTTACAAAGATAAGATGAGAGAGAGATATGTGGTCCAATAGGACACATGTCAAGCAACGCAGGCGGTGGATGCCACA
TTTTTGATCGGCCGGTTGATCATAATCATTTCTCATATTTTTATTGATCTGATGTATGACCAGCATAAAACCATTTTCCT
GAAAATGTTCATCAATATCGTTGCGTGATGGTCTAGCATACACCATCATGTGCCCATTCTATAACAGGCAATAAATTGGT
CATCGTCTGTCCTCTGTGCGCCGTCCATGCATGATGATGTACCATTTTAATCCTTTTATGGACCTACTGCTGCGCCATAC
GCGAGAACGAACTAGAAGTTGAGAATTAGTGGTCCTAGTATGGATGCTCATCAGTCATCAGCGGCACGATCGACCATATT
CCAGCAGCTCATGAGTCATGAGAAGCTCCTGTCCTACTCCCGAGCCAATATATAGTTGAATGCATACGCATGACATCTCT
CGACTCTAGTTGATTCGCCCTCTGTGAATCATCACGGTCGGTAAAGTTCACCTGAATTTCACGGGACCAGCGTGTCCGCT
ACGGCCGGTGTCATCACTCACGGCGAGTCCGGAGCTGGCGAGGAACCTAACTGTCGCAATCTTCCACCGACCTGATCGAT
CAGTCAGTCACACCGGTTCTTCTGTGGTTCAGATGCGCCTTCGCCTCTGCA

BPB-48285
TGCAGCTCGTGTCCACAGGAAGTAAGGAAACCACTAGGATGCGTCTCCAGCACCTCACCTGCCCGATCCCTCTCGTATGC
ATTGCGCTCGGATGACCAGCATCACCAAATCAGTGGCTGAGCTATTGGTGGGTGGCGCGCTATGCTCGTATCTCCGGTGG
TTCACAGAGGAGGACGTCAACTCCCAAATTTCTTCTCTGCATAAGCAGGTGCGCTTTAGTTTCGCGCCTTGGGGACACGA
CAGCGCAATGTTCTTGGTAACAGTGCCTTGCGATCCTCCCGTTATGATTGACAGCATGAGCAAAGAGTAGTACGTTGTGA
TGCCCGCATAATAACTGAATGCCTTTTTTTTAGACAAATAACTGCATGCTTAAAAACCCTCATATGCCACCACCACTCTT
GAAACCAGTGGCGGATGCAGGCACAATTTCCTGGTGTGGCGGAGGTAACTAGCATATGTGTCTCAGCTCAAATCATCAAT
GAAGAATCATGTAGACCTAGCATACATGACACGAGTTAGGTATGGCAGCAGCCAAAGCTTGCCAGATTGCTGGCTTCGCC
ACTGCTTGAAACTCCCAATGTGGAAGTTGTGCTTATAACACCATTTATAGTTTTTAGTTGAAGTTTGGGGATAGGGGATG
CAATGATTGGTAATCCACCACTGACTGGACTGAGTCCTTAAAACTGCA

BPB-7350
TGCAGTTACCAAGGTTGGGCCTGAATTTGTTGGTTGTAAGAAGGGTGATCCTGTATGTAATGAGTTGGTTGCTTTTCCCA
AACTTGAACGGTTGATTTTCATTGATATGCCCAACTGGGAGGCGTGGTCCTTTTTTGAGGAAGAAGTTGTTGCTGCTGAT
GCAAGGGGAGAGGATGGAGCCGCTGAGATTCAAAATGAGGATGCCCAATCTCCAAGGTTGCAACTGCTGCCTCGTTTGGT
ACTGTTGACACTCGATGGTTGCCCGAAGCTGAGGGCTCTCCCGCGACAACTTGGAAAGGACACCACCCGCCTGAAGGAGC
TCAAATTAATTGGTACAAACAGCTTGAAGGCCGTGGAGGACCTCCCGTTGCTCTCTGAACTCCTTAACATTCAGAAATGT
GAAGGCCTGCA

BPB-8172
TGCAGTCGCAAATCTTACCATGACTTCGAGGCATATGCTTTCAGCAATCTGGAGGTACTCATCCCACAAATCTATAGTCA
TGACATAGTCAAGCATCCTTACAGGAGGCGTGCACTTCCCATAACTAGAGAACACAATGTTTCCAATAACATCCCGCTTG
ATGCAATCAACGTAGGCCCTGACACCTTGATATATGCGTTAGAACACATGTTCTCGCATCTAAACGACGGTGGAAATAGG
GTTCAAACAACACATCAGGGTAAAAGTAGTCGTCGTACAACATAATACGGCCATGTGTCATCTGTCGGTTGAACCAGTGT
AGTTGGGAACATGCTCCGCCACACGTTCCACATCTGCA

BPB-1814
TGCAGGGAAGGAACAGAGAAGCAGCCGCCAAACTAAGTACGCCACGGGCTTCTGAAGGTGAAGAGGAAGGTGAAGGTATG
AGGAAGTATCGATCACCCCTTCTTGCAATTGTTACTTATAGAACACAATATCCATGTATGCAGTTCATCATGACTGGAGT
GTGTGCTTTGGTGTCTCTGAGATTTACTACTTACTTATTTGGGTGGCATGCGAATGAAATGTTTAAATGCAGCCGAGCAT
GTTTCTGGGGACAACCACATACAGATTGGAAGCGGGCTGCTTGAGATCCAGAACAAAGAAGGTCAAGGTATAAAGAAGTA
TTGATCACACAAACCTTGTTGCAATTGCTACTTATAGAACACATTTTCCATGTATGCACTTTCATCATGAGTAGAATGTG
TGCCTCATTATCTGTGATTTATTTGTTACATTTCGAGCAATGTGCAAGTGAAATGTTCAAATGCAGACAAGTATGTTTCT
GCGGACAAGTATGTTCAGATTGGAGGCTGGGAGCAAGGAAGGAAGAGGAAAGCAACTGTGAGTGTGACACCGAACGGAGT
GCAACCAATTGGAATTGAAATCCAGAACAAAACAAGTCACGGTATAGAAAAAATAATCACTAGCTCTTAGTACCCGCACA
TTTCAAAATACACAATGAGTTTCTTGATAATCTTTTGTATTATTGCAGGTGAGTGTCTGAGAGCTGAGAATCAGATGGTG
AGGCTGCACTGGNTCCATCAAGGGCGAATTCCAGCACACTGG

BPB-2548
TTTTCCCAGTCACGACGTTGTAAAACGACGGCCAGTGAATTGTAATACGACTCACTATAGGGCGAATTGGGCCCTCTAGA

TGCATGCTCGAGCGGCCGCCAGTGTGATGGATATCTGCAGAATTCGCCCTTGATGGATNCAGTGCAGGGAAGGAACAGAG
AAGCAGCCGCCAAACTAAGTACGCCATGGGCATCTGAAGGTGAAGGTATGAGGAAGTATCGATCACCCCTTCTTGCAATT
GTTACTTATAGAACACAGTATCCATGTATGCAGTTCATCATGGCTAGAGTGTGTGCTTTGGTGTCTCTGAGATTTACTAC
TTAGTTATTTGGGTGGCATGCGAATGAAATGTTTAAATGCAGCCGAGCATGTTTCTGGGGACAACCACATACAGATTGGA
AGCGGGCTGCTTGAGATCCAGAACAAAGAAGGTCAAGGTATAAAGAAGTATTGATCACACAAACCTTGTTGCAATTGCTA
CTTATAGAACACATTTTCCATGTATGCACTTTCATCATGAGTAGAATGTGTACCTCATTATCTATGATTTATTTATTACA
TTTCGAGTAATGTGCAAGTGAAATGTTCAAATGCAGACAAGTATGTTTCTGCGGACAACTATGTTCAGATTGGAGGCTGG
GAGCAAGGAAGGAAGAGGAAAGCAACTGTGAGTGTGACACCGAACGGAGTGCAACCAATTGGAATTGAAATCCAGAACAA
AACAAGTCACGGTATAGAAAAAATAATCACTAGCTCTTAGTACCCGCACATTTCAAAATACACAATGCGTTTCTTGATAA
TCTTTTGTATTATTGCAGGTGAGTGTCTGAGAGCTGAGAATCAGATGGTGAGGCTGCA

BPB-2965
TGCAGGGAAGGAACAGAGAAGCAGCCGCCAAACTAAGTACGCCACGGGCTTCTGAAGGTGAAGAGGAAGGTGAAGGTATG
AGGAAGTATCGATCACCCCTTCTTGCAATTGTTACTTATAGAACACAATATCCATGTATGCAGTTCATCATGACTGGAGT
GTGTGCTTTGGTGTCTCTGAGATTTACTACTTACTTATTTGGGTGGCATGCGAATGAAATGTTTAAATGCAGCCGAGCAT
GTTTCTGGGGACAACCACATACAGATTGGAAGCGGGCTGCTTGAGATCCAGAACAAAGAAGGTCAAGGTATAAAGAAGTA
TTGATCACACAAACCTTGTTGCAATTGCTACTTATAGAACACATTTTCCATGTATGCACTTTCATCATGAGTAGAATGTG
TGCCTCATTATCTGTGATTTATTTGTTACATTTCGAGTAATGTGCAAGTGAAATGTTCAAATGCAGACAAGTATGTTTCT
GCGGACAAGTATGTTCAGATTGGAGGCTGGGAGCAAGGAAGGAAGAGGAAAGCAACTGTGAGTGTGACACCGAACGGAGT
GCGACCAATTGGAATTGAAATCCAGAACAAAACAAGTCACGGTATAGAAAAAATAATCACTAGCTCTTAGTACCCGCACA
TTTCAAAATACACAATGAGTTTCTTGATAATCTTTTGTATTATTGCAGGTGAGTGTCTGAGAGCTGAGAATCAGATGGTG
AGGCTGCA

BPB-5487
TGCAGCCTCACCATCTGATTCTCAGCTCTCAGACACTCACCTGCAATAATACAAAAGATTATCAAGAAACGCATTGTGTA
TTTTGAAATGTGCGGGTACTAAGAGCTAGTGATTATTTTTTCTATACCGTGACTTGTTTTGTTCTGGATTTCAATTCCAA
TTGGTTGCACTCCGTTCGGTGTCACACTCACAGTTGCTTTCCTCTTCCTTCCTTGCTCCCAGCCTCCAATCTGAACATAG
TTGTCCGCAGAAACATACTTGTCTGCATTTGAACATTTCACTTGCACATTACTCGAAATGTAATAAATAAATCACAGATA
ATGAGGCACACATTCTACTCATGATGAAAGTGCATACATGGAAAATGTGTTCTATAAGTAGCAATTGCAACAAGGTTTGT
GTGATCAATACTTCTTTATACCTTGACCTTCTTTGTTCTGGATCTCAAGCAGCCCGCTTCCAATCTGTATGTGGTTGTCC
CCAGAAACATGCTCGGCTGCATTTAAACATTTCATTCGCATGCCACCCAAATAAGTAAGTAGTAAATCTCAGAGACACCA
AAGCACACACTCCAGTCATGATGAACTGCATACGTGGATATTGTGTTCTATAAGTAACAATTGCAAGAAGGGGTGATCGA
TACTTCCTCATACCTTCACCTTCCTCTTCACCTTCAGATGCCCATGGCGTACTTAGTTTGGCGGCTGCTTCTCTGTTCCT
TCCCTGCA

BPB-6825
TGCAGGGAAGGAACAGAGAAGCAGCCGCCAAACTAAGTACGCCATGGGCATCTGAAGGTGAAGGTATGAGGAAGTATCGA
TCACCCCTTCTTGCAATTGTTACTTATAGAACACAGTATCCATGTATGCAGTTCATCATGACTAGAGTGTGTGCTTTGGT
GTCTCTGAGATTTACTACTTAGTTATTTGGGTGGCATGCGAATGAAATGTTTAAATGCAGCCGAGCATGTTTCTGGGGAC
AACCACATACAGATTGGAAGCGGGCTGCTTGAGATCCAGAACAAAGAAGGTCAAGGTATAAAGAAGTATTGATCACACAA
ACCTTGTTGCAATTGCTACTTATAGAACACATTTTCCATGTATGCACTTTCATCATGAGTAGAATGTGTACCTCATTATC
TATGATTTATTTATTACATTTCGAGTAATGTGCAAGTGAAATGTTCAAATGCAGACAAGTATGTTTCTGCGGACAACTAT
GTTCAGATTGGAGGCTGGGAGCAAGGAAGGAAGAGGTAAGCAACTGTGAGTGTGACACCGAACGGAGTGCAACCAATTGG
AATTGAAATCCAGAACAAAACAAGTCACGGTATAGAAAAAATAATCACTAGCTCTTAGTACCCGCACATTTCAAAATACA
CAATGCGTTTCTTGATAATCTTTTGTATTATTGCAGGTGAGTGTCTGAGAGCTGAGAATCAGATGGTGAGGCTGCA

BPB-0527

BPB-7273

BPB-0158
TGCAGCTGGAGGATGGTCACCTGCTCCTCCGACGGCTACGTCTCCGCGCTGTACGTCCTTCTCCCCGCCCATCCTTGCTC
ACGCCGGTCTCTCTCTCCGTCGAGGAGCGTCCGTCGTCGGTGTCCTCGTCTTATTTCCGGACTAATCTTCTCATGTGATG
GTCTGTGCTCACAGCGGTCTGCCCAGCCAAAGGCTCTCCGGTAAACTGTCGCCCGGCATCGGGAACCTCACGAGGCTGCA
ATCTGTGTAAGCTCGTCGCTCAGATTTATCAGTACAGTTTCTTTGAAAAATGTATTGGACAGATGAGAGATTGCAAAGTG
TTCAACTTCAACATCTCTCCTGTTCTTGGACAGGCTACTGCA

BPB-9746
TGCAGTTTTAGCCAAATTAAGTTTAACCACTCAGTTCTGCTTGCAGTTCATACGCATGTACAGTTGCAAGTGCTATCAAA
CTTGGATCTGGGGGTAGAGAGAGGGGAAACTCACTTGCACAAGAGGAGGGGAGTAGGAAGTAGCACTGGAAAGGCTCATC
TGTCCTGACCACGTGAGGAGGACGCAGCCATCAGTCAGCTCGTCCATACACGTGCTCATCAGGATCACCCTCCTCTCCCA
GCATGGCCTAGAACCCGACCCTCAGTATCTAGCTTGTCCACTGGGACGATTCAACAAAATGATTTTTCTACTATAATAAC
GATTGTTGTAGCCATATTTTTTTTACTTTATATAAACTTTGCCCCTTTCATGAACAAACTTGCCCCCATATGCTTGCATG
CTGGCTTCGTTCCTGCTTGTCCATGCAAGAAGACTTGCACCAACTGTATTACAAAGTAATGATAATAAATATGCATATTT
TGGCACGAGTATTTGCTAACTAATGAACTCACATCTTTACTCTACTGATGACTATTCAAAAGTGTGCATCTCCATCTACT
GTATGAAAGCCTTATTTTAATCTGTTGGAGGTTGAGACCGACTAACTCCGTGCCTGCCTACCAACCAAGCTTCAACCGAT
CATTATAGTCAGTCAACTCTGATAATTTTGAGTCATCCGAAAGACCGAAATTGAACAAAAACGTGATTATTTTACATGCA
ATGGGATGCACAAACAGAGACACACCCAGTTGACTCGGGCCAATTTTAAGTCATCCGAAATTGAACAAAAACGTGTTGCT
CACACGCACAGTTTGCTGCTTCAGTTTCAGTCATGGCATCCACAATTCTTATGCATCCAAAACTGAAGGTACAAATAGCA
GGCAGGTGATATTCAGATCTACCTGCTGCACCTACAGAGGCGAGATGAGGTCCAAGAACCCGCAACAGCTTCTCCATTGC
CTTCCTTGACACAACAGCAGGCACTCACGCATCAGAGAAAGAAGAAAGCCAGAGGATGAAGAAAACCAGAGGAAGAAAAG
GTGAGGAGAGATCCATACCGAGGCTGGCTCTTCTTGGAAGGGATGCTGACCTGCGGCCCCATGGGTGGGGAGCAGTGGGG
CTAGGGTTCCGGGCGGTGGTTGAGGTTGGAGGGGGTGGAAGGTGGAGGAGGAGGAGAACTCCTCTGCCGATGCGTCGTGG
TGGAGAAGGAAGGAGCTCCAGCCGAGCCGGCGGTGGCCTTCTGCTGGTGGCTGCA

BPB-6347
TGCAGTTTTAGCCAAATTAAGTTTAACCACTCAGTTCTGCTTGCAGTTCATACGCATGTACAGTTGCAAGTGCTATCAAA
CTTGGATCTGGGGGTAGAGAGAGGGGAAACTCACTTGCACAAGAGGAGGGGAGTAGGAAGTAGCACTGGAAAGGCTCATC
TGTCCTGACCACGTGAGGAGGACGCAGCCATCAGTCAGCTCGTCCATACACGTGCTCATCAGGATCACCCTCCTCTCCCA
GCATGGCCTAGAACCCGACCCTCAGTATCTAGCTTGTCCACTGGGACGATTCAACAAAATGATTTTTCTACTATAATAAC
GATTGTTGTAGCCATATTTTTTTTACTTTATATAAACTTTGCCCCTTTCATGAACAAACTTGCCCCCATATGCTTGCATG
CTGGCTTCGTTCCTGCTTGTCCATGCAAGAAGACTTGCACCAACTGTATTACAAAGTAATGATAATAAATATGCATATTT
TGGCACGAGTATTTGCTAACTAATGAACTCACATCTTTACTCTACTGATGACTATTCAAAAGTGTGCATCTCCATCTACT
GTATGAAAGCCTTATTTTAATCTGTTGGAGGTTGAGACCGACTAACTCCGTGCCTGCCTACCAACCAAGCTTCAACCGAT
CATTATAGTCAGTCAACTCTGATAATTTTGAGTCATCCGAAAGACCGAAATTGAACAAAAACGTGATTATTTTACATGCA
ATGGATGCACAAACAGAGACACACCCAGTTGACTCGGGCCAATTTTAAGTCATCCGAAATTGAACAAAAACGTGTTGCTC
ACACGCACAGTTTGCTGCTTCAGTTTCAGTCATGGCATCCACAATTCTTATGCATCCAAAACTGAAGGTACAAATAGCAG
GCAGGTGATATTCAGATCTACCTGCTGCACCTACAGAGGCGAGATGAGGTCCAAGAACCCGCAACAGCTTCTCCATTGCC
TTCCTTGACACAACAGCAGGCACTCACGCATCAGAGAAAGAAGAAAGCCAGAGGATGAAGAAAACCAGAGGAAGAAAAGG
TGAGGAGAGATCCATACCGAGGCTGGCTCTTCTTGGAAGGGATGCTGACCTGCGGCCCCATGGGTGGGGAGCAGTGGGGC
TAGGGTTCCGGGCGGTGGTTGAGGTTGGAGGGGGTGGAAGGTGGAGGAGGAGGAGAACTCCTCTGCCGATGCGTCGTGGT
GGAGAAGGAAGGAGCTCCAGCCGAGCCGGCGGTGGCCTTCTGCTGGTGGCTGCA

BPB-6329
TGCAGGGGCCGGCAGGGCCGGGCAAGAAACAACATATGTGAACTGGGGATAAGCGCTGGAGAGACCCCACGGCCACCATT
GGCTGGCCGACGACTCCCTCAAGGGGGGAGTAACAAGATAATGAGGTTTTGTTGGCGAGGAGAGCGACATGGGAGGCGTA
TGATCGGAATCAAAACAAATAGTCACATGTGTGCTGTTATAATTACGAGAGTTGATCCTGAATACAGAAAATAATTTATT
GTGACATTCAGTACGAACATTTGATATACATTGTTGCTAAAGATCTGTGATCTCTGCA

BPB-1710

BPB-6914
CATGATTTGTTTTGTTCTGGATTTCAATTCCAATTGGTTGCACTCCGTTCGGTGTCACACTCACAGTTGCTTTCCTCTTC
CTTTCTTGCTCCCAGCCTCCAATCTGAACATAGTTGTCCGCAAAAACATACTTGTCTGCATTTGAACATTTTACTTGCAC
ATTACTTGAAATGTAATAAATAAATCACAGATAATGAGGCACACATTCTACTCATAATGAAACTGCATACATGCGGATAC
GCAGTCTACGAAAGGGCGAATTCCAGCACACTGGCGGCCGTTACTAGTGGATCCGAGCTCGGTACCAAGCTTGGCGTAAT
CATGGTCATAGCTGTTTCCTGTGTGAAATTGTTNTCCGCTCA

BPB-3317
TGCAGATATACGTGGACGGTTGCGCTGTGTGCTGCCTGCGAGTCCCACATCGGCTGGCTGTTCAGGGCTGACAAAAGGAA
CCTTCTTCCGATATCCTTCTGGGGGATCCGCATTTCCCAAACCTCAGACGGTACACAATCGGCTCAGGACCGACGTTCCG
TGTAAGGCAGCGGCCGCGGTTCTTGGCGCGATTCTCGTCATCCTTCAGCAGACCCGTGACCTATCTTCGGTGGCCTGCTC
GTTGTAGACCTTGCTGGATTTGGCCCGACGGTTTCCTGCTCGTTGTAGACCATGCTGGATTTGGCCTGACGGTTTCATCT
AGTATTCCCACTGCA

BPB-9130

AGTGTGATGGATATCTGCAGAATTCGCCCTTGACCCAGTGCAGTATGTAGGTGCAGAACTGATTGCATGAATGGACCGCA
AAGAGATAAAACATGCCGCAAATTTGTTCTGAAGCACAAGAGTAAAACAAATTGCACTTCGAAATTGCGTTGTTCCGTTC
AACAAAAGGAAAATATGCATGGAACCATCAAAAGAAACTCAACAGGTGCCGCCGCAGTGAACAGCAAAAGCACTTATTTA
TCCAACATACTCCTACACAACCTTGGCTCAAATTGCGCACATCTATCATCAAAGATGCACAGGAAAGATAACAACGAATA
GTGGTTATATTCATGGAAGAAATTCTGATATGAATCAAGTTTTCTGTATCTATGCAGATATGAATGAAAGAATTCCAGAT
ATCAATCTGTTTGGAGGTCGACAAAAATTCTGACACCAAAGATCTCCATCCAATCTACTCTAAGATTATCTGTCTGTTGT
ATCATCCGCTGGCTCTCTTTGGCATGGATGTGCCTGAGCTTCACTCCAGACTCCAGAGTCTCGGGTGATAGTGTAGCTCC
TCTGCGCTACATCATGGTGATGTCTGTGAAATGAAACAGCAAATTAAATCCAATTAGTGACAATATCACAGCCATTAACT
TGATTAAGATTCGAAGGATGCAGGTACGGATAACAAGAACAATCAAAGGCTGCA

BPB-2646
TGCAGCCACCAGCAGAAGGCCACCGCCGGCTCGGCTGGAGCTCCTTCCTTCTCCACCACGACGCATCGGCAGAGGAGTTC
TCCTCCTCCTCCACCTTCCACCCCCTCCAACCTCAACCACCGCCCGGAACCCTAGCCCCACTGCTCCCCACCCATGGGGC
CGCAGGTCAGCATCCCTTCCAAGAAGAGCCAGCCTCGGTATGGATCTCTCCTCACCTTTTCTTCCTCTGGTTTTCTTCAT
CCTCTGGCTTTCTTCTTTCTCTGATGCGTGAGTGCCTGCTGTTGTGTCAAGGAAGGCAATGGAGAAGCTGTTGCGGGTTC
TTGGACCTCATCTCGCCTCTGTAGATGCAGCAGGTAGATCTGAATATCACCTGCCTGCTATTTGTACCTTCAGTTTTGGA
TGCATAAGAATTGTGGATGCCATGACTGAAACTGAAGCAGCAAACTGTGCGTGTGAGCAACACGTTTTTGTTCAATTTCG
GATGACTTAAAATTGGCCCGAGTCAACTGGGTGTGTCTCTGTTTGTGCATCCATTGCATGTAAAATAATCACGTTTTTGT
TCAATTTCGGTCTTTCGGATGACTCAAAATTATCAGAGTTGACTGACTATAATGATCGGTTGAAGCTTGGTTGGTAGGCA
GGCACGGAGTTAGTCGGTCTCAACCTCCAACAGATTAAAATAAGGCTTTCATACAGTAGATGGAGATGCACACTTTTGAA
TAGTCATCAGTAGAGTAAAGATGTGAGTTCATTAGTTAGCAAATACTCGTGCCAAAATATGCATATTTATTATCATTACT
TTGTAATACAGTTGGTGCAAGTCTTCTTGCATGGACAAGCAGGAACGAAGCCAGCATGCAAGCATATGGGGGCAAGTTTG
TTCATGAAAGGGGCAAAGTTTATATAAAGTAAAAAAAATATGGCTACAACAATCGTTATTATAGTAGAAAAATCATTTTG
TTGAATCGTCCCAGTGGACAAGCTAGATACTGAGGGTCGGGTTCTAGGCCATGCTGGGAGAGGAGGGTGATCCTGATGAG
CACGTGTATGGACGAGCTGACTGATGGCTGCGTCCTCCTCACGTGGTCAGGACAGATGAGCCTTTCCAGTGCTACTTCCT
ACTCCCCTCCTCTTGTGCAAGTGAGTTTCCCCTCTCTCTACCCCCAGATCCAAGTTTGATAGCACTTGCAACTGTACATG
CGTATGAACTGCAAGCAGAACTGAGTGGTTAAACTTAATTTGGCTAAAACTGCA

BPB-3113

BPB-3278
TGCAGAGGCGAAGGCGCATCTGAACCACAAAAGAACCGGTGTGACTGACTGATCGATCAGGTCGGTGGAAGATTGCGACA
GTTAGGTTCCTCGCCAGCTCCGGACTCGCCGTGAGTGATGACACCGGCCGTAGCGGACACGCTGGTCCCGTGAAATTCAG
GTGAACTTTACCGACCGTGATGATTCACAGAGGGCGAATCAACTAGAGTCGAGAGATGTCATGCGTATGCATTCAACTAT
ATATTGGCTCGGGAGTAGGACAGGAGCTTCTCATGACTCATGAGCTGCTGGAATATGGTCGATCGTGCCGCTGATGACTG
ATGAGCATCCATACTAGGACCACTAATTCTCAACTTCTAGTTCGTTCTCGCGTATGGCGCAGCAGTAGGTCTATAAAAGG
ATTAAAATGGTACATCATCATGCATGGTCGGCGCACAGAGGACAGACGATGACCAATTTATTGCCTGTTATAGAATGGGC
ACATGATGGTGTATGCTAGACCATCACGCAACGATATTGATGAACATTTTCAGGAAAATGGTTTTATGCTGGTCATACAT
CAGATCAATAAAAATATGAGAAATGATTATGATCAACCGGCCGATCAAAAAAGTGGCATCCACCGCCTGCGTTGCTTGAC
ATGTGTCCTATTGGACCACATATCTCTCTCTCATCTTATCTTTGTAACAACGATGTTGTTACAGAATGATGACACCGAAC
GACGACTACGCAAGTGCGTGGGCAGCTGAAAATAATGTTGTAATTTTTGTAACATGGATTTTGTTACACAAAATTAGATT
TAATGGAGATGTTATTGTAAATTTTGCAACAACACCGTTGTCGTAGAAAATTATGGAACAAGGTTGTGCAACATGAGTCT
TGTTACAGAAAACTAAAGAAGAGGTGGTTTGACAACATGTGTCGTGCTGCA

BPB-2406
TGCAGAGGCGAAGGCGCATCTGAACCACAAAAGAACCGGTGTGACTGACTGATCGATCAGGTCGGTGGAAGATTGCGACA
GTTAGGTTCCTCGCCAGCTCCGGACTCGCCGTGAGTGATGACACCGGCCGTAGCGGACACGCTGGTCCCGTGAAATTCAG
GTGAACTTTACCGACCGTGATGATTCACAGAGGGCGAATCAACTAGAGTCGAGAGATGTCATGCGTATGCATTCAACTAT
ATATTGGCTCGGGAGTAGGACAGGAGCTTCTCATGACTCATGAGCTGCTGGAATATGGTCGATCGTGCCGCTGATGACTG
ATGAGCATCCATACTAGGACCACTAATTCTCAACTTCTAGTTCGTTCTCGCGTATGGCGCAGCAGTAGGTCTATAAAAGG
ATTAAAATGGTACATCATCATGCATGGTCGGCGCACAGAGGACAGACGATGACCAATTTATTGCCTGTTATAGAATGGGC
ACATGATGGTGTATGCTAGACCATCACGCAACGATATTGATGAACATTTTCAGGAAAATGGTTTTATGCTGGTCATACAT
CAGATCAATAAAAATATGAGAAATGATTATGATCAACCGGCCGATCAAAAAAGTGGCATCCACCGCCTGCGTTGCTTGAC
ATGTGTCCTATTGGACCACATATCTCTCTCTCATCTTATCTTTGTAACAACGATGTTGTTACAGAATGATGACACCGAAC
GACGACTACGCAAGTGCGTGGGCAGCTGAAAATAATGTTGTAATTTTTGTAACATGGATTTTGTTACACAAAATTAGATT
TAATGGAGATGTTATTGTAAATTTTGCAACAACACCGTTGTCGTAGAAAATTATGGAACAAGGTTGTGCAACATGAGTCT
TGTTACAGAAAACTAAAGAAGAGGTGGTTTGACAACATGTGTCGTGCTGCA

BPB-2630
TGCAGAGGCGAAGGCGCATCTGAACCACAGAAGAACCGGTGTGACTGACTGATCGATCAGGTCGGTGGAAGATTGCGACA
GTTAGGTTCCTCGCCAGCTCCGGACTCGCCGTGAGTGATGACACCGGCCGTAGCGGACACGCTGGTCCCGTGAAATTCAG
GTGAACTTTACCGACCGTGATGATTCACAGAGGGCGAATCAACTAGAGTCGAGAGATGTCATGCGTATGCATTCAACTAT
ATATTGGCTCGGGAGTAGGACAGGAGCTTCTCATGACTCATGAGCTGCTGGAATATGGTCGATCGTGCCGCTGATGACTG
ATGAGCATCCATACTAGGACCACTAATTCTCAACTTCTAGTTCGTTCTCGCGTATGGCGCAGCAGTAGGTCCATAAAAGG
ATTAAAATGGTACATCACCATGCATGGACGGCGCACAGAGGACAGACGATGACCAATTTATTGCCTGTTATAGAATGGGC
ACATGATGGTGTATGCTAGACCATCACGCAACGATATTGATGAACATTTTCAGGAAAATGGTTTTATGCTGGTCATACAT
CAGATCAATAAAAATATGAGAAATGATTATGATCAACCGGCCGATCAAAAATGTGGCATCCACCGCCTGCGTTGCTTGAC
ATGTGTCCTATTGGACCACATATCTCTCTCTCATCTTATCTTTGTAACAACGATGTTGTTACAGAATGATGACACCGAAC
GACGACTACGCAACTGCGTGGGCAGCTGAAAATAATGTTGTAATTTTTGTAACATGGATCTTGTTACACAAAATTAGATT
TAATGGAGATGTTATTGTAAATTTTGCAACAACACCGTTGTCGTAGAAAATTATGGAACAAGGTTGTGCAACATGAGTCT
TGTTACAGAAAACTAAAGAAGAGATGGTTTGACAACATGTGTCGTGCTGCA

BPB-9336
TGCAGATGGCGATGTGTATTCCTTTGGAGGCAACCAGTTCGGGCAGCTGGGGATTGGTTCTGATCAAGCTGAGGTGGGAA
CTTTGAGTCCTTTTTCCATTCTTATTACATAACATAATCCGATTCAGGGATACTAAGTGTGTAGCTGTCTCTTCACTTGC
CCAAATACATAAGAATGTTACTTCTGTTTTGATGCAGACTATACCAAAACTGGTGGATGCCCCCAGCTTGGAAAATAAGA
ATGCAAGATCAGTGTCGTGTGGAGCTCGTCATAGTGCAATAATAACAGGTATAGCGGTCGTATTTTCTTCTGGAAGTAAT
GTTTTATTTTATTTCTTGATTCTAACATTTTTTTACGAAATTTCTGCA

BPB-18973
TGCAGATTCAGTGGCTGTTGCGAGGGAATGAGCTCGCAGTGGTGATGGCGCTCGGTGTATTTTGTGAAAAGGAGATGGAA
GTGTGTGGGTGGGTGTGAGCTAAAAGTGACGGCGAGTCAGCAAGTGTGGCCGAGAATCCAGAGCAATTGATTGGCGTCGT
GTCACGGTCAGTACTGTACTTAGCTAGTAATGCGGGGTTGGACGGGAATTTAATAAGCACACGCTTAAGTTTGCCGGTCA
AAGAGGGTTTGATGAATCCGGATGCAGAGATGGCTGCGGAAGCATCGTGAACCAGATTAATTCCCCATCTGTGTGGACTG
ATGCTGCA

BPB-0619
TGCAGATTCAGTGGCTGTTGCGAGGGAATGAGCTCGCAGTGGTGATGGCGCTCGGTGTATTTTGTGAAAAGGAGATGGAA
GTGTGTGGGTGGGTGTGAGCTAAAAGTGACGGCGAGTCAGCAAGTGTGGCCGAGAATCCAGAGCAATTGATTGGCGTCGT
GTCACGGTCAGTACTGTACTTAGCTAGTAATGCGGGGTTGGACGGGAATTTAATAAGCACACGCTTAAGTTTGCCGGTCA
AAGAGGGTTTGATGAATCCGGATGCAGAGATGGCTGCGGAAGCATCGTGAACCAGATTAATTCCCCATCTGTGTGGACTG
ATGCTGCA

BPB-2137

BPB-5396

BPB-5298

BPB-4156
TGCAGTTACACAATGTCGACAAGTATGCTCCGCTGCACCTTGTGTTCTGGAACTGATTGTGCAACCGTGCCGGGCAGTTT
CTTCTGATGGATGCGACTTCTCAGGTTTGGTTGGGGATAGGATAGAGATTACCCATGTTTCGTTCGTACAGCTCCTATTG
TTTTTGTGTCGAGTCGCGGGATGGTCATTTATTTGTATTAGCTTTGACGCACTTCTTCTGCTTGTATCTTATTAGTGCGA
ACATGGTTTAGCATGCAGCAAAAGTATCAGGTTATTCAGAATTTCAGAGCCCATATATTATTGTTCTCATTTTGTTCCTA
GAAGTGGAAACCATGTTGTTCATGTATATACTGCTATTTTTGTCAGTCATGCACCCATCTTGCAAATCAGCTCAAAACAT
TTTCAAACCGATCTCATTTGATTGTGGGAGTGACAGACAATGAATATCATGGAAGGAAACCTCAGTCAGGGGTGACAGGT
GAGCAAAGAAAACAGAAGTACTCCCTCCGTCCTACAATATAAAAGCATTTTTGACAGTATAGTAGTGTCAAAAACGCTCT
TATATTATGGGATGGATGGACTGTTTTGTACTGGAGAATGCTTCACATTACCAAACGAACATTTACAGAGCATCTTCCAG
TCCAGCGTGGTGGTGTTATCCCATCTCTGAATGCAGAAACAATGAAGCAGAGAGGGGAGTGACAGGCTGCGTGCGTGGTG
CTACTATGATAATACATAACTTCTTTACATGGGTTTCACTGGGCGCTGTGTCTTCTTCGCTCGTCCGACAGACCGGGCTG
CTGTTGCTGCA

BPB-2420
TGCAGTACAACGGTAGGGACGCTGACGGTAAGATTGATGAAGAATTCGAAGATAAACCGATAAATAATTTTTATATAGAA
CAAAGCTAATCTAGGGTTAATATGTACTCCCTCCATGACAAAAATATAAGAGCAGTTAGATCACTAAAGTAGGGATCTAG
ACGCTCTTATATTTTTGTACACGGGGAGTAGTAGAATCCTACGACCAGTCAAGGACTATGTATCGTATTACCTTATTTAT

TCATTCTATCCGTGTCACTAAATTATTCTGTTTTTGGTAGATGCAACATCGTTAAATTTGTGAACAGAAAGTAGATGGGT
GTTGGGCCTTCTGCTCTTTTTGGCACTAGAAAATGTAATATTAAATATTTGTTTGCTGTGCTTGGATATCTATTGCTCTT
GTC

BPB-8021

BPB-8557
TGCAGATTAGCAAGAAACTCTGCTTTCTGTACTTCAACTATTACTCTGTTTTCTAACATTCAAGGGAGGAATTGACATGC
CTGTACTTCCACATGGCATTGTACTTGCGTGAACTCTTGTTTTCGTTTGCAGCATTTAACTGAACCACTTGTATGAATGT
GCTTCCACATGGCATGCTAGGTAGCAGGAGTGAAACACATTTCACTGATATGGAATAGATCGATCGTGCGAGAGCTCACA
GTTCCCTCTGCCGCTTCCATTGCAACCAAGACGGTCAAGAATACTGTATTTCTCCGATGCCGTTTGATCGTCTTACTCCT
GCA

BPB-5374
TGCAGAGACATACACAAAGCGCCCGCTTGCTCGACATGGAACAACTACAAGACCAGCCATAATCTTGTCATGCATGCTCC
CGCAGCCATTAACGCACTGTCTAACATCACTATATGGTTTTTAAATGACTCGTCCCAGTTTTTAGTAAATGCAAATACCA
TGAAATTAATGAAGCACCATAAAATGATGAGATGCTCTTGTACAAGATGATAGACGTTTCTATAAATTTGTAGATACTGG
ATGGACAGCTAATGCATTCACCAACCTTTCATTGTGTAACCCTGACCCGCATTATAAAAGGGTGCACTACCTTGTATTTT
CTTCTTGGACTAGGGCACAAACTAGAAAGAGGCGTGGGTTGGAATGGGACAGTTCATTATGAATGAGAGACATACGGGAA
CACCTCATCCATTATTCCTGACATGGATGTAGAAGCACTACAAATCAATGGCACACGGCGACAACCATACAGGATTGATG
TCACTGCCAAGAAGCTGCA

BPB-7689
TGCAGAGACATACACAAAGCGCCCACTTGCTCGACATGGAACAACTACAAGGCCAGCCATAATCTTGTCATGCATGCTCC
CGCAGCCATTAACGCACTGTCTAACATCACTATATCGTTTTTAAATGACTCGTCCCAGTTTTTAGTAAATGCAAATACCA
TGAAATTAATGAAGCACCATAAAATGATGAGATGCTCTTGTACAAGATGATAGACGTTTCTATAAATTTGTAGATACTGG
ATGGACAGCTAATGCATTCACCAACCTTTCATTGTGTAACCCTGACCCGCATTATAAAAGGGTGCACTACCTTGTATTTT
CTTCTTGGACTAGGGCACAAACTAGAAAGAGACGTGGGTTGGAATGGGACAGTTCATTATGAATGAGAGACATACGGGAA
CACCTCATCCATTATTCCTGACATGGATGTAGAAGCACTACAAATCAATGGCACACGGCGACAACCATACAGGATTGATG
TCACTGCCAAGAAGCTGCACTGGATCCNTCAAGGGGCNAATTCTGCAGATATCCATCACACTGGCNGNNGCTCGAGCATG
CATCTAGAGGGCCCAATTCGCCCTATAGTGAGTCGNANTACAATTCACTGGCCGTCGTTTTACAACGTC

BPB-3623
TGCAGTGTGCTCCTCGGCTCTGACCTACTCATGTGCTGACTGGGCCAAATAGGCCTCCTCTTTGCGTTGTCACGGTCCAG
CTATGCTCGGGCTGAAAATGTTGTGCAATGTTGCAGCTTCTTGGCAGTGACATCAATCCTGTATGGTTGTCGCCGTGTGC
CATTGATTTGTAGTGCTTCTACATCCATGTCAGGAATAATGGATGAGGTGTTCCCGTATGTCTCTCATTCATAATGAACT
GTCCCATTCCNACCCACGTCTCTTCTAGTTTGTGCCCTAGTCCAAGAAGAAAAATACNAGGTAGTGCACCCTTTTATAAT
GCGGGTCAGGGTTACACAATGAAAGGTTGGTGAATGCATTAGCTGTCCATCCNGTATCTACNNTTTATANAAACGTCTAT
NATCTTNTACAAGAGAATCTCATCATTTTATGGTGCTTCATTAATTTCATGGTATTTGCATTTACTAAAAACTGGGGACG
AGTTATTTAAAAATGATATAGTGATGTTAGACAGTGCGTTAATGGCTGCGGCAGCATGCATGANAAGATTATGGCTGGTC
TTGTAGTTGTTCCATGTCGAGCAAGCGGGCGCTTTGTGTATGTCTCTGCACTGGATCCNTC

BPB-7827
TGCAGCAGAGCATGATGCCAAGATTTCCCGCATGATGAGGAAGGAAGGAAAGGAGACAAGTGCATGCAAGACCGTCCAGA
GCTGGGGATCCCTGTCCTGTCTGGTTCAACCACGGCAAGAACCCAAAGCCCAACATCAAACGCCATGCACTATGGCGTTG
GAGTAAGCAGCGGAAACGCCAGTCCTTATAGCGTTTCTTGCTTTGACAGAGACGAGATTGTTAGGCGTATAAGCGTGGAG
CAGAAACATCAAGGTAAATTGATGTTTTTACATGCAGCAGCAACGCCATGGGCTGTGGCGTTTCTAAAAAGGTCAGATCG
TGAAATACTTTCAGACCGACTTCATTCTGTGTATTCTTTTCGTCTCGTGGGTCAGAACAGTGATTTTATCCGCAGGAGTT
AAACACATTTCATTGATATGGAATAGATCGATCATGTGAGAGCTCACAGTTCCCTCTGCCGCTTCCATTGCAACCAAGAC
GGTCAAGAATACTGTATTTCTCCGGTGCCGTTAGATCATCTTACTCCTGCA

BPB-5312
TGCAGAGAGAAAGATAGAGAGAGAGAATCGTGCGGTTTAAACGGGATCACCTGCGCTGTTAATTAATGAGAGAGAGATAG
ACTACCGGTTTGAGATGATCACCTTGTGTATTTATTTGTTTGCGGGTTATTTCTTTCTGTTAATTTTGTTTGACAGCATG
GCAGAGAGAGAGCGAGGGAGATGAGTTAGCAGTTTTTTTTTTTTTTTTGAGCGGGAGCAGTTGTGTACATGGAAAGTTGG
AAACAAATCTTGACGGAATATGATCGATATTTCTTGACCAACAAGATAGTATCTTATTGGAGGCAATGGAAAGCTAATTA
AAACCAGCGAAGATAGTAAAGACAGAGAGAAACCAACATTCAATAAAAACTTCATGAAAGAAATAACATGTACTCCCTCC

ATAAATTAATATGGCGCGTGCTACCAATCAGCCGACTGATCCATGTGTGGGATCAGCCGGCTCGGCGGCCGTTAGATCCG
TCCGTTAGATGCGGTCTGTAGACTTGCCCATGTCGTTATCACCTAGCCTTTTCCAGCCCCCACCACAGTTACTTCTCCCT
GTACACTATTCTATATGACCTACGAGCAACACTGCA

77164
TGCAGTAGCTGCTAATTTTTTTTGATGGTGGAGGGGCACGTCCATCTTTGATGGTTGCCTGTGATTTTAGGTTCTAAGTG
ATATCTGATTTGACATAGTACTGATCCTAGTTCCCCCTATGTGTTTTTATATAGGCGTTTTGCAAAGAGTATAGACAGGT
TGCTCCTCCTGTTCAGTCGAGGTGTCTTTTTGGATCCCGAGATGGCTGGCAAGTAGACAGACAAGGAGCGCGAATGACCT
TTTTAAATTTTGTTGTCGTTACTTGTATCAGTTTGTTTTTTTTTTTTTTGCATGGACTGCTGTAATGTTTTTCAGGAATTT
CTGGACCTAATTGTCTGATGCAATGTTCCATGAAATTGAAATCACGTTGAAGCTATGCTGAGTTTGTTGACCTTATAAAC
ATTTGGAGTACAGTCTCACACTGGCTGGAACGCTGATTCATTTTTTCTCTTCTTTTTTTCCTTTTGAGACTGATGAAACC
GAGTTGAGGCTGCA

BPB-3130
GATGGTCGCTTAGTGGTTACAGCGTACTATCTCGCAGCATAGCGTTGCAAGTTCGAGTCCCGTACCCGCTGCACTTTTTT
GATGATTTCTAGATCCGAAAAATATS

CHR. 4H

BPB-42695

BPB-7645
TGCAGCTACCCTAGTGTGTGTGCGTGTCTGTGTGTGGCTGAGTTTGCCGTTTCTTGGATGGATCTGGCCCTCTCCCATGG
CCATGACTCCTCCTCTCTTCTTGCCTTACTTTCGTTTGCTGTCATCAACTTTTCTCCGTGTTTCCTTTGTGTTTTGTCTG
TGCCGATGATCTCCGCGTTCATTTTGAGGCACTCTTGTGTTGTTCCTGCGTTTCACGGGGTGCCGGTGATGGTTTCCTTC
ATGCATACNTACATAGGTTCCATCTTCCATGTGATGGTTCAACCGCATTATTTACCAGAGGCGCTACTCGTGTTTACTGC
GTGCATTTGCTCTTGTGTACGGAGGGTTGTCTGATTTTTACAAGGCACGGCCGGTGATGAAACAACGATGGCTTTTTGAG
ATGTGCTATGCATGCATATGCATCCACAGATGACAAGACCGAATCAATTTCTTCAAAGCATCTGAGGCCAGCTAGCCAGA
TCTTTTACTGCGTTTGCTGTGATGAACTTGTGGGTGTTAGTCTCTCCCTGTTCTTGGATCTGTCTCTTCTTTGCATCCTC
TTTTTGACGTGCACGTGCCGATGATCCGCGGTAGTCGTTTCTTGGATCTGGTCCTCTCGATCCCATGCATGCATGGCCAT
GAATATAATCCTTTGATCTTGGCTTTCGTTTGCTGTTGATGAACTGTCCTCCATGTGTGGAGGTCTGTTTTAGTTGGCCG
CATGCAAAGGGTTATGATGAACTTTTGCTGTACCACAAATTTCTTGTGTAAGAAGGGTTGTTTGATTTTTACGAGGCCGG
TGACGAGACATCATTGGCTTTCGAGATGTGCTACATATGCATGCACAGATGACATGAGCGAATCAATTTCTTCAAAGTAT
CCGAGGCCTCCCAATCCCAAGCACGACGATTTACTTTGGCTTTGGTTCCATCCTGCA

BPB-14836
TGCAGCACTAGCACTTACTAATTGCAAGATTAGAGGCCAGACATTGCCAATTTTCAGTAGCACCTAGCAGGTTAATACCA
CACCCTTCAGAAAATAAACCCAAGTAGGCAAGTACCACCGCTACCACGCTTGGCGGCAGCACCATTTCTCACCAACTAAT
TAAAGGAATCTCTCGCTCATGAGCTAATTGATTAACAGGTCAAGAGACGCAGCGCGCACCTGATTCCGGTGGCCAACACC
ATCGCCGCTGTGGCCGTCGGAGAAGTGGCACTAGGCACAGGCGGATTCCAAACCCTGCTCTGCTCCCTCTGGATCCCACT
ATGGTGATGGCGAAAGAGAACACCATTAATATTAACCTCCTCTTCTTATTGAGACAACACACAAGCCTTTGCACGTCGCC
CGAAGAAAAATGCCAAGAATAAACTGTCACAAATCTGCA

BPB-9672
CGTCGCGGGAGGAAGATTTGGACGCTGGACATACCTTGATCCCGGATACGCAGTCTACGAAAGGGCGAATTCTGCAGATA
TCCATCACACTGGCGGCAGCTCGAGCATGCATCTAGAGGGCCCAATTCGCCCTATAGTGAGTCGTATTACAATTCACTGG
CCGTCGTTTTACA

BPB-2909

BPB-40823
TGCAGTGGAGATGAAGCTTAGCATCAACAGGATGATGCCAGCAGGAAGAGACCCCTCCATGGCGACACGGAGTCAGGACA
CCATCACTTGCTACGAGCTGGAACAACCTTCTGCCGCTAGTATGTAATAAGGCGCCTTGTAATATCATATGAATGGACCA
GCCATATGTTATTTCCTTAGAATAACCAGCTGAACTCCCTTGCCCCGCTGGATATTATTGTACAATCCACTTGGACCTCT
TTATAGTTCCTACTAGAGATGAAGCACGTATAATTTTCCATGTGGGTTTGCCTTTGATTTGACAAACATGCAAGTTTCTA
AAATGCATTTTCTCCAGTATTTACCAGAATTCATGCGTTTTTCTTATTTCTGTCAAACATGTAAGTTTACAAAATTCCTA
TATTTTCTAAAAGAAGTCCTCTGTTTTGCACTTGTATGTCTATCATATTCTTGTGTCTGTCCTTTCCTATGCTTTCAGAA
TCCCGTGTACCAAAGAGGCCCTTTTGATTGGAAACAGAGACCGGTTTCACATTACAATTTCAATCAACAAGCTTAATGAT

TTCTAATTAGTCTGAAGATCATCATCCACTGACGGTGTTGCCCGTCTCCAGTCATTCAGAGCTGCA

BPB-1469
TGCAGTAGATTAGTGCAATGTCACTAGCATTGTGCCCTGATCCCACGGCCATCCATCCATTATTGGTGCCTAAAGTCTAT
TCCTCTGTCGTGCATTGCATGCTTGATGGTCCTGATCAGAAGAAACACCATCCACTGATCTGTATAAGAAAAGTACGCGT
GCCAACACAAACACAGAGAGTTGGACCTTGTCATCTTATAAGTATGATGATTCACCTTGTTGGTCGTGGCAAACTGATTG
TTCTGTACCTTATTCCAATTCAGAGGTTATACTTTTTGTGCATGAAATACATATTTACCGTTTGATTTTAGAGGTTTTGT
ATCACATCTCCTCTGGAGTTAATTTCTTCCTGACAAAATGAAGACACAGTTATACGCAGCCTAGTTACATACTCTGTGAA
AAACAAATCCGGCACAGCCAAGGAAATAGCTGATATAAGTGATCTCCAGAATTGCCTGACATAACTCATCATGTGGAAAT
TACTCCTTTTCTGTTCCTTTAGTCAGTCTTTTTTGATAGAAATGATGGTGTCCTGATCCCACGGCCATCCATTTATTGGT
GCCTGATTTTTTGAGATCCTCCTTTTTTCCGGTGCCGTGCATTGCATGGTTGGCTGTCCCAATCACAAGAACTTCCAGCC
ACAGCTTCTGTGTAGGAAAAATATGTACGCCAAGACCGAGACCAAGACTTGAAAAATACCGTAGACCATACACAATAATA
TTGTGCTTACGAGTGAAGTCAGGATGAACTACTCTCTCCATTCCATTGTTGTTCTCATCCTGTAGCTAGCTGCTCTCTTC
CTCGTGGTACCAATATGGCTGCCGAGCTTGGGCATCTCGCCCGAGCAGCTGCA

BPB-8569
CAGTGCAGTAGATTAGTGCAATGTCACTAGCATTGTGCCCTGATCCCACGGCCATCCATCCATTATTGGTGCCTAAAGTC
TATTCCTCTGTCGTGCATTGCATGCTTGATGGTCCTAATCAGAAGAAACACCATCCACTGATCTGTATAAGAAAAGTACG
CGTGCCAACACAAACACAGAGAGTTGGACCTTGTCATCTTATAAGTATGATGATTCACCTTGTTGGTCGTGGCAAACTGA
TTGTTCTGTACCTTATTCCAATTCAGAGGTTATACTTTTTGTGCATGAAATACATATTTACCGTTTGATTTTAGAGGTTT
TGTATCACATCTCCTCTGGAGTTAATTTCTTCCTGACAAAATGAAGACACAGTTATACGCAGCCTAGTTACATACTCTGT
GAAAAACAAATCCGGCACAGCCAAGGAAATAGCTGATATAAGTGATCTCCAGAATTGCCTGACATAACTCATCATGTGGA
AATTACTCCTTTTCTGTTCCTTTAGTCAGTCTTTTTTGATAGAAATGATGGTGTCCTGATCCCACGGCCATCCATTTATT
GGTGCCTGATTTTTTGAGATCCTCCCTTTTTCCGGTGCCGTGCATTGCATGGTTGGCTGTCCCAATCACAAGAACTTCCA
GCCACAGCTTCTGTGTAGGAAAAATATGTACGCCAAGACCGAGACCAAGACTTGAAAAATACCGTAGACCATACACAATA
ATATTGTGCTTACGAGTGAAGTCAGGATGAACTACTCTCTCCATTCCATTGTTGTTCTCATCCTGTAGCTAGCTGCTCTC
TTCCTCGTGGTACCAATATGGCTGCCGAGCTTGGGCATCTCGCCCGAGCAGCTGCA

BPB-2837
TGCAGATTTGTGACAGTTTATTCTTGGCATTTTTCTTCGGGCGACGTGCAAAGGCTTGTGTGTTGTCTCAATAAGAAGAG
GAGGTTAATATTAATGGTGTTCTCTTTCGCCATCACCATAGTGGGATCCAGAGGGAGCAGAGCAGGGTTTGGAATCCGCC
TGTGCCTAGTGCCACTTCTCCGACGGCCACAGCGGCGATGGTGTTGGCCACCGGAATCAGGTGCGCGCTGCGTCTCTTGA
CCTGTTAATCAATTAGCTCATGAGCGAGAGATTCCTTTAATTAGTTGGTGAGAAATGGTGCTGCCGCCAAGCGTGGTAGC
GGTGGTACTTGCCTACTTGGGTTTATTTTCTGAAGGGTGTGGTATTAACCTGCTAGGTGCTACTGAAAATTGGCAATGTC
TGGCCTCCAATCTTGCAATTAGTAAGTGCTAGTGCTGCACTGAATCCATCAAGGGCGAATTCTGCAGATATCCATCACAC
TGGCGGCCGCTCGAGCATGCATCTAGAGGGCCCAATTCGCCCTATAGTGAGTCGTATTACAATTCACTGGCCGTCGTTTT
ACAACGTCN

BPB-7275
TGCAGATTTGTGACAGTTTATTCTTGGCATTTTTCTTCGGGCGACGTGCAAAGGCTTGTGTGTTGTCTCAATAAGAAGAG
GAGGTTAATATTAATGGTGTTCTCTTTCGCCATCACCATAGTGGGATCCAGAGGGAGCAGAGCAGGGTTTGGAATCCGCC
TGTGCCTAGTGCCACTTCTCCGACGGCCACAGCGGCGATGGTGTTGGCCACCGGAATCAGGTGCGCGCTGCGTCTCTTGA
CCTGTTAATCAATTAGCTCATGAGCGAGAGATTCCTTTAATTAGTTGGTGAGAAATGGTGCTGCCGCCAAGCGTGGTAGC
GGTGGTACTTGCCTACTTGGGTTTATTTTCTGAAGGGTGTGGTATTAACCTGCTAGGTGCTACTGAAAATTGGCAATGTC
TGGCCTCTAATCTTGCAATTAGTAAGTGCTAGTGCTGCA

BPB-9204
TGCAGTTCGGCGGTGACCATCAGGGATTCAGGGGCCTTCCCTTCATCTATCTTTCTGTCTCGCTATATTTGCTTTCTAGC
TTCATGTGATTAGGCCCATGTTCTACCCTTCTTCTGTGATTGTTTATAGTCACCGGCAGATTTGTATTACTACGGTTGAT
TTATGAAGCACTGTTTTAAGGTTCATGTCTCCCCAATGTCATCCAATCAAAGTCAAAAAGTCAGAAAATTGGACTAACAA
TAGTGTTTCGGTGAAAATACTAAACTGAAGAAGTGAAGAAATTCTAGCCGACCTAACATCGTGAAAGGAATCTTTCCCTA
ATAATAAAGTACACATCGCTTCTGTCGTACGTCGTCGGTCATTTTACATAAAAAACCTTGCGTTTTTTCCAAATCAACCC
GTAGCCCGGATATAAGTGACAAGAACGAACCGCTTTTTGTAGTTTTGCAAATAGGCCCTTCTGTTTTCATGGAATCAACC
CGCAATCCGTTTTGTCCGCATTTTATAGAAAAAACCCTAACTTTTACGATAAATAACCTGCA

BPB-10678
TGCAGTTCTCTGGGCTCCACCTTCTCCAAAAATGTAAGACTTCTCACCACACTTTGGGCACTTGAAGCAACTCATGTTCT

CTACTAAGCCTAGAATCTGCCAAAATATACAGCTGAGTAATGCTGGGAAGAGTGGAAGACTTTGTGAAATATTGGATAGT
GGCACATGCTAGAAATTTAAGCTAATTATTTTTCACTCGTGAAATGAATATGCCTACTGCGATTGAATTCTCATGAATAG
ATATGTGACAGTAATAAACTAACAATAGTAACTGATGCTGGTTGGCAATAACATTAACAAATAAACAGGGAAATGAACGG
TGTGATAGGAATTAGAATTCTATCTGAATTAATGTTTAATATCTAATACACGCAAACGGCTAGAAACTAAGAAAGAAGTA
CAGATATCACAAAACAGAAGTTACATAGAACATACAGGAACTTGGACTTTACGAAACATATTTGCTCCTCTTCTAGCATC
AATTAGAGCAATATCTTGAGGAGTAGAAACAATTAAAGCACCTGCA

BPB-6145
AATTCGCCCTTGATGGATCCATGCAGGTGCTTTAATTGTTTCTACTCCTCAAGATATTGCTCTAATTGATGCTAGAAGAG
GAGCAAACATGTTTCGTAAAGTCCAAGTTCCTGTATGTTCTATGTAACTTCTGTTTTGTGATATCTGTACTTCTTTCTTA
GTTTCTAGCCGTTTGTGTGTATTAGATATTAAACATTAATTCAGATAGAATTCTAATTCCTATCACACCGTTCATTTCCC
TGTTTATCTGTTAATGTTATTGCCAACCAGCATCAGTTACTATTGTTAGTCTATTACTGTCACATATCTATTCATGAGAA
TTCAATCGCAGTAGGCATATTCATTTCATGAGTGAAAAATAATTAGCTTAAATTTCTAGCATGTGCCACTATCCAATATT
TCACAAAGTCTTCCACTCTTCCCAGCATTACTCAGCTGTATATTTTGGCAGATTCTAGGCTTAGTAGAGAACATGAGTTG
CTTCAAGTGCCCAAAGTGTGGTGAGAAGTCTTACATTTTTGGAGAAGGTGGAGCCCAGAGAACTGCACTGGATCCNTCAA
GGGCGAATTCTGCAGATATCCNTCNNNNNGGCGGCNGCTCGAGCATGCATCTAGAGGGCCCAATTCGCCCTATAGTGAGT
CGTATTACA

BPB-1999
TGCAGGCAGCAGTTAGCGTGCATTTTGTTAACTGACGCGTGCAGCGTTCTTAAATGTACCGGCCAGTGTAAGCGCCGGGT
CACAAAAATTTTCTTCGAAATTCTATATACTTTTAAATTTTTGATAAAATTTTGCCAAATTTCGACGAACATGTTTTAAA
ATTAATGGAAGTTTCTCCGAAAATCGGTACACCTTTAAATTCTATGAACTTTCTCGAATATTGACGAGCTATTTTAAAAA
TGATGAACATTTTCAGAAAATGGAAGATCTTTTTTAAAATTGATTAAACTTTTTAAAATGTAAAAAACAATTCAGTTCAA
TGAACTTTTTTTGAAAATACATGAACATTTTTTGAATTTCTATGAACTTTTTTCAAATTAAATGACCTTTTTAAAAATTT
AATAAACTTTTTTAAAATTGATGAACCTTTTTTCAAGATATGAACTTTTTTTGAAATGAATGAACCTCGTTTTTAAATCT
GGAAACCCTTATTTATTTCATGTTTTTATTTTCATAAAAATAGAAAATGTAGACGGTTTTTTTCCTACTGTATCAACAGA
GAACAAAAAAAGAAAAAAAACTGAGCTGCTCACCAGCGTGCTAGTAGGCCGGCCCAAGCCAGCTACTCTAATGGCGCCGG
ATTGTTAACGGGTGCTGCACTGNATCCATCAAGGGCGAATTCCAGCACACTGGCGGCCCGTTACTAGTGGATCCGAGCTC
GGTA

CHR. 5H

BPB-0170
TGCAGATAGAGGAAAGAAGTAATAGTTCCAGCAATTCTTGAATCTACATTTAACTTCATAAGAAAGAAACCCCGAAAATA
GTGTATTTCAAATATCTCTCTGCGAAAGGCAAAAAAGGAAATGTATGTACACCTGATTCGATTTACTAGGCTTTAAAACT
TCCGTTCTATGTGGATAGTGGTTAATTTAAGCATTTAATGTCTCAGCCCAGTTGTATTACTTTTGAGACAGAAACAAGGA
TGGCCTTCAGGATAAACTACTTCAGATGAGTAGTATACCTATGATGATGGACCTCAGTGTAAGGGCGGGTGTCACCCTAC
TTTCTAACAAAGAAGTATAGCTTTGATAGGCAACACACAAGATTTACAGTACATCAAGATGGATGTAATTTTGGACCTTC
CCATATCCATTGAGACAAATTTAACGGAATTATAAAGAAATCTCAGTCCACCCCCTCCAATCACAGCAACACTTAGGTTG
ATATAGTATTACCCGTTTGTTTGGATTTGAAATTACACTTAGGTTACCTTTCGAAGAACTCTTTGATTTTCGATGTATGC
TGCACTGTGACTTTGGCCAAGCATATTATCTCAAAGAACTCTTTGGTTTACGGTGTATATATGCAACATTGTTAATTTCA
GGACGGCAAAAACCAGTATGAAGGAGAGACGCAGCAACAGTGGAAGGAGAGACGGCAAAAATGTTAAACATGCATACAAA
AACATATTGCCGTATACGAAAAATATAGACATCAAAAAATATTTCTAAAAAAACCTAATTGTGTATTCAAAAAAAATGAA
AATGTATAAAATAATACTGGGGAAAACGATACAAACAACACACAGAACAAAAATAAACAAAATTTAAAAGGATCTAAAAC
CAAAGAAAAAAAGAAAACCCATACAAAACGATATAAACAACACTTAGAACACAAAAAATTAAATTCATAAAGTCATAGAA
AAGTGGTGAAAAAGAAAATTAAATGAAGAAGACGGTGAAAGTGAAGAAAAATGAAGGAAATTAAGAAGAAAAAGAAAAAA
CAGAGAAGCCAATAAAGAAAAAAAAAAAGAAAAAAGTGAAAACTGCGGGCTCTACAGGCAAGTGGAGCTATCATCTCGCT
ATGAGCGAGATATAGCTCCCACGATGGTACCGGGTGGCGAAGCGTGCACAAACCTAACCAAGTGGGCCTGCA

BPB-7676

BPB-2591
TGCAGCTGCTAACTAAGCAATATACTATATGCATACATATACAGAGAAGAAAATTGCTTTGACAGAACACCGACAAGCAC
ATGGGAACAGATATACAACAGGAAATGGTAAAAAGTACACTGGAATTTCTTTTGAATTTTATTTGAATACATAAGTTTAT
TTGAATTTTTTCTGTACTCCTTAAAATCAGAATCCGGATTCTTAGATCATGATTCATCTCATCAAGATATTACAATTTGG
ACAGGACAATGGAAGAACTTGGAGCCAACGTTTTTTTTCTGCGATTTGCAGAGCTATCACTATATTATTTTGGAGGCAAA
GATGTCCCTGTACTAAGGAGAGCAAGCTTGAATACATAAGAACACCTTCAAGCTTTATATTGAATCCCTGATTTGTTGCA
ACTACCAAATTGCTAGTTCTGAATTCCAATTAGATAGCTGCATAGTCCAGCTAGCTACACTAGAAGCTAGATCAAGCTAA

TTTCGTGGCACACATGGCTGATGCAACCAATTGGGACTAATAAACAGAAGAGTAATCAAACCACTCTCCTAACCAATTGA
AATACCAATGGTTCTTTGCTTCAGGCAACCACCAAATAGAGCACTTCATGTACAGCTTGTCTGGCAGATAACAGGGAGAG
CAGTAGGAGATATAGAGGACGGAGGGAGATGGATATTGGGAAGTGGGATTAATTTGATGCCCAAACTTTTGTTTAGTTTA
TTTTGGGACTTATTAGCGGGAAGTGAAGGAGAAAGGTTTGGTTGCTCTTTTGATCAGTGAGTCAGAACAAGAACGAGATG
CAACATTTAGAAATTGACCATTAGTATTTAATGCATCTTTATGCTTTTCTTCTTCTATGCATGCGCTCCTGCA

BPB-1084

BPB-2460
TGCAGAGTGAACAAACAAAAAACGGCCCACTACAGCAACAACAAATAACGGGCTGCAAACAAAACAGAGAAGTGGTTCAA
ACCCATGAATCGCGGCGCAAAATGATCGAACGGTTGAGATGGTGACTGAGACCAAACCTGAATCTCAGCTAGCTGAGTTT
CAGCAATCCCGAATGATATTACCCGGGACTGATGCTGATCCAACGGCCAGAAACACATGGCATATAAGTCTTCAAAAATG
TATTGAATATATCCGTTCAAACATTTGACTCACATGTAGTGCATTGTACACTGCCCCAAGCATACATGAGGTGTCCAAGT
TTGGATTCTAGAACCATATTGCATGCTGCCAAATACGACCGTGGAAAAACGCATGCAACCTAACTATATTGTGGCTATAA
ATACCTCCACCGCGAGAGCTGCA

BPB-8072
TGCAGAGTGAACAAACAAAAAACGGCCCACTACAGCAACAACAAATAACGGGCTGCAAACAAAACAGAGAAGTGGTTCAA
ACCCATGAATCGCGGCGCAAAATGATCGAACGGTTGAGATGGTGACTGAGACCAAACCTGAATCTCAGCTAGCTGAGTTT
CAGCAATCCCGAATGATATTACCCGGGACTGATGCTGATCCAACGGCCAGAAACACATGGCATATAAGTCTTCAAAAATG
TATTGAATATATCCGTTCAAACATTTGACTCACATGTAGTGCATTGTACACTGCCCCAAGCATACATGAGGTGTCCAAGT
TTGGATTCTAGAACCATATTGCATGCTGCCAAATACGACCGTGGAAAAACGCATGCAACCTAACTATATTGTGGCTATAA
ATACCTCCACCGCGAGAGCTGCA

BPB-8866

BPB-33276
TGCAGGAGCGCATGCATAGAAGAAGAAAAGCATAAAGATGCATTAAAGACTAATGGTCAATTTCTAAATGTTGCATCTCG
TTCTTGTTCTGACTCACTGATCAAAAGAGCAACCAAACCTTTCTCCTTCACTCCCCGCTAGTAAGTCCCAAAATAAACTA
AACAAAAGTTTGGGCATCAAATTAATCCCACTTCCCAATATCCATCTCCCTCCGTCTTCTATATCTCCTACTGCTCTCCC
TGTTATCTGACAGACAAGCTGTACATGAAGTGCTCTATTTGGTGGTTGCCTGAAGCAAAGAACCATTGGTATTTCAATTG
GATAGGAGAGTGGTTTGATTACTCTTCTGTTTATTAGTCCCAATTGGTTGCATCCGCCATGTGTGCCACGAAATTAGCTT
GATCTAGCTTCTAGTGTAGCTAGCTGGACTATGCAGCTATCTAATTGGAATTCAGAACTAGCAATTTGGTAGTTGCAACA
AATCAGGGATTCAGTATAAAGCTTGAAGGTGTTCTTATGTATTCAAGCTTGCTCTCCTTAGTACAGGGACATCTTTGCCT
CCAAAATAATATAGTGATAGCTCTGCAAATCGCAGAAAAAAAAACGTTGGCTCCAAGTTCTTCCATTGTCCTGTCCAAATT
GTAATATCTTGATGAGATGAATCATGATCTAAGAATCCGGATTCTGATTTGAAGGAGTACAGAAAAAAAATCAAATAAACT
TATGTATTCAAATAAAATTCAAAAGAAATTCCAGTGTACTTTTTACCATTTCCTGTTGTATATCTGTTCCCATGTGTTTG
CCGGTGTTCTGTCAAAGCAATTTTCTTCTCTGTATATGTATGCATATAGTATATAGCTTAGTTAGCAGCTGCA

BPB-47406

BPB-1807
TGCAGCGTCATGATATTCCTTTCTGAAGCTCAAGGTTGTTGTGATATAATTATTCAGCATGCAAGGAGTTAAATGAAGTT
AGACAATGTCAACCATGTGTTTTGGCTCTCCTTTACAACAATGTGTATCTTATTTTTCAGAAAAGGAATAGCTTTACTAC
ACTATAGACTCTGCATCCCAAGCACATAAGTGTACATCATTTGACTTAGTGGAATGTAAATACGCTCCTTTGTTTAGTGA
GATATCAATAAGTTAATTTTCGAACTATGGATCCATTTGGAACTAATGCAGGAAGGTGAACACTATTTAGGACGTTTTTC
ATGGGTATACTTGAAACTGTCATTCAACACCCTTAGCACATTTTTAAAGAGGTTGTGCCTGTATATGTCTAGACCAAAGG
AATACAGTAAAGAGCATATACGGATAATATGAAAGCTTGTTCTTAGATACGGTGTGAGTTGGTTTGAGTTCGGAATATTG
GAAGTGTAGTTCATTTATTTTACTTTGGTGGTTTTAGTTTTGGAACTAATGAAGAAAGGCGATGCTACTTACAATTGATT
TCATGGGCTTACTCAAAACCTGTATTGAACAACCTTATCACGGGACCGGAGCAGAGCAACATTGACATCATCAGTGCAAG
TCTCCGCCCCTTGGCTACTCGCTTATCGCCACCCCTTCTACCAGCACGTCTCTGGAGCAATACTAGCTCCCTTACCTTCC
CAGCCCTGCA

BPB-0091
TGCAGCGTCATGATATTCCTTTCTGAAGCTCAAGGTTGTTGTGATATAATTATTCAGCATGCAAGGAGTTAAATGAAGTT
AGATAATGTCAACCATGTGTTTTGGCTCTCCTTTACAACAATGTGTNTCTTATTTTTCAGAAAAGGAATATCTTTACTAC
ACTATAGACTCTGCATCCCAAGCACATAAGTGTACATCATTTGACTTAATGGAATGTAAATACGCTCCTTTGTTTAGTGA

GATATCAATAAGTTAATTTTCGAACTATGGATCCATTTGGAACTAATGCAGGAAGGTGAACACTATTTAGGACGTTTTTC
ATGGGTATACTTGAAACTGTCATTCAACACCCTTAGCACATTTTTAAAGAGGTTGTGCCTGTATATGTCTAGACCAAAGG
AATACAGTAAAGAGCATATACGGATAATATGAAAGCTTGTTCTTAGATACGGTGTGAGTTGGTTTGAGTTCGGAATATTG
GAAGTGTAGTTCATTTATTTTACTTTGGTGGTTTTAGTTTTGGAACTAATGAAGAAAGGCGATGCTACTTACAATTGATT
TCATGGGCTTACTCAAAACCTGTATTGAACAACCTTATCACGGGACCGGAGCAGAGCAACATTGACATCATCAGTGCAAG
TCTCCGCCCCTTGGCTACTCGCTTATCGCCACCCCTTCTACCAGCACGTCTCTGGAGCAATACTAGCTCCCTTACCTTCC
CAGCCCTGCA

BPB-0351

BPB-8592

BPB-3436
CATGCGTTAGCTAAGGTAGGAGTGTCTACTGATTTATGTAAGGTTTGGTTGCATGCGGATACGCAGTCTACGAAGGGCGA
ATTCTGCAGATATCCATCACACTGGCGGCAGCTCGAGCATGCATCTAGAGGGCCCAATTCGCCCTATAGTGAGTCGTATT
ACAATTCACTGGCCGTCGTTTTACAACGTCGTGNNGGGAAAANNNNGGNCAGTGAATTGTAATACGACTCACTATAGGGC
GAATTGGGCCCTCTAGATGCATGCTCGAGCGGCCGCCAGTGTGATGGATATCTGCAGAATTCGCCCTTCGTAGACTGCGT
ATCCGCATGCAACCAAACCTTACATAAATCAGTAGACACTCCTACCTTAGCTAACGCATG

BPB-1820
TGCAGGACCTCCTCGAGCCTCCTGATGGCCCTGATCAAGCTGCCTTTGAGCACTTGGGTCATCTCCTATTGAGAGATACC
TAACAATCCCCTCCGCGAAGAGGCAAAGATCAGCAAGCTTCAAACCCAAGAATGCAACCACACGTTAGTTGCTTCAGTAC
ATGAACAAAGCAAGTGAAAGCAGATTCGAAGCAACTGAAGGGTGACACCTTCAGAATTTAGGGAAGGAGGTACTACCGCT
ATGATCAACCACTAGTTCTTCCACACTTACTCTATGGATCAGGAATCACAACCGTACATAAAAATTCCCCCTGTTATGGA
ATTTGACTGCTCTTGATTTATCTATTGCTTAACCATATAGTAGAACTCTGCA

BPB-7320

BPB-3892
TGCAGCATCATCAAACCTAGGGAGGATCCCGACGGCGGGGTCGAGCATACGTAGTACTTGCTATTTCAGCTCAACACAGA
GGTTACCACCTGCGGCAGACTCAACGCTGTATATTTTCGGGATCGGACTGAAATGCACACAAGACTTGCGAGAAAACATC
AGACCCTACAGCGCCGGCATGGAGAGAATACTGTATAGCTAGTTACGAGCTTCTGATGCTTGAAGCTGGATACATAGAAC
TACGCACATGATAGCTCTAGGTACGATTCATACGGTCATATCTTGATTACCCTAGAGACATCGTGCATTTACGCTGGGCC
GGTGACAGTTTTCATCAAGGTTTAAATCGCGGACACTGCA

BPB-5075

BPB-0949
TGCAGAGTTCATCTATCCAGCAAACTCAGTTGGAGACCAGTCAATACTATACAATCAAATTAAGAAGGCTGAACTTCAGA
GGTCGCTAGACCCTCCGCCGTTGACAAGCGGGGGTTCTCCGACTCGACCTCGGAACGTCAGTGGACCTGTGGCAGCTTTT
GGTTACAGTGCAACACAACTAGCACTAACACACGCACAACACAATAGTCATAGTTCACATGCTATAATAATGAGCAATAT
TAGTTACAGTTAAGTTTTACTGCACACTCAGAATAACAAGTTCTGAACTCGGAGCTTCAGATTAGCCATCAGTTATAGTT
GGTTTGACTTTGAGCTCACACTTGGAATCACAATTTATAATTTGGCATTTCAGATTAGTCATCTGAACTAATGCACACAA
ACAGCGGACAAATTTCACAGGCCATAACTATGAGCAACATTAGTTGCAGTGAGGTTTTACTTCACAGTTAGAATCACAAG
TTTTAAACTTGGCACTTCAGATTAGGCATCTGATATACCAGCATGCGTGCACAACAGACTAACAATTTACTAGAAAACGG
TGTCAATTCCTAAGAAAGTACTCTACGAAACATTGTCAGCTAGACAAGTAGTAGTATCACTTTGATCACAGTTCACAACT
ACTCTCTCCGTACCTAAATATAAGTCTGTTTAGAGATTTCATTAGATAACTACATATAGAGTAAAATGAGTAAACATACA
ATCTAAAATATGTATACATCCATCCGTATGTAGTCACCTAGTGAAACCTCTAGAAAAACTTATATTTAGAAACAGAGGAA
GTACTGTATTAGGGGGAGGCAGCACAATAGTTAACATCGCGGGCCTGCA

BPB-0786

BPB-6824
NTGCTGGAATTCGCCCTTGATGGANCCAGTGCAGGTGATTCAATCCATCGCTAACGCTTTAGCAACTACTCCCTACGTCC
AAAAATAAGTATCTCAAACTTAATATGATTTATATTAACATTAATACAAAGTTAAGATACTTATTTTCAGACGAAGGAAG
TATAATATCCGCTCATACGTGATTTTCTTTACAAGAAATTGAAACATCACTGATTTTGAAAAAGTTCATAAACACGCAAA
AAAGTTCATGAACCCTATTAGAAAACATTGATAAAAATAGTTCAGAAAAATTTGAAAAAGATCATCAATTTTGTAAAAAA
GGTCATCCATTTTGAAAAATAGATAACAAATTCAAGAAAATTCATCATTTTTGAAAAACAAATCATAAATTTTGTAAAAA
GTTCATCACTTTTAAAAATAGTCATCGAATTGAAAAAAAATCATCAAATTTTGGAGAAGATCTCCGAATTGTTTGAAAAA

GTTCCTGCAATTTGGAAGAAAGTCCATGAACAATGGAAAAGAAGATTCCATTGATTTTGAAAAAAAAATACGCGAATTCAA
AAACAAATCCGTGCATTTAGAAGAAAACAAAAAAAAGGAAGAGAAGAAAAATCGAATAAAAATGTCACCGACCAGTGCAC
TGGGAAAACAAAACGTGAATAGTAGAAAGAATTAAAGGAAAAACAAGATCATTGTTGCAAAACCGTTTGACATACACAGT
GGTTGCAAGTTCAAAACATGGCGGCTGCA

BPB-8924
TGCAGACTTCAGGAATCCAACACCTCCAAAACAACAGAGCAAGAAGCAAGAAAATAGGAACGGTAACACGAATCAGCGAT
TGTCAGCCCGTCATCAAATTAATTACATGGGACTTCTTTACGGTACCAATGGAAAATCAAGCATTTTACTCTGTTTTGAT
CTATCTGCTATTCTATTCTGAGAACAGCAAAGCATTTGTGACCTTCAAAATTCTTAAAAGGTAGCTATCGAAGCCCTTTC
TCATAGTCAAGCAAAGAACAAAAACCATTATCAAGTTTCACATCACGCCATGCAACTAAGAACAAAATCTGTATCACCAT
TCGCCACCACACTATGTCACTACCTCATCAGCAAACGCCTACCTCATCAACAAACGCCACGTTCTCCTCCTAAGTAAAAG
AAGTTACTTCTTCAGATCCGAGGGCTCAGCCTGTATTTTCATTATCAAGAACCACACCGTATACAAAGCTCATACGGACA
AGGAAACACATAAAGCACAAGAAGTAATCCATACAGACTCCTAACAGAATCTGATGTAAAGATCAACAAAGGACGGGAGC
AATATGTAGTCGGAACTGCA

BPB-5207
TGCAGCAAATGCATGCATGCTCCTCGCTTCCGTAGACGTCAGCGTCTCCGCGTTGTGTGGCCATAACTTTACCGCTGTGA
TTCTTGCGTGATGAGGGTTGCCAATGCTGCATAGTTTTATCTGCTGGACTGTTTCACGCTGTTGTCCCTTCTTCACTGTT
GTGGTCACGCCATCATCAACGCAAATGTTCGGTAGAGTCTATGTGCTGATAACGTGTTAAATGTAAAAAGTAAAGATCGA
GGAGAGGTGAATGAAAAATGATTCATTAGTCTTTATTGATGATAGCAATGGGGTATTTATACCCGGGCGGAAGTACACAT
GTTGCTTGGGAGTCAAGTAACGTGAAGTTACTTAAGGGCCAAGTTATTACATAGTTTCCTTGAGAGTCAAGGAAGTGCAT
GATTGCTTGAGGACTAAGTAACCTATCTAAGAATTAACTTAATCCTAATCAGCTTAATTAATAAGCAACTAATCTATTCT
TAACAGTTTTAAAAGTTGAGGCTTGTATATTAGACAATTTTGTAGTTCAGGGTTGTGTTTTAGACTTCGGTGGTAGTTCA
AAGTTGGAATATTCTCTTTTACTCCATCCGTAAACTTTTATAAGACGTTCTAGATCACTAATTTCGTTAGTCAGTTTCTT
GGCCCTTGCCACTTTTTGGATCATTCTCTGCA

BPB-4418

BPB-8637

BPB-8803
TGCAGCAACGAGAACAATCCATCCATGAGATTCTTGCAAACACATGTACCTAAAACAGTTCTGGCCTCTAATAATATAAA
TATGGTCTTTAGATTGATGTGAGTTGGTTTGTCCTCCGATAGTAGTAACATCAAGCACGGCGGTCAATATATTTGCTGAA
ATCAGACAAGTAATCTGGTTCGTTCTACCCATTTGCTTCGAATCTATTCCCTCTGTTCCTAAATATAAGTTTAAAAAAAA
TTACACTACAGACTACATATAAATGTATATAGAGTTATAGACATACTTTAAAGTATAGATTCATTCATATTGCTCCATAG
GTAGTCCGTAGTGGAATCTCTAGAAAGACTTATATTAGGAACGGAGTGAGTAGTATGGGCCCCTTACACAAACTGCAAGA
AAGCTGTGAGCAAGTACCCTTTCAAAGCCCATCCCAACCAGCTTAAGCTCTTCATCAAATGTACTGTCAGTCTGCA

BPB-5271
TGCAGGATTTCAATTTTTGATAGATTTTCGGCCTTTTTCGTCCTAATTTCGTTTGTTTTTCGTTCAAAAATTCAAATTTT
AGGTTAGTCTTTTCGTCTGAATTTTTCCAAAAAAAATCCGAAATTTCTGAATTTTTCCCATTTTGTTTGGAGGCGATAAA
ATTTGCAAAACGAAATCCAAATTGCTGTCAGAGGCACCATAGATATGTGTGGCGGGCGATATACTTCACCTGCCAATGCT
AACTTTTGGAAAATTTGTGGCGTGCAAGGATTTGGCCAGCAAACTGCTGCTTGGAAAATTAGTAGCGACCAAGATGTCGC
CTGCCACTGCA

BPB-6805
TGCAGAGATGGAAAAACAAAAGAAGGCTGCTGAGAAGAGAAGGATTGCAGAGGAAAAGAAAATGATTACAAATAAAAAAT
ACCAGCAGGAGGAGGCAGCTTTTCTAATACATCAAGTAGAGGAGTTTGAGAGGCACGAGAGTTGGAGAAAATTCAAGGAG
GATGAGGAGGCTGCAAGGCAAAATGCTTGGGAGCAACAGAACAATCAACAAGACACGACAGGGAATGAAGAATGGGAGCT
GAGAAAGAGGAGAGCTGAAGAAACAAACAAGAAAGCAATGGATGACAATATGGCAGGTCAAGCAAGGAAGCAAGCTGCGT
TGGAGGAACAAATAAAACAAAGAGCTGCAGCAACTAGTTCAAGGGCCGAGGAGGCAAGATTTAATCCATGCCCTATTCAA
GGAAACAATACAAAGAAAAAAATCTGCACCTACCAATCAGAACATGACATGTTTTAAGAAACCTAGAAAGGTTAACATGTT
CGATGAATTTAGGTAAAGGGACGTGAAGTTTGGAACAATCCTCATCTCTTTTTTTGTGTTTCTGCTTATGATCAGGCGCAA
AACTTGTAATGGCAATATTTTTTTTAGGATCATTGCGTTGATCATGAAGTGGAAGAGCTGTCTTTTATTAGGAAACAACT
ATTACAGACTTTGTGTTATTTGTACTGTGCAACTTAATTGACCAGATTGTTCAAGTGCAAATATAATGCTTATGTGTGCT
TAATTTGTGCAAATCTGATGGCCGGCAACATGCGTGGCAGCTACATGAGTGCGACAGCTGATGGAGGGATTTGGGTGCTG
CA

BPB-2988
TGCAGGATGAGCAATACCAGTATTACAAGAACTTGGATGATGAAAAGAAGTATCTCTTGGTGCTTATGTTGGGTGATTTC
CAAGATGCCATGGTAACCCTATTACCTATCTGTGTTTTTTGAAGATGAATGATGTTTGTACGAGTATCTAGTACTTTAGT
TCCGTTAATTGCAGTCAATTTCTTGACGGGCAGACTATTCAGTTAGTGTACGAAAAATTCCACATGGTTTTGGGGGCAAT
TACAAGTAAGGTCCAGAGCCATTTTCCAGTCTAACATGGTCCCTCTCATAATCTCATTAAGACCATGTTAAATTTGCTCG
TTTCTTAAAGACTAGGAATGCTAAGAGTCAGATATCAGAAAGGCAAAGTCGAGATATACCTTGATTATAACTGTTGTCAT
TTGGTTGCGCATAAACTATCCGTAGAAGAAGTCTTATATCTGTCTGCA

BPB-8809

BPB-9733
NGCCAGTGTGCTGGAATTCGCCCTTGATGGATNCAGTGCAGCTTGATTTTGTCCTCGTGCTTGCCTATAGCATGTCTGAC
GAGGTTGGTGTGTTCTGTGCCCAGCTGCAAAGATGGGGAGGATACCGTCCCTGAAGAACTTCAATGCGTTCCCGCACGCC
GAAGACCACCTGCTGAAGAAGACTTACTCGGGTGCTATAGGTACATAGATACATAATATGCTGCCTCGTGAACTGTTTTC
TGCTAATGGATGCTTATATCTTGGAGATTTGTGTCTAATACGCGCCATCGTTGGTATGTTGGCAGTGACGATTCTCGGGC
TAATTGTTATGGTAACACTTTTCGCGCATGAGCTCACGTTTTACCTTACAACCTATACGATGCATCAGGTACGTTTTGTT
CTTGTTCACCTTTTCATGTAGTTTGGCTAGCATATGTAGCTTATTGTGATGTCTATGTTGTTCTGTCCATTCTTACCCAA
CTTTTATGACTCTAGTTTGTGTTGGGAATATGCCTGTAGATGATTCAAATTTCTCCTGTTTAAGTTTATTCACTATGACT
TACGAAACCAATCTGGCCAAGGAATCTGCATGATGACAAAGGAACTATGAAAGAGAGCAGTTAGTTACATTTATATAAAA
TTGTGTGCCCTTTGATAGTCTCATTGTTGTTAATATCTGCTAGTTGTTGTTTTCATATTAGTGAAAACCAACTTGGACGT
TGTGGTTCTATTTCCCTTGTTCGGAATGCCATCTCAAGTTTTTCTTCAATATCTGCA

BPB-5529
TGCAGATTCGATCGGTCTCGACGAAGTCCACAAAAATAATCCATGGACATGCATACATTCGTAAAAGAGGAAAACTGCAT
CAGGAGATTGTTCAATTTGTCTTTGTTTTCTTGCAATCAATCTAAGATCTAATAACTACTTGTATTTTGGGAATCGCAAA
CAAGAAGTACAGGAGGAAGAACTGTGTATTTCCTTATCAAGAACAGATAAAAATCAAGAAGAACATGAATCAGGAAGCAT
AAATCGAATAAGAAAAAAGGGGAGTCAACTCACGAACTAGTAGCATACTTCACACACGAAGTACACAGATCAAAACTCTT
GAACCTTAATTGCTACAACATGAGTTGATCCTAATCCAATTTTTCTTGTTCTCACACACGAATACTAACCTGCTGGACAA
GGCCAACGGCGACGTAACGCTGTCCAGCGCAACCATGGCCATCGGCCTTCTCGGAGGCGGCCGGCGGCCATAGAAACCTC
CAGGCACTGCA

BPB-5379
TGCAGTGTCTGGAGGTTTCTATGGCCGCCGGCCGCCTCCGCGAAGGCCGATGGCCATGGTTGCGCTGGACAGCGTTACGT
CGCCGTTGGCCTTGTCCAGCAGGTTAGTATTCGTGTGTGAGAACAAGAAAAATTGGATTAGGATCAACTCATGATGTAGC
AATTAAGGTTCAAGAGTTTTGATCTGTGTACTTCGTGTGTGAAGTACGGAATCTGATTTGATGCTACTAGTTCGTGACTT
GACTCCCCTTTTTTCTTATTCGATTTATGCTTCCTGATTCATGTTCTTCTTGGTTTTTCTCTGTTCTTCATAAGGAAATA
CACAGTTCTTCCTCCTATACTTCTTGTTTTGCGATTCCCAAAATACAAGTAGTTATTAGATCTTAGATTGATTGCAAGAA
AACAAAGACAAATTGAACAATCTCCTGCTACAGTTTTCCTCTTTTACGAATGTATGCATGTCCATGGATTATTTCTGTGG
ACTTCGTCGAGACCATCGAATCTGCA

BPB-8822
TGCAGAAAAGGTAGCACCAGAAAACGTGCTACACTGGTGGATTCTACTAATCACCATGGCAATATATTGAATGCAACAAA
TAAACTTAATTTGGACACGGTTCAGTTGTCTCAGAAACCACTATCCCATTCACCATGGTCAGAATGGTACTGTGTTTATA
TTTCAGGAGGCACGGCCAAGTTTGCCATTCGGTACAGGAGTAAGAAAAAGAAAAAAGAAACAGGCGTGTACTTGATTGTG
ACCAAACATAAATTACAGAAATGGATCCATGGAGATATCCGGACTTTTAAGTGCAGGAATGGGCAACTGCCACCAGCCCA
ACATAAGTTGACTCTCACCAGCATATAGACTTGGTACTTGTACAGTTAACCAGAGACTAGGTATGACTTTAACCGTTAAA
GGATAATGGCAACCCCACCATCCATTAGTATTACCATGGATTGCGGTTTACAGTTCTCCACTGAATAGTGACATAGCACA
AAGTAGATGCAACATCAAAGTACAAGAAGATTTACATACCTTCCTTCGGTTTCGGATTACGTGTTGAAGCATGCTCTACA
GCGGGTGATTTTCTCTCAACCATTCTGCTGTCCACCAATAGCTGCA

BPB-1821

BPB-4012
TGCAGGCGGGTCCAAGGAACGCTGGAGCAAACTGAAAAAAAACAAACAGAATGATATATACAGATGCCTCGTATGTTCCA
TTTCCGAACCGTCGATTTCTACGACGCCACAAAATTGTACATATAATACCAATCATAAATTGTAAAACTACTGTTACAGG
TACGCGATTATGCTCGCTGCCATATATGTGGAACACGATAATGCCGAAGAGCAGGGTGAAGCTATTGTGGGGAAGAACCC
ACAATAGAATGATAGGAACTGGAAATAATAGAATGATAGCACTAGCAGATGCCTCGTATGTTCCATTTCCAAAACGTTAA
TTTCTATGACGCCACAAAACTGAACATATAATACCAATCATAAATTGTAAAACCACCGTTACAGGTACACGATTATGCTC

CATGTCGTACATGTGGAACACGATAATGCCCAAGAGCAGGGTGAAGCTATTGTGGGGAAGAACCCGCAATTTCTATTGGC
TTACATGAGCAGTCAGATCGGCATCATGCCATCTGAACTTGCTGCTCTTTTAGGCAAAAAATACACTGTCATGGTTACTC
CAAGCAGCAAATCGCTTGGCGGCAATCACAATTATTTCCCAAATGAAAAAGGCTAAGCCTTTACGGGAGATGTCATCATC
AGTCTTTGGAGCTCAGTACTTCTTCGGTGGCTAAAGAAGATGCCCAGTGTTCTTTCTCAAGAACTTTGTCAGCTGGCATA
CCATAGAGTAGCCTTAACGCATCGCCCCACCTGCCAGCTCGAGCTCAAAATAAATAATATTCCAAATGTACTAGTACTAG
CTATGAACCTTTTAGTTTTCATTGTTGCCATTTTCATGCATTCAAGCAGAGGGTCAAAAAGATTTGAAGAAGAAAGAAAG
CAAAACAACCAAGGGCTTCTCGCATGGGTGAATAAAGATGCAGTCTGCTAAGAATGACAGACAGTATTTCTTAGTCTAGA
CCGTGGACGTTGTGGGTCAAGGTCAATGTAGTTTAGATCATGTAGTCCATGTCATGATTCTGTGCGCTGTATCTAAAGAC
ATGGTCACTGTTACTCCTTATTAGAATGACTACTGAATTATAATCGACACGTCAGTGTTATATTGTGCAAACTCTTTCCC
ATTTTTTAATGTTTTCGCTTCTGCA

BPB-3316

BPB-5182

BPB-2425
TGCAGAAAAGGTAGCACCAGAAAACGTGCTACACTGGTGGATTCTACTAATCACCATGGCAATATATTGAATGCAACAAA
TAAACTTAATTTGGACACGGTTCAGTTGTCTCAGAAACCACTATCCCATTCACCATGGTCAGAATGGTACTGTGTTTATA
TTTCAGGAGGCACGGCCAAGTTTGCCATTCGGTACAGGAGTAAGAAAAAGAAAAAAGAAACAGGCGTGTACTTGATTGTG
ACCAAACATAAATTACAGAAATGGATCCATGGAGATATCCGGACTTTTAAGTGCAGGAATGGGCAACTGCCACCAGCCCA
ACATAAGTTGACTCTCACCAGCATATAGACTTGGTACTTGTACAGTTAACCAGAGACTAGGTATGACTTTAACCGTTAAA
GGATAATGGCAACCCCACCATCCATTAGTATTACCATGGATTGCGGTTTATAGTTCTCCACTGAATAGTGACATAGCACA
AAGTAGATGCAACATCAAAGTACAAGAAGATTTACATACCTTCCTTCGGTTTCGGATTACGTGTTGAAGCATGCTCTACA
GCGGGTGATTTTCTCTCAACCATTCTGCTGTCCACCAATAGCTGCA

BPB-8101
TGCAGAAAAGGTAGCACCAGAAAACGTGCTACACTGGTGGATTCTACTAATCACCATGGCAATATATTGAATGCAACAAA
TAAACTTAATTTGGACACGGTTCAGTTGTCTCAGAAACCACTATCCCATTCACCATGGTCAGAATGGTACTGTGTTTATA
TTTCAGGAGGCACGGCCAAGTTTGCCATTCGGTACAGGAGTAAGAAAAAGAAAAAAGAAACAGGCGTGTACTTGATTGTG
ACCAAACATAAATTACAGAAATGGATCCATGGAGATATCCGGACTTTTAAGTGCAGGAATGGGCAACTGCCACCAGCCCA
ACATAAGTTGACTCTCACCAGCATATAGACTTGGTACTTGTACAGTTAACCAGAGACTAGGTATGACTTTAACCGTTAAA
GGATAATGGCAACCCCACCATCCATTAGTATTACCATGGATTGCGGTTTATAGTTCTCCACTGAATAGTGACATAGCACA
AAGTAGATGCAACATCAAAGTACAAGAAGATTTACATACCTTCCTTCGGTTTCGGATTACGTGTTGAAGCATGCTCTACA
GCGGGTGATTTTCTCTCAACCATTCTGCTGTCCACCAATAGCTGCA

BPB-1241
TGCAGAAACGCGCACGTGACGCATAAAGCCCACCAGAGACATGCCGTTGTGCAAAAGGGCAGCAACTTGACGCACAACGC
GTGGAACTCCCTCGCTGGGTGGCTTCGCCAGCAGTTGTAATTCTTAAAAATGTTAAACAACTTTCTTTCTTCTTTTTTCC
TTTCTGTTTTTATTAAAAGAAATGTTTTTTATTTTCAAAAAATGTTCTTCAAATTCTTTTTTTTGCTTTTTAAATAATTG
TTCTGGATTTTCAAAAAGTGTTCTTGATTTTCAAAGAAAATATCTGGATTTGGAAAAATGTTTGAGAATTGGAAAAGGTG
TTCCAGTATTTGAAGAAATGTCCGGATTTTCAAAAACATGTTTCGGGAATTTGAACTTTTTTTTCGGAATTTTGAATAAA
TGTTTCGGAATTAGATAAAATGTTCCGAAATTTTAGAAAATGTTTTGGAATTTTTATCTAAATGTTTCTGATTTTTCAAA
GAAATGTTCCGAAATTTTACAAAATGTTCCATAATTTGAAAACATGTAGAATTTTCGAAATCGTGAAAACCGTTCTGGAA
TATAGAAATAAGTTTTGAGAACATGAACATTTGTTTCAATTTGTGAATAAATTTCAGTATCGCTAACAGTTCGAAGCATC
AGACGAAACTGCA

BPB-8022
TGCAGTTTCGTCTGATGCTTCGAACTGTTAGCGATACTGAAATTTATTCACAAATTGAAACAAATGTTCATGTTCTCAAA
ACTTATTTCTATATTCCAGAACGGTTTTCACGATTTCGAAAATTCTACATGTTTTCAAATTATGGAACATTTTGTAAAAT
TTCGGAACATTTCTTTGAAAAATCAGAAACATTTAGATAAAAATTCCAAAACATTTTCTAAAATTTCGGAACATTTTATC
TAATTCCGAAACATTTATTCAAAATTCCGAAAAAAAAAATTCAAATTCCCGAAACATGTTTTTGAAAATCCGGACATTTCT
TCAAATACTGGAACACCTTTTCCAATTCTCAAACATTTTTCCAAATCCAGATATTTTTTTTGAAAATCAAGAACACTTTT
TGAAAATCCAGAACAATTATTTAAAAAGCAAAAAAAAGAATTTGAAGAACATTTTTTGAAAATAAAAAACATTTCTTTTA
ATAAAAACAGAAAGGAAAAAAGAAGAAAGAAAGTTGTTTAACATTTTTAAGAATTACAACTGCTGGCGAAGCCACCCAGC
GAGGGAGTTCCACGCGTTGTGCGTCAAGTTGCTGCCCTTTTGCACAACGGCATGTCTCTGGTGGGCTTTATGCGTCACGT
GCGCGTTTCTGCA

BPB-7395

BPB-6579
TGCAGAAATTATTGACTGTCCTATATGGTTCTACACATTAACATAGTTATATTGACTGCACATGGCAACCCCACATGTTA
CTTCATGAACTAGAGGACTGGTAAATGTAGCCAACTTTGCTGATGTATAACCATCTGGGAGCTGTGATATTTTCTAGTGG
GGTGGTGCTGGATGCAGTGGTGCTCCTCACTTAGCATAGTAAAACAGTAATTATAATAGTGATAAGAGNNATGAACTAAT
GATAGTATCTGCTTAACTTTTCCCTTGA

BPB-0882
TGCAGAAATTATTGACTGTCCTATATGGTTCTACACATTAACATAGTTATATTGACTGCACATGGCAACCCCACATGTTA
CTTCATGAACTAGAGGACTGGTAAATGTAGCCAACTTTGCTGATGTATAACCATCTGGGAGCTGTGATATTTTCTAGTGG
GGTGGTGCTGGATGCAGTGGTGCTCCTCACTTAGCATAATAAAACAGTAATTATAATAGTGATAAGAGTCATGAACTAAT
GATAGTATCTGCTTAACTTTTCCCTTGAAACAAATGACTTAAGTTTCTCATGGGAGTGTTTTTTTCTTTTGAGAAAACGC
AAGAAGCCTTTGCGTTTCATTGTATAGAAAAGAGGGGGGGGGGTACATCCTCCTAAAAGGCTGCA

BPB-3879
AGTGCAGCTAGCTACAGACAATGTGATGAATGATTTTGGGTGAGTGATCAGACATTGCGCCAACTCAGGCAACTTGCAAG
CTGCTGTATTTTCCAGTGGGCATTGCAGATTCACAATTAACATGGGGTCCTCGTGCCAGACCGGTCAGTATGGCAATCTC
CAAGCCAAGCTCCATCACACACCAAACACTTGCTCCAGCACAATTGTGGCTGCTCATGGTCTTGTCTCCTGCACATGGCA
GGCCTGAGATATAAAACCATGGCTAAGTATCTCTAAGCACCATCGCTCCATCCACAGGACAAAGCATTCCAAGCTCGAGC
TGACAGAACAATCTCTTCAGTTTTCAGAAACACAAGAAGCTACCATGGTGAGTGCCACGAGACTTGCTCAGCTGGCCAAG
AAATGGCAGAGAATGGCGGTCCTCGGAAGGAAGAGGCTCACTTGGTCATCGGCGGTGCTCGAAAAAGCAATCGAAGGACC
GTGCAGCGCGTCGTATTCGGTGGTGGCTGGCAAGGGCCACTGCGTTGTGTATACGGCTGACGGTGTACGGTTCGAGGTGC
CGCTGGCGTTCCTAGGCACGGTGGTCTTTGGCGAGCTCCTAAGGTTGTGTCAAGAGGAGTTTGGCTTCGCAGGTGGTGAC
GGCAAGATCACACTTCCCTGCGATGCAGCGTTGATGGAGTACGCCATGTGCTTGATTAGGAGAAGCGCCTCCGTGGAGAT
GGAGGTAGCGTTCCTCGGCTCCATGGCAATGACATTAACATGCCACTATGATGCAAGTTGTGTGGTTTCACGTGTGGGAG
TTGCCCAGCATGTTGCTCTCTGCA

BPB-6126
TGCAGGCATCATCTTCTCTTGCTTTACCTTATCGTTTGCTGGTGTGGGTGAAAGAGGAGCAGTGCTTTTTAGCTTTGCAT
CTTTCCTTTGCTTTGCCCTTGCGTGGCTTCCCCTGAATCATATGCATTCATCGTGAGGTATAAGGTATCAGTGGAGGATC
GACACGGATTTTGTTGCGATCAGCCTATCAATTCAACTTTTCATGGCGCGAGCAGATGAGTTGACAAGAGATGAATTATC
AACAAATCAAGAACAAGGAAGCACAGAAGACAAAATATCAGGATCAAGGAGTGATTGTTAGAAGTAATATTGTCTAGCCT
GAGTTAGTCGTAGTACTAGTGGCAAATTCCTAAATATACTACTCCCCCCGTTTCAAAATATAAGTCTTTTTAAAGATTCA
ACTAGATGATTACATACGGAGCAAAATGAACGAATTTACATCTTAAAATGTGTCTATATACATTCGTGTGTAGTATTCTA
GTGAAACTTCTATGTAGTGCCCTTGCACGTCATGTGTTCTAGTAATACAAGTTGTAATCAGGTTTGGTGCTACGGTGGCG
TGCAGGTACAGGTTCCGGTCCTGCTGCATCATCAGCTCGGCCAGCCTGAAGCCTGAAATCTGCAATGTTTTTTTTCAGG
TCATGGTTGCACGCACCATGATATTCTTCCCTGCTAAAACAAATACTCTGGATGATTAGCAAAACAAAAGCACGGCCTGT
GTTCGAGTTTTTAAAACCAACGAACTCTTACATGATCGGTGCCTGACTAATACCACCCTGCTCATCGGCTGCA

BPB-4583
TGCAGGGAGATCAGGAGACAATGAGGATTATGCTATGTATGAAGAGGAAACGCCCCTACATATTGATATCAGAAGGTTCA
CGTACGCAGAGCTGAAGCACATAACAAACAACTTCCATTCAATAGTTGGAAAAGGAGGTTTTGGAACTGTTTATCATGGC
ACAATGGAAAATGGTGATGAAGTAGCTGTTAAGGTGCTTATGGAGACATCAATAGCGGAGTCAACAGATTTCCTCCCTGA
GGTATACCACAAAACCAACTAGGTCAAATCAATAATTTCTTCAATAGAAAGTAAACTATAAGTGTGCAATATATCAACCC
TTTCCAGAGTCCATATCCAATTAAATAATACAATTGTCTTCAGCTCTAGGATGATCGGTAACTATATCTGTTGCTGCA

BPB-6135

BPB-0710
TGCAGGAGCAACCATGCATTCATATAGACACCACCGGGGGGAGGGGAGTGTTCAAGTAAGAACACAAATGTGAGAAAGAC
TCTGACTACAAGGAAGCATAAGAATCAACCCAGACCCTGAGGAGGGTCAGAGATGCCTCCTTAAATCTAAAGGATTGAAC
ATGGACTTCTTTTAGGACGAGCCATTGTCTAATAGCGGCTATTTTGCCAAAAAAATCTATGTGTTCCTTTGTTTCCAGAT
TAAGCCAACAGGCCACGACAAAGATGCCTAAGAATCAATTCCCTGATAAACCAGCAGTACAGTGGTGCAGGATGTCTTCC
ATACCGTTTCTCTGCCGGTCTGCACAACTGAATGCTAAGCTTGTTCGAATAGTTGACATTAAAGGGGCCTCTAAAGGAAA
GATAGATGCTGGTTTTCCATGCCATTTCTACAGCCCGTTGCTGCAAAGGACACAAGGGTTACCATTGGAGAAAGTGAAGC
GCCGCCGCGCAAGAATTTCTTTGTGTTTACATGATCAAACTAAATCTATGTCCAGAAACTCAATGCTGCA

BPB-3241

TGCAGCCGAATCCAATAGGTCTTAACTGGTGTGTAACTTACTGTTCCAAACGCCTGCCAAAGCAGTCAAAGTAGTATTAC
AGCGTAGTATATTTAAGGAACCTGCATTTTGCATAGAGCACACCTAGGCGGGTTTATTCTTGCAATTAAGGCCCTGTTGT
TTCTTTAAAAAGTCTTAGGACTTTTTTAGTCCCAACTAAAAAGTCTTTAGTCCCTACCCGTTTCTTTCCAAGGACTAAAC
ATGGACTAGAGGTCACTAAATGACATGCAAAAAGATCATGTTACTCCTAGTAATATACTAGAAGTTATTAAATGACATGC
TAAAAGTAGGGGCATTGTTGAAAAAAAAAGCCAAGAAAGTCTCAAAAAGTCCCTCCCCATAGAGACTTCTTCTTTTAGTCC
CAAATACCCCTTTTAGTCCCTAAAAGTCCCTTCTGTTTCTTTCACATGGGACTAAAAGGGATTTTTTTAGTCCGTACAT
CAAAAAGTCACTGGAAAGAAACACCCCCTAACAGTACTGATATTAGTGGGCTCGCTACTTGCCCTGTGAGCTTACTGCTG
GTTACCTGCA

BPB-4115
TGCAGGTAACCAGCAGTAAGCTCACAGGGCAAGTAGCGAGCCCACTAATATCAGTACTGTTAGGGGGTGTTTCTTTCCAG
TGACTTTTTGATATACGGATTAAAAAAATTCCTTTTAGTCCCATGTGAAAGAAACAGGAGGGATTTTTAGAGACTAAAAG
GGGTATTTGGGACTAAAGGAAGAAGTCCCTATGGAAGGGACTTTTTGAGACTTTTTTGCCTTTTTTTCCAACAATGCCCC
TACTTTTAGCATGTCATTTAATAACTTCTAGTATATTACTAGGAGTAACATGATCTTTTTGCATGTCATTTAATGACCTC
TAGTCCATGTTTAGTCCTTGAAAAGAAACGGGTAGGGACTAGAGACTTTTTGGTTGGGATTAAAAAAAAGTCCTAAGACT
TTTTAAAGAAACAGGGCCTTAATTGCAAGAATAAACCCGCCTAGGTGTGCTCTATGCAAAATGCAGGTTCCTTAAATATA
CTACGCTGTAATACTACTTTGACTGCTTTGGCAGGCGTTTGGAACAGTAAGTTACACACCAGTTAAGACCTATTGGATTC
GGCTGCACTGGATCCGTCAAGGGCGAATTCCAGCACACTGGCGGCCGTTACTAGTGGATCCGAGCTCGGTACCAAGCTTG
GCGTAATCATGGTCATAGCTGTTTCCTGTGTGAAATTGTTATCCGCTCAC

BPB-8771
TGCAGGATGCAGCAATACACTTCGATGTTTATATTTGTGTCTTGTTTTTCAATGTCATCTTCTGCATGCATTTGTACATG
TCAGCCTGTCAAACTGTAGTATGTCACTGTTAATCCTTACTTGATTTGCTTGCCTGTCACCATTATCAGCCTGTCTCACA
TTTCTGCTAAGCTACTTGGTTATTTGTTATCCTAACTAACATGACTCTTGGTTATTGGTTATCCCTTCTAATCTTGCTAT
AGATTATCCGTTGTGCTCTTCTGCCTGTTTGATCTGAGTATTATTGGAACAATATCTGCTACTCTGAATTTGTGCTGAAT
CATATTAAAAGTAGTTATATTTGACATTTGGTGTGCCGTGATTGATGAATTTGCGATGGTTATGTTATTTTTATCCGGCC
TGTTGTGCACCGTTTGNNCTACTTTACCTAGATCTGTTGTTTTTGGCCAGTGNTTGCCAAAGGCATAAAAGTAGTCACAA
TTGAATCAAATA

BPB-8731
TGCAGAGAGAGAGAGCATGTTCTGTTCTGCTTCTACAAATCCTGAACCAGATTTTCTAAGCACCTCCGAAAAAGTTAATA
CAATTTCATGAAAAAAAAGATCACAAATTCACAAAAAAAATGTTTACAAGTTCAAAAAACACTTCATGAATTTGAAGAAA
GTTCATACATTTTAAAAGTTGTCAAAATTTGAAATAGTTCAAGCATTTTCATAAAGAATAAAGAAAGAACATAAAAACAC
AGTTCATAAAAATTAAATGAAAAAAGAAATGAAAGACCTTTTTATTTAAAATAAGTTTACAAAATTTCTCCACGAATCTG
ATTTTTTTTGGTTAGTTTCAAAATAGTTTTTGGAATTGAGAAAAGTTCAAATAATATAAAATACATAAAAAATTCATGAAT
TTGAAAAAAATTAAAATGAACAAAATTTCATAAAAAAAATAGCCAATTTTAAAAGTTCACAAATTGAGAAAATGTTCATGC
ATTTGAAAAAAGGGAGAACGAAAACGAAAATGATAAAAACAAGACAAGAACGGGCCGAAAAAATGTGAAACAAAAGGAAA
ATGTTTAAAACAACTGGATGATATCTGAAGGAAATAAGTCGTCTTTCCCGCGTGACATGCGTCCTCCTAGGGCCACGCAC
CGCGTCGCTGCTTCCCTTATCCTTATCCTTAGCGTCAACGCGAGCCGCTCATTCCTTCTCTTCTATCCAGTGAGATCGCT
ATTCCTCTTCCATTTTCTAGTTCTGCCACACAGCTAACCATCTTTTGCATGCTATCAGCCCTCGTCGGAGCAGCTGTGGG
CGGCTTGTAATGTTCCAGCGGCCGACAATTTGTAGCAAACCTGATTCAACCTGCA

BPB-3700
TGCAGAGCTTGCGGACCTCTCCCTGTGTGCCGGTTGCCTTGCACATGTGAGACTAAATATTTCCACATGTTGCCTTGCAC
AAATGGGCTGCGTGGGCCTCTAGGATTTATTTAGGGATTTCTGAAATTATATATTGGGCTGGCACAAAACTAGATAAAAT
TCCAGCATGCAATGCGCCTTAAACCTTGTATATTATGAAAATACACATAAATTTTAACCGCGTCTTCTAAACCGCGACGG
AGGTAGTATATATAGTAGAGATATGTGACTGAAATGTATATACGTGCACAACATCAATCCTGTTATTGTTGATCTCGATC
ATATTCCCTCCGTTCCTAAATATAAGTCTTTAAAGAGGTTTCATTAATGGACTACATACGGATGTATATAGACATACTTT
AGAGTGTAGATTCACTCATTTTACTCCGTATGTAGACTTCTAGTGAAATATCTTAAAAGACTTATATTTAAAAACGGAGG
GAGTATAACGTAACATTTTATTAGAAAGTGATCAATGCCACCCAAATCAAAAGAATGGAAGGTGCGGTCAGAAGTAAGTA
CATCAAGATGAGTCCGAATAAAACCAAAAAGGAGATAAAATACAACCAATCCGACTGGAAATAAACACAAACGTCTCGCC
ATTATCGGATGTTTCCTCAGAAAATGACGACGATGATATCATCAGTTGAACTTGCTGCA

BPB-3910

BPB-8462
TGCAGAGCTTGCGGACCTCTCCCTGTGTGCCGGTTGCCTTGCACATGTGAGACTAAATATTTCCACATGTTGCCTTGCAC

AAATGGGCTGCGTGGGCCTCTAGGATTTATTTAGGGATTTCTGAAATTATATATTGGGCTGGCACAAAACTAGATAAAAT
TCCAGCATGCAATGCGCCTTAAACCTTGTATATTATGAAAATACACATAAATTTTAACCGCGTCTTCTAAACCGCGACGG
AGGTAGTATATATAGTAGAGATATGTGACTGAAATGTATATACGTGCACAACATCAATCCTGTTATTGTTGATCTCGATC
ATATTCCCTCCGTTCCTAAATATAAGTCTTTAAAGAGGTTTCATTAATGGACTACATACGGATGTATATAGACATACTTT
AGAGTGTAGATTCACTCATTTTACTCCGTATGTAGACTTCTAGTGAAATATCTTAAAAGACTTATATTTAAAAACGGAGG
GAGTATAACGTAACATTTTATTAGAAAGTGATCAATGCCACCCAAATCAAAAGAATGGAAGGTGCGGTCAGAAGTAAGTA
CATCAAGATGAGTCCGAATAAAACCAAAAAGGAGATAAAATACAACCAATCCGACTGGAAATAAACACAAACGTCTCGCC
ATTATCGGATGTTTCCTCAGAAAATGACGACGATGATATCATCAGTTGAACTTGCTGCA

BPB-4058
TGCAGCGAATTAAATTATTCACTACAGAAGCAAGCTCGAATTGGGCTTGTAGTTGGTGGCGCACCTGCACATAGAAGACA
CTATGGGCATGGAGGACAGAGAGTTGTAGATGTGGAAGCAATTGTCGCCATGGCGTGGCGCAAGTGGTGAAGACCCGTT

BPB-3887

BPB-1420

BPB-4970
TGCAGATATTGAAGAAAAACTTGAGATGGCATTCCGAACAAGGGAAATAGAACCACAACGTCCAAGTTGGTTTTCACTAA
TATGAAAACAACAACTAGCAGATATTAACAACAATGAGACTATCAAAGGGCACACAATTTTATATAAATGTAACTAACTG
CTCTCTTTCATAGTTCCTTTGTCATCATGCAGATTCCTTGGCCAGATTGGTTTCGTAAGTCATAGTGAATAAACTTAAAC
AGGAGAGATTTGAATCATCTACAGGCATACTCCCAACACAAACTAGAGTCATAAAAGTTGGGTAAGAATGGACAGAACAA
CATAGACATCACAATAAGCTACATATGCTAGCCAAACTACATGAAAAGGTGAACAAGAACAAAACGTACCTGATGCATCG
TATAGGTTGTAAGGTAAAACGTGAGCTCATGCGCGAAAAGTGTTACCATAACAATTAGCCCGAGAATCGTCACTGCCAAC
ATACCAACGATGGCGCGTATTAGACACAAATCTCCAAGATATAAGCATCCATTAGCAGAAAACAGTTCACGAGGCAGCAT
ATTATGTATCTATGTACCTATAGCACCCGAGTAAGTCTTCTTCAGCAGGTGGTCTTCGGCGTGCGGGAACGCATTGAAGT
TCTTCAGGGAC

BPB-4318
TGCAGATATTGAAGAAAAACTTGAGATGGCATTCCGAACAAGGGAAATAGAACCACAACGTCCAAGTTGGTTTTCACTAA
TATGAAAACAACAACTAGCAGATATTAACAACAATGAGACTATCAAAGGGCACACAATTTTATATAAATGTAACTAACTG
CTCTCTTTCATAGTTCCTTTGTCATCATGCAGATTCCTTGGCCAGATTGGTTTCGTAAGTCATAGTGAATAAACTTAAAC
AGGAGAGATTTGAATCATCTACAGGCATACTCCCAACACAAACTAGAGTCATAAAAGTTGGGTAAGAATGGACAGAACAA
CATAGACATCACAATAAGCTACATATGCTAGCCAAACTACATGAAAAGGTGAACAAGAACAAAACGTACCTGATGCATCG
TATAGGTTGTAAGGTAAAACGTGAGCTCATGCGCGAAAAGTGTTACCATAACAATTAGCCCGAGAATCGTCACTGCCAAC
ATACCAACGATGGCGCGTATTAGACACA

BPB-6179
TGCAGCAGCACCGTTTTAGTTAATGTTCGGATCCACATAAAGATTCACAATTAAATTATAAAAGAACATAAATAGGACAC
TTTGATAGGTTATATCTCAAAGTACATATGCAATTTATATGCAAAAGAAATATGTAAACTAAACTTGTACAACACAAATA
TTATATGCACCTTCGGTTCATTTTTGAAAATACCAGATAATTGGAGATTTGATGTGTATAGTTAGAAGAACCAACATGTC
TCTACATAGAAAATAAGTGTTGGCCATGCAAAAAACATCCGTAATCATGTTTGCTAGAAATAATAGTGGACTATATTGGC
GTCGTTTGATTGGACTTGAAAGTTTGCTCAATAAAAATCCTATGAAATTCCTTTGAATTAAAGGAGCCCTTATTTCCTAT
AAGTTTCCTATTCCTATAATAATTAATATTACCAAAGGAGACCTTATATTTAGGAACGGAGGGAGTAAAACCCTTCGTCA
CATTTATCGAAGGTTCTTTTGCGGTGAGTTTTCTTTGTCATTGTGATAGTCGACGTCAGTGTTTCACTGCA

BPB-4595
TGCAGCAGCACCGTTTTAGTTAATGTTCGGATCCACATAAAGATTCACAATTAAATTATAAAAGAACATAAATAGGACAC
TTTGATAGGTTATATCTCAAAGTACATATGCAATTTATATGCAAAAGAAATATGTAAACTAAACTTGTACAACACAAATA
TTATATGCACCTTCGGTTCATTTTTGAAAATACCAGATAATTGGAGATTTGATGTGTATAGTTAGAAGAACCAACATGTC
TCTACATAAAAAATAAGTGTTGGCCATGCAAAAAACATCCGTAATCATGTTTGCTAGAAATAATAGTGGACTATATTGGC
GTCGTTTGATTGGACTTGAAAGTTTGCTCAATAAAAATCCTATGAAATTCCTTTGAATTAAAGGAGCCCTTATTTCCTAT
AAGTTTCCTATTCCTATAATAATTAATATTACCAAAGGAGACCTTATATTTAGGAACGGAGGGAGTAAAACCCTTCGTCA
CATTTATCGAAGGTTCTTTTGCGGTGAGTTTTCTTTGTCATTGTGATAGTCGACGTCAGTGTTTCACTGCA

BPB-0835
TGCAGTGAAACACTGACGTCGACTATCACAATGACAAAGAAAACTCACCGCAAAAGAACCTTCGATAAATGTGACGAAGG
GTTTTACTCCCTCCGTTCCTAAATATAAGGTCTCCTTTGGTAATATTAATTATTATAGGAATAGGAAACTTATAGGAAAT
AAGGGCTCCTTTAATTCAAAGGAATTTCATAGGATTTTTATTGAGCAAACTTTCAAGTCCAATCAAACGACGCCAATATA

GTCCACTATTATTTCTAGCAAACATGATTACGGATGTTTTTTGCATGGCCAACACTTATTTTCTATGTAGAGACATGTTG
GTTCTTCTAACTATACACATCAAATCTCCAATTATCTGGTATTTTCAAAAATGAACCGAAGGTGCATATAATATTTGTGT
TGTACAAGTTTAGTTTACATATTTCTTTTGCATATAAATTGCATATGTACTTTGAGATATAACCTATCAAAGTGTCCTAT
TTATGTTCTTTTATAATTTAATTGTGAATCTTTATGTGGATCCGAACATTAACTAAAACGGTGCTGCTGCA

BPB-1719
TGCAGTAGTTAACCTATAGAAAGAGTATTGTTAATAGTTTCTTATTTTGATTGGCTTGCCAAAGCCAAGCCAGACAGGGG
ACGACAAAGAGTCTCATGTTGTTTGCCAATAGAGACCCATCATGATGATTTTTAGGCAGGGGGAATGGTAGTTTCCATGT
GTTCCTCTATTCCCTATTGTTGACAAGTTATTTCTTCGGTACTTCTAACGCTGACGTTGGTATTCCTTTTTCGGTGGTTT
ATCTTACGGATCTACAGACACGGAAATCGTACGTGTTTATGCGTACTGGTGCTGGTCGGGTGCTCGTTGAATCAGATGCT
GGGAGGGGGCAGGTCTTGGAGCTGTTCCGGTAGCAAACTCCACAGGAAACTTCGGCTGCATCTGGGCTGCA

BPB-0799

BPB-5766
TGCAGTGAGCTGGATGATGAGAGCTGCATTACACGCTCCTCATAGTCATGCTTGACCTCTCCAAGTATCTTCAACACAGA
AACAGCTGGGAAACAGTTTAATACTTTTGAAGATGATGATTTGCTGCTGCTGCTGCTAAGATGAGATACATCTAATTCGA
GACTCTTAACTGAACGGAACACACTACTGCCATCCAGCCCTTCAGGGAACAAACCGTCGCATCTTCCGACAGTCATATCT
TCTACTTTATCCAGATTGGGGGAGGCCAAACCACCGTCATACCCTCCTGTAACAGCCAATTTCTTTCCATCATAATAAAA
AAACGTTCCCAAATCAGGTCGCGGAAAGCCTTTTCTCCTTGAATTATCTCTTTTAACAACAAAATATGTCAGTTTGGAGG
TGTGAGGCATGGAAGGCAGAGACATCTTGGGGCACCCAGAAACTTCAAGTCTGCGCACATTGGTGCAACATACCTCCGAC
AAGGGCATCACACGGAGATTGGGACAACGGACGCATTCGATTATTTCAAGACTTGGAAAGGAACAACAATTAGGTTCCAC
AACCCATTCAGCAAGTTCTGGCATCTCATAAAACTCAACTTCCTTCAAGCGCATAAAACATTTGCCTGAAGCGCCACCAC
AGACAGGCCCAAACTGAAGCATTCCAGAAATTTTCCTCAATTTGAGTCCCTTGAGATTTGGTAGCTTCGCAAAAGGTGGG
AGGGTGCTCCNAGATACGCCTTGTAGAGTGAGAGTCTCTANACTTGTT

BPB-7360
TGCAGGTTAACAAGTTTAGAGACTCTCACTCTACAAGGCGTATCTTGGAGCACCCTCCCACCTTTTGCGAAGCTACCAAA
TCTCAAGGGACTCAAATTGAGGAAAATTTCTGGAATGCTTCAGTTTGGGCCTGTCTGTGGTGGCGCTTCAGGCAAATGTT
TTATGCGCTTGAAGGAAGTTGAGTTTTATGAGATGCCAGAACTTGCTGAATGGGTTGTGGAACCTAATTGTTGTTCCTTT
CCAAGTCTTGAAATAATCGAATGCGTCCGTTGTCCCAATCTCCGTGTGATGCCCTTGTCGGAGGTATGTTGCACCAATGT
GCGCAGACTTGAAGTTTCTGGGTGCCCCAAGATGTCTCTGCCTTCCATGCCTCACACCTCCAAACTGACATATTTTGTTG
TTAAAAGAGATAATTCAAGGAGAAAAGGCTTTCCGCGACCTGATTTGGGAACGTTTTTTTATTATGATGGAAAGAAATTG
GCTGTTACAGGAGGGTATGACGGTGGTTTGGCCTCCCACAATCTGGATAAAGTAGAAGATATGACTGTCGGAAGATGCGA
CGGTTTGTTCCCTGAAGGGCTGGATGGCAGTAGTGTGTTCCGTTCAGTTAAGAGTCTCGAATTAGATGTATCTCATCTTA
GCAGCAGCAGCAGCAAATCATCATCTTCAAAAGTATTAAACTGTTTCCCAGCTGTTTCTGTGTTGAAGATACTTGGAGAG
GTCAAGCATGACTATGAGGAGCGTGTAATGCAGCTCTCATCATCCAGCTCACTGCA

BPB-4725

BPB-9244
TGCAGGTTAACAAGTTTAGAGACTCTCACTCTACAAGGCGTATCTTGGAGCACCCTCCCACCTTTTGCGAAGCTACCAAA
TCTCAAGGGACTCAAATTGAGGAAAATTTCTGGAATGCTTCAGTTTGGGCCTGTCTGTGGTGGCGCTTCAGGCAAATGTT
TTATGCGCTTGAAGGAAGTTGAGTTTTATGAGATGCCAGAACTTGCTGAATGGGTTGTGGAACCTAATTGTTGTTCCTTT
CCAAGTCTTGAAATAATCGAATGCGTCCGTTGTCCCAATCTCCGTGTGATGCCCTTGTCGGAGGTATGTTGCACCAATGT
GCGCAGACTTGAAGTTTCTGGGTGCCCCAAGATGTCTCTGCCTTCCATGCCTCACACCTCCAAACTGACATATTTTGTTG
TTAAAAGAGATAATTCAAGGAGAAAAGGCTTTCCGCGACCTGATTTGGGAACGTTTTTTTATTATGATGGAAAGAAATTG
GCTGTTACAGGAGGGTATGACGGTGGTTTGGCCTCCCACAATCTGGATAAAGTAGAAGATATGACTGTCGGAAGATGCGA
CGGTTTGTTCCCTGAAGGGCTGGATGGCAGTAGTGTGTTCCGTTCAGTTAAGAGTCTCGAATTAGATGTATCTCATCTTA
GCAGCAGCAGCAGCAAATCATCATCTTCAAAAGTATTAAACTGTTTCCCAGCTGTTTCTGTGTTGAAGATACTTGGAGAG
GTCAAGCATGACTATGAGGAGCGTGTAATGCAGCTCTCATCATCCAGCTCACTGCA

BPB-7292
TGCAGGCATGGATGAAACAATCATAAGAAGATGCTGTGAGCGAAGCTAGCTGGTGGTATACCACTACCACGTGCACTAAA
CTCATTGCACGTGATGTGAGTTAAGCTCACAAGTTATTCAGCATTCTGAATTAGCCAATTGATCATGGATTTGTGGTCAA
TATACATCTTCACAATCTCTTCTGCTGCCTCTCTAGCTAGTGAGGGAGTGAGTGACACTGACCAGACTAGCTAGCTAGGT
AGTGACATCCTTTGCAGTAGCCAATTGATCAAGGAGGGGGACAAGTCACAGAATTAATCTACAATTCGCCCACAGGCTGC

TCCGTCATCAAGTCAATGTAGCCGCTTGGTTTTCACTCAGTTACATTGGGATTACCTTACCATTCTGCA

CHR. 6H

BPB-5926
TGCAGGAGTATGTAGCTCCTCCAAAAACTTATTTGTACAAATGATATAATTAGCACATTTTCATAACCTTTTCATTCATT
ACTGTATATTTCCGTACCGTGTAAACCGAAACATTTTTCAAACAGGATAGAAGTCATATTACAACAACCTTTTCTTCATT
TCGTGCTTCGGGATTAGTCTTTGCAGCAGTATGTGGCTCCTTTTCTATAGAAATCATACATCTTTAGCACCATTTTCGTA
CCCTCTTTATTCATCACTCTATATTTTCACTTCATGTCAAAATGAAAATATATTTGTATAGCATCTCCACATATTCAACT
CACATTTCCATCATTTCAATCTTCAAGAGCAGTTGCCGTTGCATTATATACCTCACACAACGACTTACATGTGGAAATCG
GACAACTTGTAATATTATTCGTATTGTTTCGGTCCAACATGGTATATTTTCTCTTATCTGAAAATAAAAATTTTCAAATA
GACGAATCGACATATGCTAGTAATCCCGCTTCATTTCTTGCTTTCGAATATGTCTCTACAGCAAGATGTAGCTCCTCCAA
CAACTTATATATGGAAATGATATCATTTAACACTTTTCTCGTAACCGTTTTATTCATTACATATAGATTTTCTCTTCCCG
GGGAAGTGGAATATTTTTCAAATAGGGTAGTCGCCATATTACAACATCATTTTCTTCATTTCATAGTTTGCCACTACTTT
GTGCAGCAGTATGTGGCTCCGCCTTATGTAGGAATTATATATCATTAGCATCATTTTTGCATCATCTTAGTCAGTCACTT
TAGATTTCTGTTCGTCTAAAACTGAAAATTATATTATATGGCATTGTCGATAGATAAGAGTAGCTTTCGACCAATTCTAT
ATTACATCATAGTTTTTGTTGCATGATGTACCTCAAACAACAACTTATATGTAGAAATCGGACAACTCGTAATATGTTTT
GTACCATTTCGATCCATCATGGTATATTTTCTCTTTTGTGAAACTGAAAAGTTGCTTAAATAGGAAAATTTACATATGAT
ATCAGTCCTGCTTCATTTGTTGATGTCAAAGTTGTCTCTGCA

BPB-4754
CATGGCAAGATAAAAAAAGTTGTTATGATTGTGAGCCGTGATAATTTGAGTCCACCAAATCAATGTGTCAGGTCTACATG
CGGATACGCAGTCTACGAAAGGGCGAATTCTGCAGATATCCATCACACTGGCGGCCGCTCGAGCATGCATCTAGAGGGCC
CAATTCGCCCTATAGTGAGTCGTATTACAATTCACTGGCCGTCGTTTTACAACGTCGTGANTGGGAAAACANNGGCNAGT
GNNTTGTAATANNANNTCACTATAGGGCGAATTGGGCCCTCTAGATGCATGCTCGAGC

BPB-2380

BPB-8937
TGCAGTAGATACACACATGACCTTTTATTTTTTTATAGAAAATTCATGACCTAATTAACATGGCAAGATAAAAAAAGTTG
TTATGATTGTGAGCCGTGATAATTTGAGTCCACCAAATCAATGTGTCAGGTCTACATGCAGTTTGGCAATAGAGTAACAT
AAATCATCACGTAAAAAAAATTCTTTCATTGCAAAAAAAGAAATCTGGAGTCTCCAAATAGATCTACACCATAATAACTC
AGGCCCACCGGATAAACGGCTCTCAACGTGTGAATTTCTTGGGAGTCTCTAATTAGTATTAGTATAGAAGTATAGATTAA
CAACCTCTGCAACAATTTATTAAGCAAACGTGAGTACAAAAGTACTCAGCAAGACTTTGTGATAAACTATCTACTCATGC
AATGTATCAATAAGGAATTGTGGGGTTTCATGCGGAAAGCCAACATTTGACTCATGGCTAGACAAGTTGCATTTTCAAAT
AGTTTAGACAACTTTGATTTCTCGCACACGAGTCCACTAACACCACAACAATACTCCATCATGGAACCATTCCGTCTCCA
TACGGAAATGCCGTCCACGACACTCACGCTTATCTTGACAATTTTATGAGTACCAACTATAGTTATCTATGAACAACATA
TGTTTCCAAGTAGTCCATATCCGCGGACGTAGCTATTCGAATAGATCATAACCCTGCA

BPB-5433
TGCAGGGTTATGATCTATTCGAATAGCTACGTCCGCGGATATGGACTACTTGGAAACATATGTTGTTCATAGATAACTAT
AATTGGTACTCATAAAATTGTCAAGATAAGCGTGAGTGTCGTGGACGGCATTTCCGTATGGAGACGGAATGATTCCATGA
TGGAGTATTGTTGTGGTGTTAGTGGACTCGTGTGCGAGAAATCAAAGTTGTCTAAACTATTTGAAAATGCAACTTGTCTA
GCCATGAGTCAAATGTTGGCTTTCCGCATGAAACCCCACAATTCCTTATTGATACATTGCATGAGTAGATAGTTTATCAC
AAAGTCTTGCTGAGTACTTTTGTACTCACGTTTGCTTAATAAATTGTTGCAGAGGTTGTTAATCTATACTTCTATACTAA
TACTAATTAGAGACTCCCAAGAAATTCACACGTTGAGAGCCGTTTATCCGGTGGGCCTGAGTTATTATGGTGTAGATCTA
TTTGGAGACTCCAGATTTCTTTTTTTTGCAATGAAAGAATTTTTTTTACGTGATGATTTATGTTACTCTATTGCCAAACT
GCATGTAGACCTGACACATTGATTTGGTGGACTCAAATTATCACGGCTCACAATCATAACAACTTTTTTTATCTTGCCAT
GTTAATTAGGTCATGAATTTTCTATAAAAAAATAAAAGGTCATGTGTGTATCTACTGCA

BPB-6554

BPB-9447
TCGAGCGGCCGCCAGTGTGATGGATATCTGCAGAATTCGCCCTTGAGTAGTGCCAGAACGGTCCATGGGTTGTGCGCTTA
CAAAAAGTTCATTGATGCCTACTGTTGCCTTGCGAGAAATTTGTGTTATGCGACAGTCGATCCGGATACGCAGTCTACGA
AAGGGCGAATTCCAGCACACTGGCGGCAGTTACTAGTGGATCCGAGCTCGGTACCAAGCTTGGCGTAATCATGGTCATAG
CTGTTTCCTGTGTGAAATTGTTATCCGCTCAANNTAAACGAATGNNTTGTAGCTTGATTGNTTCCTCCACGCTNNTGCAC
AAGATANCCATCNCNCGATCGTCACTGATCCTCNNNGCTGCA

BPB-7829
TGCAGGGAGCATGATTTGTTCTTACTCTGTCCCACAAAGCGAGTTGATGGCCTGAGAATATTAACTATTCTTAGCTTCGT
TCAACTTTTCACCTTTTCTTCCAGTAGCACTTTTTACTTTAGTTAAATTTAAACATGTGTCTAATTTCAACAGAGAATTT
ATTTTACTTTAAATTTTTCTGTTGGTCAATACTGTCTCGGGCTATTTATGTGACCATAAAATGCACAAGAAATAAATATG
ATTCTTCACATACTAGAAAACACTTTGTGCTGTGTTATAGATCAGTTATCTAAAATAAACTCACCTGACCATGATCTTGG
GTTCTTGGGCAAGCAGCCAGTGACTTCTGCCGTTCCAGATGTTAATATCTAGATACAGTATCTCAAAACTTCAGTTCTGC
TGGTCGGTCATTCTTACCCAGTTATTTATCACATTAGCTACAATGATGGCCCACATTTCCATCTTTGAGAAAACTTCTCT
TAAAGATGGGGATTTGCTGCCTTTGACTTTTGATTTACTTCGTCCAGCTAGAATTAGTTGCAGCTCAGATTTGCTTATAG
TACGGCTGTCGATAGGGTCACTTTCTACATTCAACAGCCATACAGAGAGCCACAAGTAACCAAGTGATGCGAAGAAGGCA
AATGTTCCGGCGAGGCCTATATGTGACATGATGATCGGTGTTGCCAGGAAGCTTACGACGTTTCCAAGATGGAATCCTCC
CATGGAAAGGCCAACAGCAGTGGCACGTTCATGTGTTGGGAACCACCTGTTTCATGTTCNAAAATGAAATTAANAGCCTG
AACTGAAGTTAAGTTTGATTGCATCAATATATGTAGTGAATGNTTCATTTTCTGTAAGTAATTGGNNNNNNNNNNNNNNN
NNNNNNNNNNNNNNNNNNNNNNNNNNNNNNNNNNNCAAGGACCAGAGTGCAGCAGCACNNNCCATCACCTTCTTCCCCCC
GTATCGGTCCNCCAAAGCTCGTACAACCATGGATGAGAAAACATACCCCCATAGAAATGATGACTGCA

BPB-6071
TGCAGCTCAAGCCTCTAATGCGCAAGTAGTTCGGCAAGATGGCCAAGCCTGTCAAGAACTAGGTACTAACGCCACAAAAA
TTCTACTTGAAAAGTTGGAATGCACATGTTATGCTTGGTTTCTATGCTGCAACTCATTTGTTTGTGCACCTGCTCTTATA
GTCATTAAACTTTTTTATGGATTGTACTGTTAACATTTAAGAAGTTTTCCTGTCATTGGAGATTTTGTTTTGGTCTTTGT
TTTTAAATGGTGACCAAAGGTATATTGATGTAATTAGCCGTAACAACAAGGATATGAAGTGGTCAATTGGTTATTCAGTG
AAATGTTTTTTACGCTTGGCATCTATATTCAACCGGTTAAGAAAATTGTTTTTTATAAAATTAGATTCACGTTAGTGAGC
GTATATTAAATTATTTTTATCCGTTGCAACGCACGGGTATTTTTGCTAGTACACCTAAAAACGCCAAATAACATGGGCAC
GGCGGCAAACCCTATCTAGCGCCACCGGCACCGTTGAAACATAAAATGTGCTAACCGGCGCCTGCA

BPB-9873
CACTAGTAACGGCCGCCAGTGTGCTGGAATTCGCCCTTCGTAGACTGCGTATCCGGATCTATAACACAGCACAAAGTGTT
TTCTAGTATGTGAAGAATCATATTTATTTCTTGTGCATTTTATGGTCACATAAATAGCCCGAGACAGTATTGACCAACAG
AAAAATTTAAAGTAAAATAAATTCTCTGTTGAAATTAGACACATGTTTAAATTTAACTAAAGTAAAAAGTGCTACTGGAA
GAAAAGGTGAAAAGTTGAACGAAGCTAAGAATAGTTAATATTCTCAGGCCATCAACTCGCTTTGTGGGACAGAGTAAGAA
CAAATCATGCTCCCTGCACTGGATC

BPB-1009
TGCAGGGAGCATGATTTGTTCTTACTCTGTCCCACAAAGCGAGTTGATGGCCTGAGAATATTAACTATTCTTAGCTTCGT
TCAACTTTTCACCTTTTCTTCCAGTAGCACTTTTTACTTTAGTTAAATTTAAACATGTGTCTAATTTCAACAGAGAATTT
ATTTTACTTTAAATTTTTCTGTTGGTCAATACTGTCTCGGGCTATTTATGTGACCATAAAATGCACAAGAAATAAATATG
ATTCTTCACATACTAGAAAACACTTTGTGCTGTGTTATATATCAGTTATCTAAAATAAACTCACCTGACCATGATCTTGG
GTTCTTGGGCAAGCAGCCAGTGACTTCTGCCGTTCCAGATATTCAAATTGCAGTATAATTGTCCTACAGGTGCATTTCTC
CAATAAGTTTTTCCCAGCAGTTGCTGCA

BPB-8075
TGCAGAAGAAGGGAGACATGGCCAAGGACAAGTTCTCCAAGGTCCTTTGGGGCGCCGTTACCAACTACCGGACATGGATC
TTTGTCCTCCTCTATGGCTACTGCATGGGTGTCGAGCTCACCACTGACAATGTCATTGCCGAGTACTACTTCGATCACTT
CCACCTAGACCTCCGTGCCGCCGGTACCATCGCCGCTTGCTTCGGCATGGCCAACATCGTCGCACGTCCTATCTTGTGCA
CTTCCCGTCGCTCTTGTCATGGGCATCATGATCATGGCTTGTACACTTCCCGTCGCTCTTGTGCACTTCCCACAGTGGGG
CTCCATGTTCTTCCCAGCCAGTGTCGATGCCACGGAGGAAGAGTACTACGCCTCCGAATGGTCGGAGGAGGAGAAAAGCA
AGGGCCTCCATATCGCAGGTCAAAAGTTCGCCGAGAACTCCCGCTCGGAACGCGGTAGGCGCAACGTCATTCTTGCCACA
TCTGCCACACCACCCAACAACACGCCCCAGCACGTATAAGATCCAAATTTTCTTCTACCAACCAAATGTAAAGCTCGCAT
AAATATACACATCATATATGTCTACTCCTGCCAACCCCAGTTGTTCTGTACTTGTAGATGTTAGGAAACCTTCAACTTCA
TCTGTCTGCA

BPB-2751
TGCAGCTCAGAGCTCACCGGCATAATAGCTCAACCAATATTTGCGACACCGTACCCACGAAGGAATCTCACACACATGCA
TGCCTAGACTCGCCATTACGTCTACTTTCTTTCTTTATATAAACATAAAAAAGAGGTTTTCCCACTCGATTAATTATGTA
ATACTCCAGTGTTGGAGTACATTGTAGCACCACAAAATAGTAGTTAAGCTGCAAATCCGATGGTACAACCTCGTCGGAAC
ACACGATATATTAACCGGCAACAGCTGCGGCTGCAATGCCGCTGACGATATCGATCTTAGCTGCAACATTAAAGAGCACA
GTAGCTAGTACTCCATCGAAGTGCAGCAAGCAGGTAGGTAGGCGGTCGATCGAATCGACATGGACCTAATTAAGGGTTGT

AGTAGACGATCTTGATCTCGGTGACGCCGTGGCAGTTGCCGCCGGGGACGGAGTGCTCGAAGCAGTGGCCGCCGGTGTCG
CAGCCGGCGGTGGGGCAGTGGCCCTGCA

BPB-2628
CATGCTGGTAAAGTCCCAGAGAACTGATTATTGCTGAGATCAAGAAAGATGAGTCGTGTACATTTCTGCACTGGATCCGG
ATACGCAGTCTACGAAAGGGCGAATTCCAGCACACTGGCGGCCGTTACTAGTGGATCCGAGCTCGGTACCAAGCTTGGCG
TAATCATGGTCATAGCTGTTTCCTGTGTGAAATTG

BPB-4315
TGCAGCGACGTAGATGTAAGACACCCAGCTTGCTGCTGTGTGAATGTTGGAATATGCAATCTGGGATTGTCGCCAATTGG
TAATAGTAGTTATAATCTGGTAAGATGAAGCAGGATGTTGCCTCAGAAAGCACCATGCCAATATCATCAGGTAGCAGGCC
CTTCTTGTGCTCCACCAAAGCCAAGTGATCATGAAGCCTGCACTGCATGTGGCGACGAATGACATCATCATTACAGAAAT
AAAGCCATTTCGTAGAGATGTTTCTCTGTACAGCATCGGCTACTCCCCATGCCGACGTATCGCCTTGCCCTTGTATAATC
CCATCGCATACAAAGTAGTTAGAAACCCAGTTCTCCCAATTGAACATACTGCAATGTTTGAGTGCATCGGCATACTGGAT
GCACTTCTCAACTATTATATGGTTCATGTAAGGTTCTGGAATGCCGATATAAGCGGCAATCTCCTTGGACTCTTCCAATA
TTGCTTCATCGCGGATGCAATCATCATGAAAATAATTGACCATAACATCAGTGTGCCTCTCCATGTAATCAAAACCGGGT
CCAAACCTCCCGTGCGAGGTCCATAATACCTTAATACTCAAAAATGATATTACTGGGACTCCACACTCGTACAGATCAAT
GTATTCATCACTCCCATTGTGAAATATCACCACAAATCTACTGCTAGCAAGCTTTATGAAGATTTCTGTTCTGATATCTT
CTAGCACCCCTCGAGAGCCTTCGTCTATCCCACTGAAATCATCCTCCTCGTCCTGCTGATCGAAGATGGCCATCACCGAC
GGCGGAAGCTCCAGCTCCTCCGCGACTGCCTTCTGCA

BPB-7036
TGCAGCGACGTAGATGTAAGACACCCAGCTTGCTGCTGTGTGAATGTTGGAATATGCAATCTGGGATTGTCGCCAATTGG
TAATAGTAGTTATAATCTGGTAAGATGAAGCAGGATGTTGCCTCAGAAAGCACCATGCCAATATCATCAGGTAGCAGGCC
CTTCTTGTGCTCCACCAAAGCCAAGTGATCATGAAGCCTGCACTGCATGTGGCGACGAATGACATCATCATTACAGAAAT
AAAGCCATTTCGTAGAGATGTTTCTCTGTACAGCATCGGCTACTCCCCATGCCGACGTATCGCCTTGCCCTTGTATAATC
CCATCGCATACAAAGTAGTTAGAAACCCAGTTCTCCCAATTGAACATACTGCAATGTTTGAGTGCATCGGCATACTGGAT
GCACTTCTCAACTATTATATGGTTCATGTAAGGTTCTGGAATGCCGATATAAGCGGCAATCTCCTTGGACTCTTCCAATA
TTGCTTCATCGCGGATGCAATCATCATGAAAATAATTGACCATAACATCAGTGTGCCTCTCCATGTAATCAAAACCGGGT
CCAAACCTCCCGTGCGAGGTCCATAATACCTTAATACTCAAAAATGATATTACTGGGACTCCACACTCGTACAGATCAAT
GTATTCATCACTCCCATTGTGAAATATCACCACAAATCTACTGCTAGCAAGCTTTATGAAGATTTCTGTTCTGATATCTT
CTAGCACCCCTCGAGAGCCTTCGTCTATCCCACTGAAATCGTCCTCCTCGTCCTGCTGATCGAAGATGGCCATCACCGAC
GGCGGAAGCTCCAGCTCCTCCGCGACTGCCTTCTGCA

BPB-2768
TGCAGCATACTCAACAAAATGTATTACTTATTCAGTCTTCACAGGCCGGCTTTATACCAAAAGATACCACTACTTGTAGC
ATCTGTATGTGCATGGTGTGGCAATGTCTTTTGCTCAATGTCTTCGGTCACATCTATGGTGTTGCAATCTTTTTGCTAAA
CAACATGCCTATGCTTGTGCTTTCCCAGCTGTCGTGTTGCTACAAATTTTTGAAGGACGACTACNAGAAACTACACTCAA
TTGATGCAACCGCTCTTTATAGAATTGCGCTATCATCGGATCCCGATGCGGTTGTTAGCGTCAGTGGCAGTGATTCCGTG
TGTTCAGACGGCCCAGCAAACAACAGCCATAGCCAAATGACGTCAAGCGAAAACGAAAGCATATGCAGGATCGGTAGGTC
TTACTCCTAGTGCTGCTTAGGAGTAAAAAGTTCAGTACACATGTTTCATTTTCCCATAGCACACATACAATTACTATCAT
GTGTTTTTAACCTGCTGCTGCCCAGTAGTCCAATGCAAATTGCTGGTCTTAGCCTTCACAACTCAAAGTCAACTAAGTAA
CCGCTCAAACAGTTAAGGTGCTGCTTCTTTTTGGGAAGCATTGAAGCACAACTCTTAACAGAAATGTTAGCAACGGTCTG
CA

BPB-0742
TGCAGCCACTGTGTAATGTGATTACAGGCCCACATGTTGGCAGTTGGCACCTCCCATTAGTGGAAGAACTTGAAAAATAA
CTATGGCCCTTGATTACTCAAGCAAGAATAAATAAACGTGGCTTTTGATTTGCTACATTGATGTGTTTCGAGTAACTGTG
TACGGCTGAAGCCTCGCGCCGTTGTTGTTCTTCACTGCCCCAATATGTCTGCACCGCAGTGGCGGACCTAGAATCTCAGC
TTTGGGGATGCCACAATTCATAATTAACACTAATTATCATTGTAGTATCAATAAATAGTCTTAAAATAGCATCACTTTGA
TAACTATTTAGTGTTAAATACAAAAAGAAATATAGTGTACAATTTTATAGGGGGGGCCGCCCACTGGATCCGCCCCTGCT
GCACCGTATATGTAAACAAGCGATACTGGGATCTTGAATATGTCACCAATGTTCCAAGACTCAAAAGCAGTATATGTAGC
TAGCGCGGTAAAGTAGCTGAGAGCAAAATGTCCTGCTCCAAGCTATAGCATCTGTCGCTGCATTTAGATGATGAATTAGC
TCTGTTATTGAGGTCAGCAGGCAACGAGTCAGAAATTGATCTAGTTCCTGCA

BPB-3586
TGCAGACAGATGAAGTTGAAGGTTTCCTAACATCTACAAGTACAGAACAACTGGGGTTGGCAGGAGTAGACATATATGAT
GTGTATATTTATGCGAGCTTTACATTTGGTCGGTANAAGAAAATTTGGATCTTATACGTGCTGGGGCGTGTTGTTGGGTG
GTGTGGCAGATGTGGCAAGAATGACGTTGCGCCTACCGCGTTCCGAGCGGGAGTTCTCGGCGAACTTTTGACCTGCGATA

TGGAGGCCCTTGCTTTTCTCCTCCTCCGANNNNNNNNNNNNNNNNNNNNNNNNNNNNNNNNNNNNNNNNNNNNNNNNNG
AGGTCTAGGTGGAAGTGATCGAAGTAGTNCTCGGCAATGACATTGTCAGNGGTGAGCTCGACACCCATGCAGTAGCCATA
GAGGAGGACAAAGATCCATGTCCGGTAGTTGGTAACGACGCCCCAAAGGACCTTGGAGAACTTGTCCTTGGCCATGTCTC
CCTTCTTCTGCA

BPB-4246
TGCAGAAGGCAGTCGCGGANNAGCNGGAGCTNNCCGCCGTCGGTGATGGCCATCTTCGATCAGCAGGACGAGGAGGATGA
TCTCAGTGGGATAGACGAAGGCTCTCGAGGGGTGCTAGAANATATCAGNACAGAAATCTTCATAAAGCTTGCTAGCAGTA
GATTTGTGGTGATATTTCACAATGGGAGTGATGAATACATTGATCTGTACGAGTGTGGAGTCCCAGTAATATCATTTTTG
AGTATTAAGGTATTATGGACCTCGCACGGGAGGTTTGGACCCGGTTTTGATTACATGGAGAGGCACACTGATGTTATGGT
CAATTATTTTCATGATGATTGCATCCGCGATGAAGCAATATTGGAAGAGTCCAAGGAGATTGCCGCTTATATCGGCATTC
CAGAACCTTACATGAACCATATAATAGTTGAGAAGTGCATCCAGTATGCCGATGCACTCAAACATTGCAGTATGTTCAAT
TGGGAGAACTGGGTTTCTAACTACTTTGTATGCGATGGGATTATACAAGGGCAAGGCGATACGTCGGCATGGGGAGTAGC
CGATGCTGTACAGAGAAACATCTCTACGAAATGGCTTTATTTCTGTAATGATGATGTCATTCGTCGCCACATGCAGTGCA
GGCTTCATGATCACTTGGCTTTGGTGGAGCACAAGAAGGGCCTGCTACCTGATGATATTGGCATGGTGCTTTCTGAGGCA
ACATCCTGCTTCATCTTACCAGATTATAACTACTATTANCNATTGGCGACAATCCCAGATTGCATATTCCAACNTTCACA
CAGCAGCAAGCTGGGTGTCTTACATCTACGTCGCTGCA

BPB-5027
TGCAGAAGGCAGTCGCGGAGGAGCTGGAGCTTCCGCCGTCGGTGATGGCCATCTTCGATCAGCAGGACGAGGAGGATGAT
TTCAGTGGGATAGACGAAGGCTCTCGAGGGGTGCTAGAAGATATCAGAACAGAAATCTTCATAAAGCTTGCTAGCAGTAG
ATTTGTGGTGATATTTCACAATGGGAGTGATGAATACATTGATCTGTATGAGTGTGGAGTCCCAGTAATATCATTTTTGA
GTATTAAGGTATTATGGACCTCGCACGGGAGGTTTGGACCCGGTTTTGATTACATGGAGAGCCACACTGATGTTATGGTC
AATTATTTTCATGATGATTGCATCCGCGATGAAGCAATATTGGAA

BPB-2058
TGCAGCACAGACTCCGTCTGCGACCCGGCGGTGGCACCAACCTGTAAGACGACGGACCCCCTTACTTGCATTGCATTTGC
TTCGTTTATAGATGAATTTCTCATGACCATTGATGTCTATGTTCTCTGTTTTCTGTTGACATGACAAAAAGCACTATCTG
TTCTGATGATCTTGTGATCGTCTTGACGATTTTGCAGATGCACAAGGTGTTGCGGATTGCATAGCTGAGTGCAAGAAGAG
GTGGGGGCAATAATTAACGCCAAGTAACCTTAAAATAAACAGCTGGAGAATCTGTGGATCATGGGATCATATGATGCTGA
ACGTCTGTCACCGCTGAACCAATCAATAAAATGTGTTCCGCGTGGGTCGGTGCCACTTGTCTGATTTTTTTTATTTTTTT
TTTGCAAATCTCAGCTGCATATTTATTTCGTAAGAAGTTAACACATACTACAAGAATTAAATCATTAGCAATCCCGTCCA
AGCCGTCCGTGAATCCCAGTGAAAGCAAGCAACTCATAAGAACAAACACACAAGATGGGACGGCATACGCATAAATCTGA
TGCGGAATTCTCCCCGACAGATGCAACACAGAAAACAGTGTGACAGTATGAACAAACACAGCAAGATCAACCGAAAAGTA
CTGATTAATACAAGCAAGTCATTATGTCTTCAATCTTCATGTGAATTGGCAAATTTACTACAGCAAACTCATCCAAGCCT
TGCCTGATTGTTAATGAAACGAAACTAATTCGAAATTCATCAGTGAGAACCAGATAAACATTCACCAAGAATAAACGCAG
AAAATGGGGGCTAAATAGAAATCTGGTGCAGAATCTAAGAAAACAATGAAAAGATGAACACATACATAATTAAGGCAAGT
AAGATCAATTGAAAGGTACTAACTAATCGAAGCAAGCGAACAAGCCACTTCTGCATCTTGGCCACCGGAACGTAACTCTA
GATACCTGCA

BPB-9807
TGCAGCACCTCAATATAAGTGATCATTCGAGTATTGTCCTGTCTCCGATTGGAGGCGAGGGCCATGTCAACCAGCTGGTT
CCAGTTGAAGAACTCAGCATTTCTAAAAATGGTGCTAACGGGGAAGTATTGACTCAACTACTCTCTCATTTCCCGAAGCT
CACCATCCTGCATATAGCTTTTTGCATGAAGGTAGGAGAACTTGGTGTGATGGAGCAGCAAAGTGGATCCCAGAAGCAGC
AGACAAAAGAAGAGGAGGAAATAGTGGCGACGGCAGAAGGAGGGGTGCTGCTCTTGCCAACCCAACTACAGATCTTGACC
CTCCAACGTTGCCGTCGGGTGAGAATAGTACCCAGTTCAGGGGGCGACAAGAACGAATCAGCAGGAGGTCTCCAACGTCT
ACGCTCCCTCCGCATAGTGAGCGCAGCTTACTGCCCCGAGTTGCTTTCCTCCTATTCGGGCTCCTCGTCTGGTTTCCCTT
TCCCGACCTCCCTTGAAAGACTTAAACTATACCAAACTGAACAAGTCAATCTGGCCCTCACAAACCTATCCAACCTCCAA
GAACTCGAGCTTAGCTACATGGAGTCCCTCGAAAACCTAAGCATCAGCCGTTCCTCAGAACTGACCATAAATTACTCCTC
CGGAGTTCTGTCAGAGTCCATCTGCA

BPB-7995
CATGGCGAAGCATTTCTCTTTCCAGCTATCGCCGGCGTCGCTGCCGCTGCACTGCCCTCCGTTGATGTTCCTGAACGTGG
CCCCGAAATCGCCGTCCAGCAGCTCCTCGAGGCGCCTCATGCGGATACGCAGTCTACGAAAGGGCGAATTCCAGCACACT
GGCGGCCGTTACTAGTGGATCCGAGCTCGGTACCAAGCTTGGCGTAATCATGGTCATAGCTGTTTCCTGTGTGAAATTGT
TATCCGCTCAA

BPB-4917
TGCAGCTCAAATACATGTTCGTGTTCTTTCTGTTTGATATCCTGTTACAACTTCTTGTTCTTGCTTCTATCGTGGATATG
TTCAGTTCATATTTACTATTTTAAATGCTATTTGCATACCCTCTATTAGATTGCATGACTTGTCTTATCTTTGTTGTAAT
AGTCATGTTTTATCTATTATTTTTGTTAATTAAATCATATGATGAATTGCTGATATTTCCAACAATATATGTACACACCT
CGTCATTTCATTAGGAAGTGATCCCAGTCTCTAGTATTAAACTATTTTTTACTACATATAGCCATTTAATTATGTTGTGC
TTTTTAAAGAATCTATACATAACGTACTACTCCCTCTGTTTGGAAATACTTGTGGGAGCAATGGATATATATTTCTGAAC
AGGAAATACTTGTGGGAGCAATGGATATATATTTCTGGACAGAGGGAGTATATACGAAGTTAGGAACACAACTATCTCTA
TCCCGAAAGTTAATTTAATAGAAATTGTGTCTTGGTTCTTTGGATGCTGATACAACAAGAAGCAAAATTTGGAAGGAGAA
ACAGTAGAGCCTTAACTCTTATATAAAAAAGGCTTCTTATACTTCCTCTCTATTGAATAAGCGAATTGGTCATGAATCAG
AATAACACGCCCAAGAATGTACGAGAACCAAATACCAAAAAGTAAATAGGGAGAAAGCATAATTAGAAAATCCATGTTTG
TGAACTCGGTCTCAAAAGTAAAGACTTTAATGTGTTTCATGTACAGTTACTGCA

BPB-4843
GCTCGGATCCNCTAGTAACGGCCGCCAGTGTGCTGGAATTCGCCCTTCAGTCAAGTTAGATGGTGCAGTAACTGTACATG
AAACACATTAAAGTCTTTACTTTTGAGACCGAGTTCACAAACATGGATTTTTTAATTATGCTTTCTCCCTATTTACTTTT
ATGTATTTGGTTCTCGTACATTCTTGGGCGTGTTATTCTGATTCATGACCAATTCGCTTATTCAATAGAGAGGAAGTATA
AGAAGCCTTTTTTATATAAGAGTTAAGGCTCTACTGTTTCTCCTTCCAAATTTTGCTTCTTGTGGTATCAGCATCCAAAG
AACCAAGACACAATTTCTATTAAATTATCTTTCGGGATAGAGATAGTTGTGTTCCTAACTTCGTATATACTCCCTCTGTC
CAGAAATATATATCCATTGCTCCCACAAGTATTTCCTGTCCAGAAATATATATCCATTGCTCCCACAAGTATTCCCAAAC
AGAGGGAGTAGTACGTTATGTATAGATTATTTAAAAAGCACAACATAATTAAATGGCTATATGTAGTAAAAAAATAGTTT
AATACTAGAGACTGGGATCACTTCCTAATGAAATGACGAGGTGTGTGTACATATATTGTTGGAAATACCAACAATTCATC
ATATGATTTAATTAACAAAAATAATAGATAAAACATGACTATTATAACAAAGATAAGACAAGTCATGCAATCTAATAGAG
GGTATGCAAATAGCATTTAAAATAGTAAATATGAACTGAACATATCCACGATAGAAGCAAGAACAAGAAGTTGTACCAGG
ATATCAAACAGAAAGAACACGAACATGTATTTGAGCTGCACCATCTAACTTGACTGAAGGGCGAATTCTGCAGATATCCA
TCACACTGGCGGCCGCTCGAGCATGCATCTAGAGGGCCCAA

BPB-14536

BPB-8054
GCTCGAGCGGCCGCCAGTGTGATGGATATCTGCAGAATTCGCCCTTGATGGATCCANTGCAGTTAACAACCTGAATTTTC
TGATTCAGAATCCCCTTTCCTAGATGAGCATTTCTCTGCTGAGCGGATTTGAGGCGTTGGTGTTCATTTGACCTATAAAC
TTTGGGATCACCTTGTGGTTCTTGATTTCTGTCCATGTGCCATGTCAATGTCACCAGACTGACCGAACCTCGAGCACTCC
GCTGATCCTGATGTGTGCTTTACTGAAACAGAGACCCTTATATCGGATATCTGATTTTGCTTGTTTGCTTCTGAAACAGG
GAGCGCTTACTTTCAGCATTGTTTTACAACCATATGAAGCTGCCCTGACATTTTTCAGTTCGTTTTCTTTTGCAGGCTAG
TGCTTTCTTGGATGTTCTAACTGTGTGAGTTCGCATGAGACTGAAGACCAAAAATGTCTGCTGCA

BPB-4405

BPB-4565
TGCAGCAGCACACCAAATAGACAGCCCGGGCCATGATGAATATATAAACATCATAGCGTGTTAATTAAATGTAAACAACA
AGCAACATATATATAAGTGATCAACGGAGTATATAGAACTCTGAGACTTGATTTTCTATCTGTCCCGGCTTCTACCCATC
TCAGTGAGGTGTGAGACAACTAGTATGGAGGCCCCCACGACAGAGTGAATAGTGGACATATATGTTTGAAGTAAAAAAAA
GATGATACTCCCTCCGCCTTAAAATTATTGTCTTTGATTTGTCTAAATATAAATGTATCTATTCACAAAAAATGGTTGTG
AGGGCATTGTTGTTGTCGAATGTTGTGGTTCATCCAAGACTTTATCAGCAATGATACCTCGGGCTCTATATAGTGTATAT
ATGTGATACCTCGGGCTCTATATAGTGTATATATGTTTTTTTCTACGTTTGGCCTAGACTTTTTCAAAGATAATATAGGA
TTGACAGTAAAAAAAAAGTTCCCAAAGCAATACTCCAAAAAAGAAAAGAAAAATCCAACAATAACTGTTGTAGCTAGCAAG
AAAACTTGGGTTCCAAAAAGGGCCCAACCGGGTTCGAACCGGTGACCTATTGATCTGCACTGGNTCC

BPB-8283
TGCAGTCGAACCTCAGGAGTGTACTTTCAGGGACTTCTGTTATCGACAGCAGATATTGTGTGAATTCACGGTCACACAAT
ATTTTGGATTCCGAATGATCAAATTTTCTCGTGGGGGGGCGAGATTCTCGATTACCACGGTTACCGAGAAATACCGAATA
AATTTCGTACGAATTTATAATGGAATTTTGAATTCAAATTTTGAAAACTGTACAAAAGACTTGAACATGAATAGATATGA
AAAGTGTTAGTAGCTCAGTGGTATGTCCTGTACGTGCTCTCTAATTGCTGTAAGATCACGAGTCCGACTCTCATTTGGGT
AGTTTTCTTTTTTTTGTAATATAATGGAATTTTGAATTCAAATTTTGAAAACTGTACAAAAGACTTGAACATGAATAGAT
ATGAAAAGTGTTAGTAGCTCAGTGGTATGTCCTGTACGTGCTCTCTAATTGCTGTAAGATCACGAGTCCGACTCTCATTT
GGGTAGTTTTCTTTTTTTTGTACGTCTTGAAAAAAATAAAAAGTTACTGTCAAAGTGGTCGAACCTCGAACCTCTTGAGA
TCGGTGGTAAACGGTTACCACCACCCCACAGCTGCA

BPB-3068

CCAGTGTGATGGATATCTGCAGAATTCGCCCTTGATGNANNCAGTGCAGATGCAAGGCCTGTCTTAATTTTAACAGAAAT
GATCCTGTCTTAGTTTTAACAGAAATGATCCATGCTGCTTCTAGCTAGCTAGTAGTTGCCGTGACCTTTTGATGTTGCGT
CAGAGTGAGTGAGCAACTTGGACTGTAGTTAGTGACAACAACTATGGTTTTGCCTCTAATAGCTAACAGACACTCTCTAC
CATTACTTAGAAATGAGTGAGTTCGGGTTGTAATCAGTGACACCAACAATTAGGGTATTTGTCTGTGTTGTTTTTTTAGA
AACAAGAGGAAATTCATTTACTAATATGCAAACAAGAGAGTAAATTCATTTACCCAAGACCCAAGAGGCACGACTCTTTC
TGTGATATACTGATATTACTAGCAGAGATTCTCAAGTTTGATGCAGAATTTCTTCAATGTAGTTATTTTTACCAAGGTTC
TGCTCTTCAATTCCTGCA

BPB-9130
AGTGTGATGGATATCTGCAGAATTCGCCCTTGACCCAGTGCAGTATGTAGGTGCAGAACTGATTGCATGAATGGACCGCA
AAGAGATAAAACATGCCGCAAATTTGTTCTGAAGCACAAGAGTAAAACAAATTGCACTTCGAAATTGCGTTGTTCCGTTC
AACAAAAGGAAAATATGCATGGAACCATCAAAAGAAACTCAACAGGTGCCGCCGCAGTGAACAGCAAAAGCACTTATTTA
TCCAACATACTCCTACACAACCTTGGCTCAAATTGCGCACATCTATCATCAAAGATGCACAGGAAAGATAACAACGAATA
GTGGTTATATTCATGGAAGAAATTCTGATATGAATCAAGTTTTCTGTATCTATGCAGATATGAATGAAAGAATTCCAGAT
ATCAATCTGTTTGGAGGTCGACAAAAATTCTGACACCAAAGATCTCCATCCAATCTACTCTAAGATTATCTGTCTGTTGT
ATCATCCGCTGGCTCTCTTTGGCATGGATGTGCCTGAGCTTCACTCCAGACTCCAGAGTCTCGGGTGATAGTGTAGCTCC
TCTGCGCTACATCATGGTGATGTCTGTGAAATGAAACAGCAAATTAAATCCAATTAGTGACAATATCACAGCCATTAACT
TGATTAAGATTCGAAGGATGCAGGTACGGATAACAAGAACAATCAAAGGCTGCA

BPB-6607
TGCAGCCCCGAGGATACGGGAGCTCGAGCTCCTCCTCCGGCAGCCCCCCTCATCCGAGCAGCCACCAGCGCCGCCGCATC
CAACCTCCAAGGTCGGCCCGTCGGCCCTATCCTTGCCCTGACCTCTCTGCTTTCCCTCATCCTCACCTCGTTTCTCTCTC
TCTCTCTCCTAGACACAGAACGTCGTGGTCGTGTCGGCAGCACGCCTCCGCCCGAGCCAGCAGTTGCATCCTCTCTGTGC
ATCGCCCTCGCTGTTGTTCGCCTGCATCGGTCGCGCCAGCGACCTCAGCCGACGGAGCAGCGAAACCCTTCTTCCCTGCC
CTCTCTCCTTCTTATTCCTTCCCCAAAATTTCCCTCTCTGAAGACTCTGTTTGTCTTGTTTTCTTCAGCCAACAGCAGAA
TCGATGCCAACTCCTGCTGCCTTCCATCGGCGACGTCACGTGCCGATCTCGCCGGTGAGACCCCGCTCCCTCATGTCCCC
GTTTCTCCCCTTCTTCTCTCTCTAACCTCACATGCTCTCCCTATCTTTCTGCA

BPB-4125

BPB-6357
TGCAGGAGCGAACCAGCGCCGACCGAAGCGCTCGGTGGATGCGGACGGGGCGAGGTGAGCCATCGCGACCGCGGTAAGTG
CGGCGTGTTGGAGGCGACGATGCGATGACAACTTGAGGAGGAGCACCCGGAGGGCCGCGTAGCGGAGGCATGGAGTGGTG
CCAGCCAATGGTGCAGGTTCAGCGGCGGGCCAGCTTGGCGAGCGTGGCGAGCCAGTGGCGGCATAGGCGAGCTCGGAGAA
GCGTCGGCACAGGCGAGGCGCTGTGGAGGCGGAGTTGGCGGCTCGGACGCACGGCGTTGACTCCGTAATTGACCCGATAT
GTGGCCCCTTTTTATTTTTATTGCAGGAACTCTACCCCGGGACGGGAGTTGATATTTTGGGTGGATCATAATACTGCA

BPB-7786
TGCAGTTATCTATCTATATATCTGGTTATTACCACAACCAGAGACCTTTTTTTTTATGAGGCAAACCACAACCAGAGACC
TTGTACAGTGCATGACAGACTTTCACTACAAGAAGGCTTTTATTTTATTTAAATTCTCGGCCTATACCGCGGCCAAACTG
TGTCTGTCCTCCAAGTCTCCAGCCAGCCTCTCCCAGCCCTCATGGAGCTGCATCCTCCTCAGCGACATTGAACTCGCCGT
GATCGGCACCCCCCGGCAAGAGCAGCCATGCTGCCCTCAGTGACGTTGAACTCGCCGTGATCGGCACCCCCGGCAGGAAC
AGCCATGCTGCCCTCAGTGACGTTGAACTTGCCGTGATCGGCACCCTCTGCA

BPB-1933
TGCAGTGGGAATACTAGATGAAACCGTCAGGCCAAATCCAGCATGGTCTACAACGAGCAGGAAACCGTCGGGCCAAATCC
AGCAAGGTCTACAACGAGCAGGCCACCGAAGATAGGTCACGGGTCTGCTGAAGGATGACGAGAATCGCGCCAAGAACCGC
GGCCGCTGCCTTACACGGAACGTCGGTCCTGAGCCGATTGTGTACCGTCTGAGGTTTGGGAAATGCGGATCCCCCAGAAG
GATTTCGGAAGAAGGTTCCTTTTGTCAGCCCTGAACAGCCAGCCGATGTGGGACTCGCAGGCAGCACACAGCGCAACCGT
CCACGTATATCTGCA

BPB-1176
TGCAGGTGCTATTTTATAGCTGGAAACTTATTTTCGTATTCTTCATTCTAGTGAAGAAATGGATCAAAAGATGTTCATAC
AGGGCAACAAGCAAGAGAATTAAATCGATTAGTTCATTAGGATTCTGATGATTTTCTCTTTTCGGATGATAACTTGTTAA
AACATATCACAATTAGCTCAGTTAGTTCACTGTGATTATGATAATTTTACTAATTTCAAATGAAAACTTGCTAAAACATA
ACACAACTTTTTTTAAAGAGCTTTGGAATCTGTCATCGAGCACACGGATGACAATCTTTGAGCTCACAGCTCAAAGAAG

GACAAGCTAAACATGCATTGATTCTATGTTTCTGTTTTCAGACAGCAAGTTATTAAGAGATTAATAAAAAGAAGACATAA
AAATAATACTGAGTTTGCTGCCACTAGTAGTACCACAAACAGTAGCGAATGGCTGCGCCTCTGCA

BPB-7446
TGCAGCTCAAATACATGTTCGTGTTCTTTCTGTTTGATATCCTGTTACAACTTATTGTTCTTGCTTCTATCGTGGATATG
TTCAGTTCATATTTACTATTTTAAATGCTATTTGCATACCCTCTATTAGATTGCATGACTTGTCTTATCTTTGTTGTAAT
AGTCATGTTTTATCTATTATTTTTGTTAATTAAATCATATGATGAATTGCTGATATTTCCAACAATATATGTACACACCT
CGTCATTTCATTAGGAAGTGATCCCAGTCTCTAGTATTAAACTATTTTTTACTACATATAGCCATTTAATTATGTTGTGC
TTTTTAAAGAATCTATACATAACGTACTACTCCCTCTGTTTGGAAATACTTGTGGGAGCAATGGATATATATTTCTGGAC
AGGAAATACTTGTGGGAGCAATGGATATATATTTCTGGACAGAGGGAGTATATACGAAGTTAGGAACACAACTATCTCTA
TCCCGAAAGTTAATTTAATAGAAATTGTGTCTTGGTTCTTTGGATGCTGATACAACAAGAAGCAAAATTTGGAAGGAGAA
ACAGTAGAGCCTTAACTCTTATATAAAAAAAGGCTTCTTATACTTCCTCTCTATTGAATAAGCGAATTGGTCATGAATCA
GAATAACACGCCCAAGAATGTACGAGAACCAAATACCAAAAAGTAAATAGGGAGAAAGCATAATTAGAAAATCCATGTTT
GTGAACTCGGTCTCAAAAGTAAAGACTTTAATGTGTTTCATGTACAGTTACTGCA

BPB-7877
GTAAAACGACGNCCAGTGAATTGTAATACGACTCACTATAGNGCGAATTGNNCCCTCTAGATGCATGCTCGAGCGNCCGC
CAGTGTGANNNATATCTGCAGAATTCGCCCTTGANNNATCCAGTGCAGAGGCGCAGCCATTCGCTACTGTTTGTGGTACT
ACTAGTGGCAGCAAACTCAGTATTATTTTTATGTCTTCTTTTTATTAATCTCTTAATAACTTGCTGTCTGAAAACAGAAA
CATAGAATCAATGCATGTTTAGCTTGTCCTTCTTTGAGCTGTGAGCTCAAAGATTGTCATCCGTGTGCTCGATGACAGAT
TCCAAAGCTCTTTAAAAAAAAGTTGTGTTATGTTTTAGCAAGTTTTCATTTGAAATTAGTAAAATTATCATAATCACAGT
GAACTAACTGAGCTAATTGTGATATGTTTTAACAAGTTATCATCCGAAAAGAGAAAATCATCAGAATCCTAATGAACTAA
TCGATTTAATTCTCTTGCTTGTTGCCCTGTATGAACATCTTTTGATCCATTTCTTCACTAGAATGAAGAATACGAAAATA
AGTTTCCAGCTATAAAATAGCACCTGCACTGGATCNATCAAGGGCGAATTCNNGCACACTGGCGGCCGTTACTAGTGGAT
CNNAGCTCGGTACCAAGCTTGGCGTAATCATGGTCATAGCTGTTTCCTGTGTGAAATTGTTATNCGCTCACAATTNCACA
CAACATACGAGCCGGAAGCATAAAGTGTAAAGNCTGGGGTGCCTAATGAGTGAGCTAACTCACATTAATTGCGTTGCGCT
CACTGC

BPB-1758
TGCAGGTGCTATTTTATAGCTGGAAACTTATTTTCGTATTCTTCATTCTAGTGAAGAAATGGATCAAAAGATGTTCATAC
AGGGCAACAAGCAAGAGAATTAAATCGATTAGTTCATTAGGATTCTGATGATTTTCTCTTTTCGGATGATAACTTGTTAA
AACATATCACAATTAGCTCAGTTAGTTCACTGTGATTATGATAATTTTACTAATTTCAAATGAAAACTTGCTAAAACATA
ACACAACTTTTTTTTAAAGAGCTTTGGAATCTGTCATCGAGCACACGGATGACAATCTTTGAGCTCACAGCTCAAAGAAG
GACAAGCTAAACATGCATTGATTCTATGTTTCTGTTTTCAGACAGCAAGTTATTAAGAGATTAATAAAAAGAAGACATAA
AAATAATACTGAGTTTGCTGCCACTAGTAGTACCACAAACAGTAGCGAATGGCTGCGCCTCTGCA

BPB-5885
TGCAGCTGCTCCTTTCTTATCTTACTTTCACGAACAGGAAACAGTACCCTCTCTGCAACTCTGTCTGGTCCAAAACCTCC
ATGCTCCAAAGAAGCTGCCTGACCGGGTCCGGCTGCGAGGCCAGACTACCTTTCTGCCCGGAGCCAGCCTCGGTGCCCGT
GCCCTCCGACTGTAGCGTCCCATCGAACGCCGCGAACCACCCGACCACAACAAGGGCAGAGCTTTCTCCAATCAATCAGG
GTCAAGTTACTACTAGCATATAATAGTACAATGCGAACATGCGCATTCTTCAGTTCAACACGGACGAACAATTCCCTTCT
CTTTGCCAGATTTGATTAGTTCCTTTTTCATGATATAATGATCGATAAATGAAGTCATTGTGCTCCAACTTTGTAATAAG
ATGTTCATTAAACTGCACTGCTCTCCGTAAAGAGCAGAAACCAACCACTGTTGTAAACTGAAAAGAACGGACACCTGCAC
TTTCTTGTGGCTAGGATTGGACTTTGAACTTTTCAGTTATGGATCGGAGTGGGTATGGGCAGCTCTCAGACCATTCCCAG
CTTGAAATATATAGTGCTAAGCTCCCCTGCA

BPB-5515
TGCAGCAAAGAAGACAAAAGGTTTCAAGCCGTCTCCAATCTCATGCTGTACAAACTCGTTTATTTTTACCATAGCGAATT
TAGCGCTTTTGACTTCGATGCTTTTCGCTGCTGCTGCTACTACTACTTACGAACTGCGGATTGCAACATGCAGCAGGTTC
TTCTCCGAAGCCGCTCAGTTCTGTCGCGTCAATGAGTTGGATATCCGGGCCAAGATACAGAGGAAGATGCTCTCGCAAAA
CACAACGTATGTCGTATGCTTGGTGTTCAAGCTAGCATATGTATACTCTGGTCCGATTTCACGCACGAGGTGGCATCCGT
TGGCGTTGCCGGGAGGGAGTCGACCCGGCAAGTTTGCGTGCAAGGCTACGTCGACGATGTGGATGGGGCTGGCAATCCTC
CCGGAGAGGAAGTTCATTTCCCTCGTGAGAAAGCCGACGGCTGGATGGAGGTGGAGTTGGGTGAGTTCCATAACGAGGAG
GATGACGATGGTGGCGAGGTGTCCATCAGCTTCACGGGAGAAAGCAAGTCTGGTCTTACCGTGCTGGGCATTGAGCTCAG
AAGTAAGCAACAAAAGCCAGCATGAATGTAGCATAAAGTAACGCTTTGGGGGTAAAAATATTATTACTATTTGAAATGTC
TTTGGTTACATGCATGCATGTGAAGAGGCTAAGAATTTAAGTTACAGTAGTGATTTTTTTTAATATAAGGGGTTTCCCCC
CTGCTCCATTTTATTGATGAAGCAAAGACCACCAGTTTTACAACTCTCACACAAACAAACAAGGACCAACACAGATCGAA
AGTATCTCAAAACAAGAATTTAGCCAAGTCACCACCTCTAAACAGAAAAACAAGCTAACTTTTAACAGAAACATCAGCAC

ATGTAGCATTCAGCTCCTGCA

BPB-0649

BPB-1029
TGCAGCTGTCAGAAGAGGGTAGTAGGGCCACGCTGTCGAACACAATCAAAGCATGCAGTCAAAATTATGTTAAAAACAAT
AAATACTCTTCATAGAACATGACATTCTGATAATGAGCACCAACCAAGTTCAGCATTGTAGCCACTGAGTACCAAGCTGC
CAATCGATGCAGCACCAAAGGCACTGATCCATTGCTTGGTCGAATCCCCAAACCTTAATGCTGTGGACTTAACACCTACT
TTGAGGTCATCTTCTTTGTCCTGACATGTATTGGGATACCACCATAATGAAAAACGATAATCCTGATAAGATGCTTTTCC
TCAGTTTAGATATGAAAGAACACCACTAGTAAAATACCTGATGTGCATATATGGTATCGTACACCAATGTCCAACATATA
CCGGCAGTATATAGTGGAAGGATGACTGCA

BPB-8382
TGCAGGTATATTTATATCCAGATCAGTTTCATTTTGATCACAAACCATCTTGTATAGCTCTGCAAGCAAACATATGTCAG
TTGTTAACGCCAAAAACACGTGTTGAAATATAAGGCATGAGCCAAAAGTGTGCTCTTCTAAATACGCAGACATGCACCAC
CAAATGTGTACACACAACTATGAAAAACAATTGTCCAATAAATAATCAAAAGGCTTAAGTAAGGTTGTGATGGTCATTCT
AGTGGTAAAAAATGGTAATATACGTTGTCAGGCAACTTGTCTTGTTGATCTCATTTAACTGTATCCCAACATCTTATCTT
CTAATTAAATACCAGTTGAGAAAGGTCATACATGGAAAACATTGCCTGTTGAATTTTATGAGACAGATCATATAGCACAT
ACCATGCCGATTATTCAGGATTACAATAGCAGAAGCACCAGCATCTTCAGCAACCTTAGCTTTTGTAGTGAACTTACAAT
CTCCTCTTTGCACTAGCAAAACTTCTCCAGCAACCTAAATAAAAGAACAATTAATCACTACTTCCTCTGTACCTAAATAA
ATGTAGTTGGGGAGAACTAGTCTAGTTCTCCCCAACTACAATTATTTAGGCACAGATGGAATACATAACAATGTAGGATA
CTATAAAAATTTCGCAAACAGCTAAATCATGTTTTCCCAGAAAGGTAACCTTTTCTTTGAGAGGGGTACAACAATCAAAA
GGGTCTGCTAATAGTAGTCCTGTCCGGTTCGCGTGCTTTTCCTTTGACTCTATTATGGGGCCAAACCGAGCACCAACACC
AACAAACTCATCGGTCTCTCTGTTTTTGACCCAAGTTCGCACCTTTACCTGATAAAAAGGTGAGGAAACAAAATTTGAGA
ATGAGTACATAGTCCATTAAAAAGAAGTAAGTAGATGCTGAAGTACAAAATCTCCGCCGGAGAAAAAAGGAAGAGCTGCA

BPB-6875
TGCAGCTCTTCCTTTTTTCTCCGGCGGAGATTTTGTACTTCAGCATCTACTTACTTCTTTTTAATGGACTATGTACTCAT
TCTCAAATTTTGTTTCCTCACCTTTTTATCAGGTAAAGGTGCAAACTTGGGTCAAAAACAGAGAGACCGATGAGTTTGTT
GGTGTTGGTGCTCGGTTTGGCCCCATAATAGAGTCAAAGGAAAAGCACGCGAACCGGACAGGACTACTATTAGCAGACCC
TTTTGATTGTTGTACCCCTCTCAAAGAAAAGGTTACCTTTCTGGGAAAACATGATTTAGCTGTTTGCGAAATTTTTATAG
TATCCTACATTGTTATGTATTCCATCTGTGCCTAAATANNTGTAGTTGGGGAGAACTAGACTAGTTCTCCCCAACTACAT
TTATTTAGGTACAGAGGAAGTAGTGATTAATTGTTCTTTTATTTAGGTTGCTGGAGAAGTTTTGCTAGNGCAAAGAGGAG
ATTGTAAGTTCACTACANAAGCTAAGGTTGCTGAAGANGNNGGNGCTTCTGCTATTGTAATCCTG

BPB-7644
TGCAGTTTTCAGCATCCACATGATCACACCGAGAATCTTCCTGTCAACAAGATACAGTACACTCTGGGGTGGTTTTAGAT
CGTGACAAGGATGACAATGTCGATCACTGGATAGTATCTTGCAAATTCAGCAAAATCTCCGCTGGTAAAATGAGGAGGGA
GGATTTTACATATTAACCATCTGATATAATACAGTACTATGAGGACCGGGCGCTTTGCTATGCCCTCTGATTAAAAAAGA
CTAATCACTCTTTTAAAATACAACATGTATTACTTTGTTAAAATGGCAAACCTTTTAAATTTAATCAAATTTGTAGATAA
ATATGTCAAAATTAATAATATCAAATTCTCGTGATTAGATTCATCGTTAAATGAATTATCATACTTTTTTATTTAATATT
ATGGATGTCGATATTTTTACTCTCAACTTGGTCAAAGTTGAAGAACGTTGAAAAGATGTTGTAGAGCTCAAGCTAGCTGC
A

BPB-0443
TGCAGCTAGCTTGAGCTCTACAACATCTTTTCAACGTTCTTCAACTTTGACCAAGTTGAGAGTAAAAATATCGACATCCA
TAATATTAAATAAAAAAGTATGATAATTCATTTAACGATGAATCTAATCACGAGAATTTGATATTATTAATTTTGACATA
TTTATCTACAAATTTGATTAAATTTAAAAGGTTTGCCATTTTAACAAAGTAATACATGTTGTATTTTAAAAGAGTGATTA
GTCTTTTTTAATCAGAGGGCATAGCAAAGCGCCCGGTCCTTATAGTACTGTATTATATCAGATGGTTAATATGTAAAATC
CTTCCTCCTCATTTTACCAGCGGAGATTTTGCTGAATTTGCAAGATACTATCCAGTGATCGACATTGTCATCCTTGTCAC
GATCTAAAACCACCCCAGAGTGTACTGTATCTTGTTGACAGGAAGATTCTCGGTGTGATCATGTGGATGCTGAAAACTGC
A

BPB-6677
TGCAGATCAATAGGTCACCGGTTCGAACCCGGTTGGGCCCTTTTTTAAACCCAATTTTTCTTGCTAGCTACAACAGTTAT
TGTTGGATTTTTCTTTTCTTTTTTGGAGTATTGTTTTGGGAACTTTTTTTTACTGTCAATCCTATATTATCTTTGAAAAA
GTCTAGGCCAAACGTAAAAAAAAACATATATACACTATATAAAGCCCGAGGTATCACATATATACACTATATAGAGCCCG

AGGTATCATTGCTGATAAAGTCTTGGATGAACCACAACATTCGACAACAACAATCCGGTTTGAAAGACCATTTTTTGTGA
CTCCTCCAAATGCCGTCACAACACCATACTGTCAATCCTATATTATCTTTGAAAAAAGTCTAGGCCAAACGTAGAAAAAA
AATATATACACTATATAAAGCCCGAGGTATCACATATATACACTATATAGAGCCCGAGGTATCATTGCTGATAAAGTCTT
GGATGAACCACAACATTCGACAACAACAATCCGGTTCGAAAGACCATTTTTTGTGACTCGTCCAAATGCCGTCACAACAC
CATTTTTTGTGACTCGTCCAAATGCCCTCACAACCATTTTTTGTGACTAGATACATTTATATTTAGACAAATCAAAGATA
ATAATTTTAAGGTGGAGGAAGTATCATCTTTTTTTTACTTCAAACATATATGTCCACTATTCACTCTGTCGTGGGGGCCT
CCATACTAGTTGTCTCACACCTCACTGAGATGGGTAGAAGCCGGGACAGATAGAAAATCAAGTCTCAGAGTTCTATATAC
TCCGTTGATCACTTATATATATGTTGCTTGTTGTTTACATTTAATTAACACGCTATGATGTTTATATATTCATCATGGCC
CGGGCTGTCTATTTGGTGTGCTGCTGCA

BPB-2940

BPB-2863
TGCAGCAGCACACCAAATAGACAGCCCGGGCCATGATGAATATATAAACATCATAGCGTGTTAATTAAATGTAAACAACA
AGCAACATATATATAAGTGATCAACGGAGTATATAGAACTCTGAGACTTGATTTTCTATCTGCCCCGGCTTCTACCCATC
TCAGTGAGGTGTGAGACAACTAGTATGGAGGCCCCCACGACAGAGTGAATAGTGGACATATATGTTTGAAGTAAAAAAAA
GATGATACTCCCTCCGCCTTAAAATTATTGTCTTTGATTTGTCTAAATATAAATGTATCTATTCACAAAAAATGGTTGTG
AGGGCATTGTTGTTGTCGAATGTTGTGGTTCATCCAAGACTTTATCAGCAATGATACCTCGGGCTCTATATAGTGTATAT
ATGTGATACCTCGGGCTCTATATAGTGTATATATGTTTTTTCTACGTTTGGCCTAGACTTTTTCAAAGATAATATAGGA
TTGACAGTAAAAAAAAGTTCCCAAAGCAATACTCCAAAAAAGAAAAGAAAAATCCAACAATAACTGTTGTAGCTAGCAAG
AAAACTTGGGTTCCAAAAAGGGCCCAACCGGGTTCGAACCGGTGACCTATTGATCTGCA

BPB-4626
TGCAGTGTCCTTCTTCAGAGGTGGCCAAACGGGCCAGCCCGACAAGGCACGATACACGTTAATCGTGCTTGGCATGAACC
GATACATGAGGTTACGGTTAGTAAACGGGTTATGCTATACCGCCCCGTGTGGTGCGCCTGTTAGCCTAGGCACGAGCCCT
TGGCTAATCGGACCGCCAGTAGGCACGGCTGGGCTGCATATTCTCGGTATGCTAAGCCTTTTAGTGTGTGACAGCTGAAA
AATCCTATCTGACGCCCTCGGGCGTTGAAAGGATGTGGCTTCGCTGAAGCGTGGCCACCCGACGCCACAGTTTGGGCCGG
GCCATGGTCGCTCTGCCTTTTCTTCTTTTGTTCAAGATATATTTTGAAAAGTATGTTGAATGTGCATTTAAATATCGTTC
AGCATATATGTGTACTTAAATATTGCGAGTTCGAGTCGGTTCGCCCGCCTAGCGCTCTTCGGCGAATGGCCTACCTTGCC
ATTTAGCCGGTGCCGTGTGTTGAGTGGGTGCTAACGGGCCAGCCCGCCGGTTTGGCCTTCTTCTTCCTCCTCCTCCTCCT
TCTTTTGGATCTGCTATATATAAAAAAACTGAGGCATAGGAGCAGAGGATTGCAGTGCACGTGCCAGCATTCAATGGACC
TCACACCCACACCCAGACGGTCACGGGCACACACACATGATGGAATGGAAGGAACTGCGCTACGTAGATTTGGATTTGGA
TTTGGATTTCAATTTAGATTTGGATATAGGGCGCGTACGAATTGCATTCCTTCATTGTTTGATTTGATTTGATTAAGGCA
AGCAAGCAAGCATCTCTCCCGTCACTACTTACTGTACTTACTTACTACTAGTATAATGTTAGAAACGAGATTAAGCGTGG
TATGGATGGATGAACCATGAACCAACACTTGGCTTGGCTGCA

BPB-9817
TGCAGGTACAAGAGATTGGAGGTCCTCGCCGCCTTCACCAATGCCGTAAGCGTCGCAACCTGCCTTTTAACATCCTTTGT
TTGCATATATACAATTTTACGGTGGCAAATCATGTATGTACAGAAGCAGCAATAACCAAGTAGCAGTGCCGCCAAGTCCA
CCTTACATGTGTCAAACACAACTGTCCCCTGTTTTCTCCACGATACCGATGTCGAATAGAAACAAGACAGTGCAAGCACC
ATGCTATGATAAAAACATATGCTTCAATTGCAAGGCAGCACAAACCAACACTTGTTTTTATCTTCTTATGTATGATTTTC
TATGTGCCAAACTAAGCTAGCAACAACAGCTAGATTTTCTTGTAGCACAAAACTGGAACAGTCTGTTGATGCCTTTACAT
TACATACCAAGTGACCTTTTGGATCATTTGTTTAATTTCTTTATACTTGGTGGCACACTTTCTGGCCTTTCTATCTTTTT
TTGTCCTAACGTTTTGACCACCCTCCATATGGTTGTTTATGTAGACAGCTGTTCCTGCTGTTCCTGTCTTTCTCCTTGGC
TGTTGAAGCGCTGCATTCATTTATGCAGGATGAATCCGAGCACAAGTAAGCGCCATTTATGTATGCTCACAGATGGCTCT
GTTCAAATAATTCTTTTCTATGTTGGTTGTTGAATGTGTTTACCTTAGGAGGAAACAAAGGAGATCGGATGTGAAAAATC
CAGTCACTGTAATGCGTTATGTTGCATCGTACTAGTATGATAAGTATGTGTTGGTTCAGTCCATTGTGGGTAACTAAATT
GTCTTCTCAGAGGAAACTGCTTAAAAAGAAAGATAATTGCATCTACAGTTTGTAGTCAGTGTTACACCGCAATTGATCAT
CTGCTGTCCAACCTCTGTGACATTATTTTCCTGAGAAACCGAGAATTGTTTGGTCAACTTTTATTTCCATGCATCTACTG
CCTCTTTTCATCAGCCATGGCCTATCAATCTAAACTCTTTGCTAGCCTAATATTTTCCTTGCTCTTAAAAAATTATCTTT
CAGGCATTACCTCATTGTTTCTGCA

BPB-9890
TGCAGGTACAAGAGATTGGAGGTCCTCGCCGCCTTCACCAATGCCGTAAGCGTCGCAACCTGCCTTTTAACATCCTTTGT
TTGCATATATACAATTTTACGGTGGCAAATCATGTAAGTACAGAAGCAGCAATAACCAAGTAGCAGTGCCGCCAAGTCCA
CCTTACATGTGTCAAACACAACTGTCCCCTGTTTTCTCCACGATACCGATGTCGAATAGAAACAAGACAGTGCAAGCACC
ATGCTATGATAAAAACATATGCTTCAATTGCAAGGCAGCACAAACCAACACTTGTTTTTATCTTCTTATGTATGATTTTC
TATGTGCCAAACTAAGCTAGCAACAACAGCTAGATTTTCTTGTAGCACAGAACTGGAACAGTCTGTTGATGCCTTTACAT
TACATACCAAGTGACCTTTTGGATCATTTGTTTAATTTCTTTATACTTGGTGGCACACTTTCTGGCCTTTCTATCTTTTT

TTGTCCTAACGTTTTGACCACCCTCCATATGGTTGTTTATGTAGACAGCTGTTCCTGCTGTTCCTGTCTTTCTCCTTGGC
TGTTGAAGCGCTGCATTCATTTATGCAGGATGAATCCGAGCACAAGTAAGCGCCATTTATGTATGCTCACAGATGGCTCT
GTTCAAATAATTCTTTTCTATGTTGGTTGTTGAATGTGTTTACCTTAGGAGGAAACAAAGGAGATCGGATGTGAAAAATC
CAGTCACTGTAATGCGTTATGTTGCATCGTACTAGTATGATAAGTATGTGTTGGTTCAGTCCATTGTGGGTAACTAAATT
GTCTTCTCAGAGGAAACTGCTTAAAAGAAAGATAATTGCATCTACAGTTTGTAGTCAGTGTTACACCGCAATTGATCAT
CTGCTGTCCAACCTCTGTGACATTATTTTCCTGAGAAACCGAGAATTGTTTGGTCAACTTTTATTTCCATGCATCTACTG
CCTCTTTTCATCAGCCATGGCCTATCAATCTAAACTCTTTGCTAGCCTAATATTTTCCTTGCTCTTAAAAAATATCTTTC
AGGCATTACCTCATTGTTTCTGCA

BPB-2410
TGCAGGTACAAGAGATTGGAGGTCCTCGCCGCCTTCACCAATGCCGTAAGCGTCGCAACCTGCCTTTTAACATCCTTTGT
TTGCATATATACAATTTTACGGTGGCAAATCATGTATGTACAGAAGCAGCAATAACCAAGTAGCAGTGCCGCCAAGTCCA
CCTTACATGTGTCAAACACAACTGTCCCCTGTTTTCTCCACGATACCGATGTCGAATAGAAACAAGACAGTGCAAGCACC
ATGCTATGATAAAAACATATGCTTCAATTGCAAGGCAGCACAAACCAACACTTGTTTTTATCTTCTTATGTATGATTTTC
TATGTGCCAAACTAAGCTAGCAACAACAGCTAGATTTTCTTGTAGCACAAAACTGGAACAGTCTGTTGATGCCTTTACAT
TACATACCAAGTGACCTTTTGGATCATTTGTTTAATTTCTTTATACTTGGTGGCACACTTTCTGGCCTTTCTATCTTTTT
TTGTCCTAACGTTTTGACCACCCTCCATATGGTTGTTTATGTAGACAGCTGTTCCTGCTGTTCCTGTCTTTCTCCTTGGC
TGTTGAAGCGCTGCATTCATTTATGCAGGATGAATCCGAGCACAAGTAAGCGCCATTTATGTATGCTCACAGATGGCTCT
GTTCAAATAATTCTTTTCTATGTTGGTTGTTGAATGTGTTTACCTTAGGAGGAAACAAAGGAGATCGGATGTGAAAAATC
CAGTCACTGTAATGCGTTATGTTGCATCGTACTAGTATGATAAGTATGTGTTGGTTCAGTCCATTGTGGGTAACTAAATT
GTCTTCTCAGAGGAAACTGCTTAAAAGGAAAGATAATTGCATCTACAGTTTGTAGTCAGTGTTACACCGCAATTGATCAT
CTGCTGTCCAACCTCTGTGACATTATTTTCCTGAGAAACCGAGAATTGTTTGGTCAACTTTTATTTCCATGCATCTACTG
CCTCTTTTCATCAGCCATGGCCTATCAATCTAAACTCTTTGCTAGCCTAATATTTTCCTTGCTCTTAAAAAATTATCTTT
CAGGCATTACCTCATTGTTTCTGCA

CHR. 7H

BPB-0427
GGANCAAAAATGTAATGTGGAGCTCGAACCGGGTTGAGAATACGAAATATTCACACATACTCCGTGTGCCGTTCAAGACA
GAAGACGGAAAGGATTTGCGCCAGTGGGTGTCCCGGTTTGACATTTACCCTTACCTAGAGAGATACACTCAGGTTTGCCA
GCTGCGAATTATTAACAATCAGTACTCTTGTGTGAGTGATCTTGAGTAAAAAATTATCCCGATATGATTTCAAGATGCTT
CTGCCAAGATCCTTGACATTCTAGAGGGCAAACCAGACTTGATCATTGGCAACTACACTGACGGAAACTTGGTGGCGTCC
CTCATGTCAAGCAAGCTAGGAGTCACACAGGTTAAAAAAATGTCTTCTAATCAAATAAAAGCCCTCAAGATTTCTTTTAC
CACCAATCACAGTTGACATGACATCCCTAAGTGGTCTTCCTGACAGGGAACAATTGCACATGCTCTCGAGAAGACGAAGT
ATGAGAACTCAGATGCTAAGTGGAGAGAGCTGGACCAAAAATACCACTTCTCCTGCCAATTCACTGCACTGGATCCNTCN
AGGGNCNAATTCTGCANANNTCCNTCNNNNTTGCNGGCNGCTCGAGCATGCATCTAGAGGGCCCAATTCGCCCTATAGTG
AGTC

BPB-0108
TGCAGGTCGGAATCTATGAAAGCCTTCATAGCACGGAGGACGACGAAGTGGATTGGAAGTGGAGGAGAGCCGACGAGGAA
ACTGAGGGAGTCTCCCTTGTTTACACGTTACACGTTACACGTTTGGGAACACTAATTCACATGGGCACTTTGATCATTTG
CAAATTTACATTACAATGATGTATTGTGAATATGCCGAAGATAGGCGTGGGTAGGGAAACTGAATCAGAAGGACGGCCAT
ACATATGGAGCAAAATGAATGAATCTACACTCTAAAATATGTCTATATACATCCGTGTGTAGTTCGTAGTAGAATCTTTT
AAAAGACTTATATTTAGGAACGGAGAGAGTAGATAGCAAGCTTTATATATGTAGCTGACAAGCTCATGTAGAATTCAGTA
CGGTACGAATAGATATCAAGCTTTATATGTATCCAGCTGGAAGCACGCGTCCGTCCAACTGTCACCTCAGCCTCGCCCGG
CTACCTCTATTCCTCTCTGGCATAGCTGATTGTAAGCCCCTCCTCTGCTCCCTCTGATGGTAAACTCTGAACTTGGTTGC
TTGCTTACTTTCTGCA

BPB-9729

BPB-8921
TGCAGCAGCTTCACAATATTTCGATTTGGACTGGATAAATGTCTCTTTCTCATCAAAAACAATGGCGAAAGGTTTCAATA
AAAACAGTTCTTCGCGAGAACGGTCGTGCTGCACTTCACCATCGGCGGCAACCTCCATCCTTGCGGAATCGGCAAAGGCA
GAGGAACACGAAGCATGTATGCCACCGTCAGATCTGTAGAGACCATGAATGGCAATCAGACTTGTAAACGGGAATTCTGT
CCACAGCCTTGTACTAACGCCCTAACAATCAAGATCAAATAGCTTAACTGACAAATCGACTGAGCCTCATGTGTGCCCTG
TTTTCCTGCATAAATAAATAAATAAATACTGAAATCGTTTAACTGACACTGTTACCCAATCTGAAGCCACGAAAGAAGTG

GATCAGAATCGGCGGATGATAACATCGGATGTACCTGCA

BPB-2216
TGCAGTGGTGTTTTTCAAATCTGCTAGAATCATCAATGGCTGTCTTTTTCAAAAAAGGACAGTAACGATTTCTCCTGCGC
TCGCTAATCCGCCTATCTGCGTGCGTTGCATGCAGCCTGTTAGGAGCTCCTAATTAGATGTATGCAAAAAAATGATGTCA
GTGGATTCTTTTTAAAAATTGAAAATTAAAAATGTTTTGTAGCTTAAACTATCCACTCGATTGAAAAACTGTTTTCACAT
AAAAGATTCTTCATCACGAGACCTTCAAAATTATATCGCGTTTTGATAAATTTCAACGACATTTTAAAAAGATTATAACA
TCTAAACTACGTAACCTATCACACCTATAACACATAAATTATCATGGTTTTTACAGTGAGGCTATTGGGTATATTTTAAC
CTTTTTTGTCTAAGTCAAAATATACCACATGATTCGTAAGTAAATTATTAGGTCCGCAATTCCACCGTCCATCATCGATG
TCATGCCTAGCCGCAAGGTGAGGTTCACATGCCGATGTTGTATGGCCCGCTCATAAACATGACTTTGTGAGTCGTGCTTT
GCTAGGTTCGTTTAGGCATGTGCCGGGTTGTGATTATTGTACATGCATTATCTGCA

BPB-2875

BPB-0043
TGCAGTGGTGTTTTTCAAATCTGCTAGAATCATCAATGGCTGTCTTTTTCAAAAAAGGACAGTAACGATTTCTCCTGCGC
TCGCTAATCCGCCTATCTGCGTGCGTTGCATGCAGCCTGTTAGGAGCTCCTAATTAGATGTATGCAAAAAAATGATGTCA
GTGGATTCTTTTTAAAAATTGAAAATTAAAAATGTTTTGTAGCTTAAACTATCCACTCGATTGAAAAACTGTTTTCACAT
AAAAGATTCTTCATCACGAGACCTTCAAAATTATATCGCGTTTTGATAAATTTCAACGACATTTTAAAAAGATTATAACA
TCTAAACTACGTAACCTATCACACCTATAACACATAAATTATCATGGTTTTTACAGTGAGGCTATTGGGTATATTTTAAC
CTTTTTTGTCTAAGTCAAAATATACCACATGATTCGTAAGTAAATTATTAGGTCCGCAATTCCACCGTCCATCATCGATG
TCATGCCTAGCCGCAAGGTGAGGTTCACATGCCGATGTTGTATGGCCCGCTCATAAACATGACTTTGTGAGTCGTGCTTT
GCTAGGTTCGTTTAGGCATGTGCCGGGTTGTGATTATTGTACATGCATTATCTGCACTGNNNTCCATCAAGGGCGAATTC
CAGCACACTGGCGGCCGTTACTA

BPB-5259
TGCAGCATCTTGATCTCTCATGTCATTTTTACAGAGAAGGAATGTACTCAAGAGACATTTCATGGGTAGCGCGCATTCCT
AATTTGCAGTCTCTTGGAATGAATGGGGTAAACCTCAGCACGGTAGTTGATTGGCCTTATGTTATCAATATGATCCCTTC
CTTGAAGGCCCTCAGTCTCCAGTCTTGCTCTCTTCCAACCGCAAATCAATCACTCCCACATATTAATAACCTTACCGAAC
TGGAGAGGCTTGATCTCTCTGGCAACATCTTTGCCCACCCAATGTCAAGGGGTTGGTTTTGGAATTTGACAGGCCTCCAG
CATCTTTACCTTGCCGGCACTCTACTGTACGGTCAAGCACCTGATGCACTGGCACGTATGACGTCCCTTCAAGTCCTTGA
TTTGTCAGGTAATCGTGACATGGGGATGATGAGTAGTACAAGCTTAAAGCACCTATGCAGTCTGAAAATTCTGGACCTTT
CTTTTTGTCAAATTGATGGAAATATAAAGGATATTATAGGGAGGATGCCCCAGTGTCCATTGAACAGACTGCANNGGATC
CA

BPB-6976

BPB-3418
TGCAGAAAGTAAGCAAGCAACCAAGTTCAGAGTTTACCATCAGAGGGAGCAGAGGAGGGGCTTACAATCAGCTATGCCAG
AGAGGAATAGAGGTAGCCGGGCGAGGCTGAGGTGACAGTTGGACGGACGCGTGCTTCCAGCTGGATACATATAAAGCTTG
ATATCTATTCGTACCGTACTGAATTCTACATGAGCTTGTCAGCTACATATATAAAGCTTGCTATCTACTCTCTCCGTTCC
TAAATATAAGTCTTTTAAAAGATTCTACTACGAACTACACACGGATGTATATAGACATATTTTAGAGTGTAGATTCATTC
ATTTTGCTCCATATGTATGGCCGTCCTTCTGATTCAGTTTCCCTACCCACGCCTATCTTCGGCATATTCACAATACATCA
TTGTAATGTAAATTTGCAAATGATCAAAGTGCCCATGTGAATTAGTGTTCCCAAACGTGTAACGTGTAACGTGTAAACAA
GGGAGACTCCCTCAGTTTCCTCGTCGGTTCTCCTCCACTTCCAATCCACTTCGTCGTCCTCCGTGCTATGAAGGCTTTCA
TAGATTCCGACCTGCA

BPB-24334

BPB-3461
CGGATAACAAATTCACACAGGNAACAGNTTATGNNCNTGATTGCGCCAAGCTTGGTGCNGAGGTTGGATCCACTAGTAAC
GGCCGCCAGTGTGCTGGAATTCGCCCTTGAGGGATCCAGTGCAGCATCTTGATCTCTCATGTCATTTTTACAGAGAAGGA
ATGTACTCAAGAGACATTTCATGGGTAGCGCGCATTCCTAATTTGCAGTCTCTTGGAATGAATGGGGTAAACCTCAGCAC
GGTAGTTGATTGGCCTTATGTTATCAATATGATCCCTTCCTTGAAGGCCCTCAGTCTCCAGTCTTGCTCTCTTCCAACCG
CAAATCAATCACTCCCACATATTAATAACCTTACCGAACTGGAGAGGCTTGATCTCTCTGGCAACATCTTTGCCCACCCA
ATGTCAAGGGGTTGGTTTTGGAATTTGACAGGCCTCCAGCATCTTTACCTTGCCGGCACTCTACTGTACGGTCAAGCACC
TGATGCACTGGCACGTATGACGTCCCTTCAAGTCCTTGATTTGTCAGGTAATCGTGACATGGGGATGATGAGTAGTACAA
GCTTAAAGCACCTATGCAGTCTGAAAATTCTGGACCTCTCTTTTTGTCAAATTGATGGAAATATAAAGGATATTATAGGG
AGGATGCCCCAGTGTCCATTGAACAGACTGCA

BPB-4808
TGCTCGAGCGGCCGCCAGTGTGATGGATATCTGCAGAATTCGCCCTTGTAGACTGCGTATCCGGATCTCGGCTTGCTTGG
AAGGTTGCCGGCCCTTCATACTCTGCTGTTAAAGAGCCTCCGCCATG

BPB-1285
CATGTATAATCAGCCCTCCTTCAAGCTGTGTCAATGCCCCAAGTTCTGTCAGTTCAAATCCCATGAGGGAGAGGACAGAA
AGGGAGGAGGGAGGACGCACGGGGGACTGATCCGGATACGCAGTCTACGAAGGGCGAATTCCAGCACACTGGCGGCAATT
ACTAGTGGATCCGAGCTCGGTACCAAGCTTGGCGTAATCATGGTCATAGCTGTTTCCTGTGTGAAATTGTTATCCGCTCA
ANNT

BPB-4552
TGCAGCATACTCAACAAAATGTATTACTTATTCAGTCTTCACAGGCCGGCTTTATACCAAAAGATACCACTACTTGTAGC
ATCTGTATGTGCATGGTGTGGCAATGTCTTTTGCTCAATGTCTTCGGTCACATCTATGGTGTTGCAATCTTTTTGCTAAA
CAACATGCCTATGCTTGTGCTTTCCCAGCTGTCGTGTTGCTACAAATTTTTGAAGGACGACTACAAGAAACTACACTCAA
TTGATGCAACCGCTCTTTATAGAATTGCGCTATCATCGGATCCCGATGCGGTTGTTAGCGTCAGTGGCAGTGATTCCGTG
TGTTCAGACGGCCCAGCAAACAACAGCCATAGCCAAATGACGTCAAGCGAAAACGAAAGCATATGCAGGATCGGTAGGTC
TTACTCCTAGTGCTGCTTAGGAGTAAAAAGTTCAGTACACATGTTTCATTTTCCCATAGCACACATACAATTACTATCAT
GTGTTTTTAACCTGCTGCTGCCCAGTAGTCCAATGCAAATTGCTGGTCTTAGCCTTCACAACTCAAAGTCAACTAAGTAA
CCGCTCAAACAGTTAAGGTGCTGCTTCTTTTTGGGAAGCATTGAAGCACAACTCTTAACAGAAATGTTAGCAACGGTCTG
CA

BPB-6109
TGCAGACCGTTGCTAACATTTCTGTTAAGAGTTGTGCTTCAATGCTTCCCAAAAAGAAGCAGCACCTTAACTGTTTGAGC
GGTTACTTAGTTGACTTTGAGTTGTGAAGGCTAAGACCAGCAATTTGCATTGGACTACTGGGCAGCAGCAGGTTAAAAAC
ACATGATAGTAATTGTATGTGTGCTATGGGAAAATGAAACATGTGTACTGAACTTTTTACTCCTAAGCAGCACTAGGAGT
AAGACCTACCGATCCTGCATATGCTTTCGTTTTCGCTTGACGTCATTTGGCTATGGCTGTTGTTTGCTGGGCCGTCTGAA
CACACGGAATCACTGCCACTGACGCTAACAACCGCATCGGGATCCGATGATAGCGCAATTCTATAAAGAGCGGTTGCATC
AATTGAGTGTAGTTTCTTGTAGTCGTCCTTCAAAAATTTGTAGCAACACGACAGCTGGGAAAGCACAAGCATAGGCATGT
TGTTTAGCAAAAAGATTGCAACACCATAGATGTGACCAAAGACATTGAGCAAAAGACATTGCCACACCATGCACATACAG
ATGCTACAAGTAGTGGTATCTTTTGGTATAAAGCCGGCCTGTGAAGACTGAATAAGTAATACATTTTGTTGAGTATGCTG
CA

BPB-8103
TGCAGACCGTTGCTAACATTTCTGTTAAGAGTTGTGCTTCAATGCTTCCCAAAAAGAAGCAGCACCTTAACTGTTTGAGC
GGTTACTTAGTTGACTTTGAGTTGTGAAGGCTAAGACCAGCAATTTGCATTGGACTACTGGGCAGCAGCAGGTTAAAAAC
ACATGATAGTAATTGTATGTGTGCTATGGGAAAATGAAACATGTGTACTGAACTTTTTACTCCTAAGCAGCACTAGGAGT
AAGACCTACCGATCCTGCATATGCTTTCGTTTTCGCTTGACGTCATTTGGCTATGGCTGNTGNTTGNTGGGCCGNCTGAA
CACACGGAATCACTGCCACTGACG

BPB-49307

BPB-2678

BPB-8558
TGCAGATGATAATCAGCTCAAATTGGATTCCTCCCTTTAAATTGAAGTTGGCCTATTTCCCTTCTAGCAAACTTGGACCT
CAGTTTCCTTTGTGGCTTAAAGGGCAGGGAAATATCAGTTATCTTGACATTTCTAATGCAGACATAGTTGACCAACTCCC
AGATTGGTTTTGGGATGTGTTTTCAAATATTCAGTATCTGAACATCTCTTGTAATCAAATCAGTGGCTGGTTACCGAGTA
CGTTGGAATTCATGTCTTCAGATGCGGGTATCATATTTGACCTCAGCTTCAACAACGTCACTGGTGTTTTACCTCAGTTA
CCAAGGCATTTGGTCGAACTTGACATTTCCAGAAACTCATTATCAGGGCCACTACCACAAAATTTTGGAGCTCCATTCCT
TGGTGATTTGTTGCTTTCAGAGAACAGTATCAATGGCACTACTCCCATACATATCTGTGAGCTGCA

BPB-0714
TGCAGATGATAATCAGCTCAAATTGGATTCCTCCCTTTAAATTGAAGTTGGCCTATTTCCCTTCTAGCAAACTTGGACCT
CAGTTTCCTTTGTGGCTTAAAGGGCAGGGAAATATCAGTTATCTTGACATTTCTAATGCAGACATAGTTGACCAACTCCC
AGATTGGTTTTGGGATGTGTTTTCAAATATTCAGTATCTGAACATCTCTTGTAATCAAATCAGTGGCTGGTTACCGAGTA
CGTTGGAATTCATGTCTTCAGATGCGGGTATCATATTTGACCTCAGCTTCAACAACGTCACTGGTGTTTTACCTCAGTTA

CCAAGGCATTTGGTCGAACTTGACATTTCCAGAAACTCATTATCAGGGCCACTACCACAAAATTTTGGAGCTCCATTCCT
TGGTGATTTGTTGCTTTCAGAGAACAGTATCAATGGCACTATTCCCATACATATCTGTGAGCTGCA

BPB-1645
CCAGTGCAGCTGTGGCACGCACGGTGTGTACCACAGAGCAAAAGGATTGCGGAGCAAAGTGACTCACTCGAGTGACAACT
TCTATGCTTCTACTCCCTCAGTTTTTAAATATAAGACCTTTTAGAGATTTTATTATAGATTAGATACAAAGTAAAATGAG
TGAACCTACATCTTAAAATAAATGTATGTAGTTCATAGTAAAAATCTCTAAAAGGTCTTATACTTCATTTAGGAACGGAT
GTAGTATATATTAACCTAGGAGAAGCATGCCAAGGATGAAGATACGGGCCACCAGTCGACCGCATGCCATACTATGTTAC
CTACAAGACTCAACCCTTACACATACAGATGAACTGACACGGTCCTGCACTAAACAAAGGGCAGTGATGACTAAGCAAAT
ATCGTTGAAAGTCTGAGATAAATTTAAAAATAATGCAAGCATCAATGTTAAATTTAGGACTTGATTAAATTTAGGACTTG
AGCCCTGACGAGATGAGGATATCACTATTCTTCTAACCATCCAACCACATTGCTGCTTCGTAGAAGTTGTATTCGATTTA
ATGCTTGGAGGGGCTCACTTCTGCTGCCGGTGCGGGCAAGAATGCCACGAGGAGCAACAGCAGCGCCAGGATGGCGGCGA
GGAGGGGGGAGGCCGCCATCTCTTCCCGTCACTCCCTGCACTGGATCCNTCNAGGGGCGAATTCTGCAGATATCCNTCNN
NNNTGGCNGNNNCTCGAGCATGCATCTAGAGGGCCCAATTCGCCCTATAGTGAGTCGTATTA

BPB-2191
TGCAGATCTTCTCCGTCGAGGTCACGGAGCTCAGCGGGGGCCTCCAGTGGCCGCTCGAGGTCTACGGCGTGGTCGCCACC
CGAGACTCGGTGGACCATAATCGCAACGTCATCTTCAGGCGCAGGAGGGACGCCTCCCTCGTCCTCACCCAACAGGTTTG
TTTTTCCTCTCAACCATCATCTGTAGACTGTAGCTGACATTGGTGTGGTATTCTGTTTTCCGGATGCAATCGGTTGAAGT
TCATCTGTTAACGTGTGCAACTGCTGACTGCA

BPB-4312
TGCAGCATGTCAGAACAATGTATAGCCTGAGGCAGAGGTAGTGATCTGTGTTCATGGGAAGAAATCCGTAGTATGGGACT
GGGGAGTGGGGAAACGTGGTTGGTTACACAGGCTTCTGGCCCTAGCGCAATAATCATCTGGGTCGTTAGGATTAGTTTAC
CTTATAATACTAGGGAGCTGTTTGGCAGCCCTCCACGGAGCGGAGCGCGCTGTTAATTAACAGCTCCGCAAAATATTGCT
GGATGCGCGCGCGACTCCGCTCCAACGCGCAGTAAATTGACAGAAACCGCACTAACAATATGCCATTTGTGTGTTGCAGG
CCCCGCTATACGTGGACACGAAGATGGTTATGAGCATGGTAACGTGGGGAGGCTCTGTGTCACAGGTGATCATGCCAACA
CCAGACGCGTACGCCAGTGCGGGGGCAAGCTGGATCGGGCACTGCCGCCCGGTCTGCCTGCCCAACTTGGGCCACCGACT
GCA

BPB-7382
TGCAGAGATGGAACACGGATAATTTNGGGTGATGAAACTAANTGCGGAGATTAATACACCTATGATGGCATACACGATTT
ACACTCTTTGAATTCTCTTAGGTGCCCAAAACACTCGGACGGTGGGAGTTGCTAGTTACAAAAAAAATGATGTATTTATT
AATATTGATCGATCTATTTTGGAATAACGCAAATTCAGTTTAGTACAAAAAATGCAACTTGCACGTTTAAGAAAGAATTG
TAGCTGCATGTAGTGTGCATATTAGAAGAAAAAATTCAATCAACTGGTGGACCTAACAATAAATGCATATTGCACGTATG
CCCTTTGTGGTTAAACAGTGCAAGCATTGATGCTAGCCGCTCCATGCCCACGCCCACTTCTCACACAATCCTTTGCTTCT
TCGTTATAAATATGTGTTCAGCAATCTAAGCACGAAATATAAGGCTCTCAATTTTCTCTTATTGATTCATCTACAATTTT
TCAAGCCGTTCCGGCCACCACCGCCGCCATGGTACCCTCTTAATTTTTCTGTGCGTAGCTACAGTAACCACACTCCTATA
CCTCATGAGCCGTTCACGCACGGTATACCTTGTTGATTATGCGTGCTTTCGGTCTCACTCGAACTACCGCATCAGCAAGG
CTGAATGGATTGAGAACATTCACCACTCCCGGTCGTACGACGACAGCGGCAAGCATCGCTTCCTGACCCGTATTTCTGAA
CGGTCAGGCCTTGGTGATGAGACCTACCTCCCACCTTACCATCATCATATCCCGCCGTATTATTATTTAAGTGAAGCCCG
TGCTGAGGCTGAGTTGTCTATCTTCACGACCATAGATGATTTGCTCCTGAAGACGTGTATCGACCTTGATGCAATCGCCA
TACTCGTCGTGAACTGCA

BPB-8660
TGCAGTCGGTGGCCCAAGTTGGGCAGGCAGACCGGGCGGCAGTGCCCGATCCAGCTTGCCCCCGCACTGGCGTACGCGTC
TGGTGTTGGCATGATCACCTGTGACACAGAGCCTCCCCACGTTACCATGCTCATAACCATCTTCGTGTCCACGTATAGCG
GGGCCTGCAACACACAAATGGCATATTGTTAGTGCGGTTTCTGTCAATTTACTGCGCGTTGGAGCGGAGTCGCGCGCGCA
TCCAGCAATATTTTGCGGAGCTGTTAATTAACAGCGCGCTCCGCTCCGTGGAGGGCTGCCAAACAGCTCCCTAGTATTAT
AAGGTAAACTAATCCTAACGACCCAGATGATTATTGCGCTAGGGCCAGAAGCCTGTGTAACCAACCACGTTTCCCCACTC
CCCAGTCCCATACTACGGATTTCTTCCCATGAACACAGATCACTACCTCTGCCTCAGGCTATACATTGTTCTGACATGCT
GCA

BPB-6747
TGCAGTTCACGACGAGTATGGCGATTGCATCAAGGTCGATACACGTCTTCAGGAGCAAATCATCTATGGTCGTGAAGATA
GACAACTCAGCCTCAGCACGGGCTTCACTTAAATAATAATACGGCGGGATATGATGATGGTAAGGTGGGAGGTAGGTCTC
ATCACCAAGGCCTGACCGTTCAGAAATACGGGTCAGGAAGCGATGCTTGCCGCTGTCGTCGTACGACCGGGAGTGGTGAA
TGTTCTCAATCCATTCAGCCTTGCTGATGCGGTAGTTCGAGTGAGGCCGAAAGCACGCATAATCAACAAGGTATACAGTG

CGTGAACGGCTCATGAGGTATAGGAGTGTGGTTACTGTAGCTACGCACAGAAAAATTAAGAGGGTACCATGGCGGCGGTG
GTGGCCGGAACGGCTTAAAAAATTGTAGATGAATCAATAAGAGAAAATTGAGAGCCTTATATTTCGTGCTTAGATTGCTG
AACACATATTATAACGAAGAAGCAAAGGATTGTGTGAGAAGTGGGCGTGGGCATGGAGCGGCTAGCATCAATGCTTGCAC
TGTTTAACCACAAAGGGCATACGTGCAATATGCATTATTGTTAGNTCCACCAGTTGATTGAATTTTTTCTTCTAATATGC
ACATTACATGCAGCTACAATTCTNTCTTAAACGTGCAAGTTGCANNNTTTGTACTAAACTGAANNNGCGTTATTCCAAAA
TAGATCGATCAATATTAATAAATACATCATTTTTTTTGTAACTAGCAACTCCCACCGTCCGAGTGTTTTGGGCACCTAAG
AGAATTCAAAGAGTGTAAATCGTGTATGCCATCATAGGTGTATTAATCTCCGCAATTAGTTTCATCACCCAAAATTATCC
GTGTTCCATCTCTGCACTGGA

BPB-2533
TGCAGGACTATGCCTAGGCCTATCAAGAAACAAACTGGCCTGATTATTTCACCATGAAGAAGCAACATGGCACCGCGAAA
CTGCCCAGTAGAGGACCATGAAACATGTGGAAAACAAAGGATAACTTTTTTGCAATGGACATCTAAGAGAACTCTAAAAA
TGATTCTTACCATTGGCCCAGCAACTTCATCAGCCTTTTCTTTGCCATACAGTTGTTCAACCAAAACAATAGAATACTCC
ATGGTTGTTCCTGTCCCACGGCTAGTCACACAATTCCCATCAATCTGTAACCTTGATTCTACAGCTTGCACCTCTGAAGG
AAGTTTGTTCATGCACGGCGGATGACAGGTTGCCTATCACAAACACTCAAATGAGCTGATAATACAAAGCATACAGAAAA
TGAATAATGGGTAAAATTGTAACCTTTAATACATTGAGCAAACCCCAAGCTCCCAGTGCCACAGCTGGTGCAGCACATAC
TGCA

BPB-5852
GGGCCCTCTAGATGCATGCTCGAGCGGCCGCCANTGTGATGGATATCTGCAGAATTCNCCCTTGATGGATCCNNNNNNNN
NNNNNNNNNNNNNNNNNNNNNNNNNNNNNNNNNNNNNNNNNNNTGCAGTATGTGCTGCACCAGCTGTGGCACTGGGAGCT
TGGGGTTTGCTCAATGTATTAAAGGTTACAATTTTACCCATTATTCATTTTCTGTATGCTTTGTATTATCAGCTCATTTG
AGTGTTTGTGATAGGCAACCTGTCATCCGCCGTGCATGAACAAACTTCCTTCAGAGGTGCAAGCTGTAGAATCAAGGTTA
CAGATTGATGGGAATTGTGTGACTAGCCGTGGGACAGGAACAACCATGGAGTATTCTATTGTTTTGGTTGAACAACTGTA
TGGCAAAGAAAAGGCTGATGAAGTTGCTGGGCCAATGGTAAGAATCATTTTTAGAGTTCTCTTAGATGTCCATTGCAAAA
AAGTTATCCTTTGTTTTCCACATGTTTCATGGTCCTCTACTGGGCAGTTTCGCGGTGCCATGTTGCTTCTTCATGGTGAA
ATAATCAGGCCAGTTTGTTTCTTGATAGGCCTAGGCATAGTCCTGCA

BPB-2373
TGCAGCTTATCTTCTTAGAGGGAGATAGGCCAGAGTCGGGGAGAAGTGAGACACGCGAGGCCTTCCGCGTCGTTCCGCTG
ATCCCGGGGTACGAGCGCATTCTGGATCTAAGAGGAATATGCCATTCTAGGGAAGGAGTGGGTTCTGGTTCCTCGACGAG
ACGAAACACGAGAATGACCCTTAGGAACGGAAAGTACCCGTTATATTCCATCCGATGATCTGAACCAAAAAGATCAAAAT
GATCATCCGTTGAGAAAAGGATCACCCTAAGATGATCATCTCATGGCTATTGGAAATGAATCAAATTAGATGGTTCTATT
TCTCAACCTTTCTGACCTGCTCCTATGGAACCAAGGTCGAAAGGATTGGAAAAGTCATTCATTCACAAGGACTCATGAAG
GGTTCCTCAAAAAAGGTAAGGATTAGTAGTTCTTTTTTGACATCGATTTCAGAAATGAATGGATTCGGTCCTGTCATACA
TACCGAAATCTCCCACTTGAAGACTATGCCGCTGAAAAGCCATGTATTTAGTTGTAATGAATGGAGAAGCTAGCTAGGTA
GCTACCAGCTAATTATATTGTTAAACCATGTGCCCTCATATGACATTAGGCAACTGTTGGGAGAGGAGCTATCTGGTACC
ACTGTTCAAGATCTGCA

BPB-9181
CTCGGANCCNCTAGTAACGGCCGCCAGTGTGCTGGAATTCGCCCTTTCGTAGACTGCGTATCCGGATCCAGTGCAGCTAT
GGCAGTTTAGGCAGCTACCGCTTTGGTTGGGTTCGGGTCCCTGTCTCACATG

BPB-4646
TGCAGCTATGGCAGTTTAGGCAGCTACCGCTTTGGTTGGGTTCGGGTCCCTGTCTCACATGTTGGTGTATGGCCGATGAG
ACGTTTCCATCTGGGAAGGTCCAAGTGAGACTCTGGCATCCTTTCAGTGAATGAAAAACAGTAAAAAGACCCAATAAGAA
ACACTGAGCATCTCGGCAAAGGAACTTAAATATTGCCAAACTGATCTCACAAAGTAACCGAAAGTTAATTGTAGTGCAAG
TCTGTAACTGTACAATATGTCAATATCCAGCTTCTTGAACTGTCCGGACGTATAGTTTTGCACAATATTTCATTTGCGTT
GATGCAATTTTTCAGCCCCTTGGTTAAAACTTAAAAGGCAGAATAGACACACAAGTTCAACTGCA

BPB-4541
TGCAGTTAAATGTGAACCCCCCACCGGCCATCTGAAGATAATTATTTCCATTTGCCGGATAAGGGACATTTGCTGAATAGT
TTGGCATAGCCATGGGTGGGACATAAACTGGAGAATACAGAGGATGTCGATATGGCATGAAATTCGGATAATGCTGCACA
TGCATTGGAGGGTACATCTGTGCTGCTTGTTGTTGCTGGGACATATGTTGCTGTTGTGTAGACATTGCAATAGGGCTGCT
GCTCGCAAGCTGTGAACTTAGAGCCTGCAAAAACACAACAGGTGAAAGATACCATGAGATAACAGTACTTGCGATCAAGA
GATGAGTTGCTGGGCTTGCTTGGCACATGATAGGGGAGAAAGTCAGTGGGTTAGTTATTTTTTCTTCCCTCCGTGAAGCA
CAGCCACCAAACATATACTCCCTCCGTTCACAAATATAAGATGTTCTAACTTTTTTCTGAATCGGACATATATAGACACA

TTTTAGTATGTTCGTTCACTCATTTCAGTCCATACGTAACCATATTGAAATATCCAAAACATCTTATATTTCTGAACGGA
GGGAGTACCTATCCCGCCCCCAATGTTCCAGATCTGCTGGGGCCACTTCACTATTCCTGCA

Supplemental Figure S1. The sequences of the relevant DArT makers that are associated with the QTLs in RIL population derived from the cross between Xena X H94061120.
